# Linear growth in preschool children treated with mass azithromycin distributions for trachoma: A cluster-randomized trial

**DOI:** 10.1371/journal.pntd.0007442

**Published:** 2019-06-05

**Authors:** Jeremy D. Keenan, Sintayehu Gebresillasie, Nicole E. Stoller, Berhan A. Haile, Zerihun Tadesse, Sun Y. Cotter, Kathryn J. Ray, Kristen Aiemjoy, Travis C. Porco, E. Kelly Callahan, Paul M. Emerson, Thomas M. Lietman

**Affiliations:** 1 Francis I. Proctor Foundation, University of California, San Francisco, San Francisco, California, United States of America; 2 Department of Ophthalmology, University of California, San Francisco, San Francisco, California, United States of America; 3 The Carter Center Ethiopia, Addis Ababa, Ethiopia; 4 Department of Epidemiology & Biostatistics, University of California, San Francisco, San Francisco, California, United States of America; 5 The Carter Center, Atlanta, Georgia, United States of America; 6 Institute for Global Health, University of California, San Francisco, San Francisco, California, United States of America; King Saud University College of Medicine, SAUDI ARABIA

## Abstract

**Background:**

Mass azithromycin distributions have been shown to reduce mortality among pre-school children in sub-Saharan Africa. It is unclear what mediates this mortality reduction, but one possibility is that antibiotics function as growth promoters for young children.

**Methods and findings:**

24 rural Ethiopian communities that had received biannual mass azithromycin distributions over the previous four years were enrolled in a parallel-group, cluster-randomized trial. Communities were randomized in a 1:1 ratio to either continuation of biannual oral azithromycin (20mg/kg for children, 1 g for adults) or to no programmatic antibiotics over the 36 months of the study period. All community members 6 months and older were eligible for the intervention. The primary outcome was ocular chlamydia; height and weight were measured as secondary outcomes on children less than 60 months of age at months 12 and 36. Study participants were not masked; anthropometrists were not informed of the treatment allocation. Anthropometric measurements were collected for 282 children aged 0–36 months at the month 12 assessment and 455 children aged 0–59 months at the month 36 assessment, including 207 children who had measurements at both time points. After adjusting for age and sex, children were slightly but not significantly taller in the biannually treated communities (84.0 cm, 95%CI 83.2–84.8, in the azithromycin-treated communities vs. 83.7 cm, 95%CI 82.9–84.5, in the untreated communities; mean difference 0.31 cm, 95%CI -0.85 to 1.47, P = 0.60). No adverse events were reported.

**Conclusions:**

Periodic mass azithromycin distributions for trachoma did not demonstrate a strong impact on childhood growth.

**Trial registration:**

The TANA II trial was registered on clinicaltrials.gov #NCT01202331.

## Author summary

Mass distribution of a single dose of the broad-spectrum antibiotic azithromycin twice per year to pre-school children in Sub-Saharan Africa has been shown to reduce childhood mortality. The mechanism by which azithromycin reduces mortality is currently not clear, especially since the antibiotic is not targeted to sick children but rather given to all children in the community whether or not they have an infectious disease. In this study, we report the height and weight of children enrolled in a trial in Ethiopia in which communities were randomized either to twice annual mass azithromycin distributions for blinding trachoma or to no treatments. After accounting for age and sex, children from azithromycin-treated communities were on average slightly taller at the 12- and 36-month study visits than those from untreated communities, but the difference was not statistically significant.

## Introduction

Undernutrition is thought to contribute more to the global burden of disease than any other risk factor.[1] Poor nutrition potentiates the effects of infections such as diarrhea, respiratory infections, and malaria, leading to worse outcomes and higher mortality.[2, 3] Infectious diseases in turn lead to poor growth.[4] It is conceivable that antibiotics could have an important role for breaking this cycle of malnutrition and infection. For example, a randomized trial demonstrated less stunting and underweight in HIV-infected children who took daily co-trimoxazole compared to those taking placebo.[5] This idea is not new; antibiotics have long been thought to be effective growth promoters for animal husbandry.[6]

Mass azithromycin treatments have recently been shown to reduce childhood mortality in sub-Saharan Africa, although the causal pathway for the mortality reduction is unclear.[7, 8] Azithromycin is a broad-spectrum antibiotic with efficacy against a wide array of pathogens that cause respiratory disease, diarrhea, and malaria, so it is possible that azithromycin prevents mortality by directly clearing these infections.[9–11] Alternatively, if azithromycin caused height and weight gain in children, it is possible that the improved growth could be partly responsible for the survival benefit.

In a recent cluster-randomized trial, we randomized communities that had been treated with four years of mass azithromycin for trachoma to either continued antibiotics or cessation of treatment. Cluster-randomization, which was chosen for the primary trachoma outcomes, allowed assessment of both the direct and spillover effects of antibiotics.[12] We recognized that this trial provided an opportunity to assess whether mass azithromycin distributions had an effect on childhood growth, and consequently added height and weight as secondary outcomes. We compared anthropometric measurements at the individual level to determine whether mass azithromycin increased childhood growth.

## Methods

### Ethics

The study had approval from the University of California, San Francisco; Emory University; the Ethiopian Ministry of Science and Technology; and the Food, Medicine, and Health Care Administration and Control Authority of Ethiopia. The study was carried out in accordance with the Declaration of Helsinki and overseen by a Data Safety and Monitoring Committee appointed by the National Institutes of Health-National Eye Institute. Verbal informed consent was obtained in Amharic from community leaders before randomization and from the guardians of all children post-randomization; verbal consent was approved by all institutional review boards and was used due to the high levels of illiteracy in the study area.

### Study design

This study describes a secondary outcome from a parallel-group cluster-randomized clinical trial for trachoma (TANA II; clinicaltrials.gov #NCT01202331) performed in the Goncha Siso Enese *woreda* (district) of the Amhara region in Ethiopia.[13] The trial was conducted in communities that had been treated with 8 biannual mass azithromycin distributions for trachoma as part of an earlier trial (TANA I; clinicaltrials.gov #NCT00322972).[14] In the initial TANA I trial (June 2006 until November 2009), 72 contiguous *subkebeles* (sub-districts) were randomized to 1 of 6 different trachoma treatment strategies.[14] Rural subkebeles were eligible for enrollment if they were located within a 3-hour walk from the farthest point accessible to a four-wheel drive automobile. As part of the TANA I trial, 12 subkebeles were randomized to receive biannual mass azithromycin distributions (every 6 months, ±1 month). Each subkebele consisted of approximately 4–6 government-defined demographic units known as state teams; all state teams in the subkebele were treated identically. In TANA II (November 2010 until May 2013), two randomly selected state teams from each biannually treated subkebele were randomized to either continued biannual mass azithromycin (N = 12 state teams) or cessation of antibiotics (N = 12 state teams). Anthropometric indices were measured as a secondary outcome in these 24 state teams 12 and 36 months after randomization, providing a randomized controlled trial comparison of childhood growth in communities treated with mass azithromycin distributions versus no treatment. No changes were made to the anthropometry portion of the study after its addition to the main trial. The trial was reported according to CONSORT guidelines (S1 Checklist). Details of the study design were pre-specified in a trial protocol (S1 Protocol).

### Randomization and masking

This study employed pair-matched cluster-randomization with matching based on the TANA I subkebele. A biostatistician (TCP) used the statistical package R (R Foundation for Statistical Computing, Vienna, Austria) to randomize 12 pairs of state teams from each of 12 subkebeles, with one member of the pair allocated to intervention and the other to control. A study coordinator enrolled the communities and participants and assigned the intervention. Allocation was concealed by enrolling all communities before randomization and administering the intervention to the entire community population. Although study participants were not masked to their cluster’s treatment assignment, the study personnel responsible for anthropometric assessments were not informed of the treatment allocation or study hypothesis.

### Intervention

**Fig 1:**
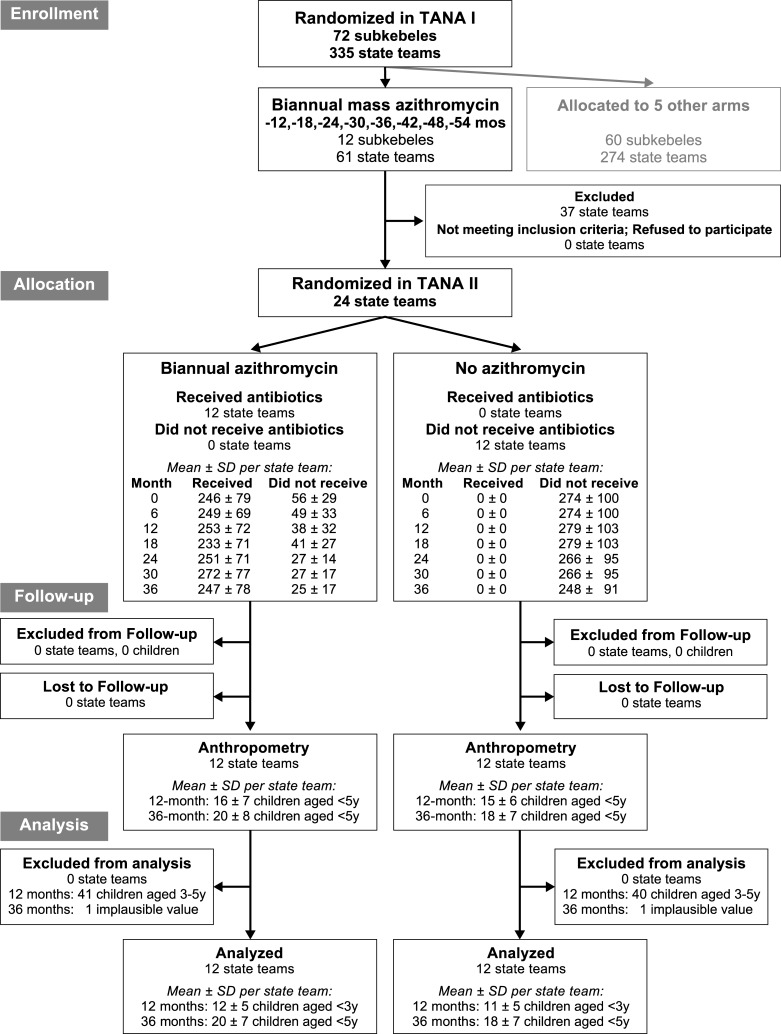
Participant flow. Communities were randomized twice: originally as part of the TANA I trial (2006–2009), and subsequently as part of TANA II (2010–2013).

Local health extension workers performed a population census each year to enumerate all community members eligible for treatment. Because birthdates are imprecise in this part of Ethiopia, census-takers recorded age in years. During scheduled mass treatment visits, these same health workers offered a single dose of directly observed oral azithromycin (20mg/kg height-based approximation for children, 1g for adults) to all community members except for children under 6 months of age, self-reported pregnant women, and those known to be allergic to macrolide antibiotics, each of whom were instead offered a six-week course of twice daily tetracycline ophthalmic ointment. Each community was treated twice per year (Fig 1). Antibiotic coverage during mass treatments was assessed from the preceding census. Although study participants from all communities continued to receive routine government health services during this time, no other studies or interventions were performed during the study period. No adverse events were reported through routine passive surveillance activities; active monitoring of adverse events was performed during the trial and is reported elsewhere.[15]

### Outcome assessment

We performed anthropometric assessments after the month 12 and month 36 treatments. All children aged 0–59 months identified from the most recent census were offered height and weight measurements, conducted in a central area in each state team. We recruited anthropometrists from the local communities, and conducted a 2-day training session using methods recommended by the World Health Organization (WHO). Anthropometry teams demonstrated high levels of reproducibility for all measurements.[16] We used a portable stadiometer (Shorr Productions, LLC, Olney, MD, USA) to measure height or length and a Seca 874 floor scale (Seca GmbH & Co. KG, Hamburg, Germany) to measure weight. Each measurement was taken in triplicate, with the median used as the official measurement.

### Statistics

The pre-specified primary analysis for the anthropometric outcomes was a post-test only comparison of height adjusted for age and sex in children not old enough to be eligible for treatment during the first trial (i.e., aged 0–36 months at the 12-month visit and 0–59 months at the 36-month visit). Repeated measures for height were modeled as a function of treatment arm, time, age in years (as a continuous variable rounded to whole years), and sex in a mixed effects linear regression, with state team and individual modeled as random effects. Heteroskedasticity was modeled by allowing independent residual errors over each year of age. The p-value was estimated with a Monte Carlo permutation test stratified by subkebele to account for the randomization strategy (10,000 permutations of the likelihood ratio comparing models with and without the treatment term). Intraclass correlation coefficients (ICCs) were calculated from the model to assess cluster-correlation of the primary outcome. Similar models were constructed for the secondary underweight analysis (weight adjusted for age and sex). We purposefully did not use z-scores in the primary analysis given the imprecision of reported ages in the study area. For all outcomes, we performed intention-to-treat analyses.

### Sample size

Sample size calculations for the trial were based on the primary trachoma outcomes and set at 12 communities per arm; including 18 children per community would provide 80% power to detect a 1 cm difference between the two arms assuming an average height of 90 cm (standard deviation 10 cm), ICC of 0.02, correlation coefficient of 0.9 for the relationship of height with age and sex, equal cluster sizes, and a two-sided alpha of 0.05.[17] All analyses were performed using Stata 14.2 (Statacorp, College Station, TX) except for permutation tests, which were done in R 3.4.0 (R Foundation for Statistical Computing, Vienna, Austria).

## Results

The study was conducted from November 2010 until May 2013. Characteristics of the two treatment arms were balanced at baseline (Table 1). The biannual treatment group received 7 mass azithromycin treatments during the continuation trial; the control group received none. Table 2 shows the median antibiotic coverage at each distribution for the under-5 age group.

**Table 1:** Baseline characteristics of 24 communities randomized to either continuation or cessation of biannual mass azithromycin treatment.

	Biannual MDA (*N* = 12 communities)	No MDA (*N* = 12 communities)
Persons per community	309 (242–423)	259 (180–360)
Proportion female, %	51.1% (47.6–52.4%)	52.1% (48.6–55.2%)
Proportion <10 years, %	28.3% (26.5–29.5%)	27.2% (25.4–29.8%)
Prevalence of active trachoma, %*	42.1% (13.1–53.6%)	30.9% (20.0–48.9%)
Distance to nearest town, km	8.4 (5.1–9.2)	8.1 (5.0–9.1)
Altitude, m	2622 (2528–2674)	2611 (2545–2657)

Values indicate the median and interquartile range of community-level data. MDA: mass drug administration with azithromycin

* Sub-district prevalence, as assessed from a random sample of children aged <10 years of age

**Table 2:** Azithromycin coverage among population aged 6–59 months of age in 12 biannually treated communities.

Month	No. per community	Antibiotic coverage
0	35 (29–44)	86% (83–91%)
6	34 (27–42)	93% (91–94%)
12	29 (21–40)	91% (86–95%)
18	29 (20–42)	91% (89–98%)
24	29 (21–41)	82% (76–86%)
30	28 (21–41)	94% (92–96%)
36	27 (17–41)	87% (83–89%)

Values represent median and interquartile range of community-level data.

We obtained anthropometric measurements on 282 of 387 (73%) eligible children aged <3 years at the 12-month visit and 455 of 591 (77%) eligible children aged <5 years at the 36-month visit, with 207 children having measurements at both visits. As shown in Table 3, children who were eligible but did not participate in the anthropometric assessment tended to be younger than those who participated.

**Table 3:** Characteristics of study participants at each monitoring visit, stratified by participation status.

	Biannual MDA		No MDA	
Characteristic	Participating	Not participating	Participating	Not participating
Month 12	N = 146	N = 68	N = 136	N = 37
Female	77 (52.7%)	40 (58.8%)	66 (48.5%)	18 (48.7%)
Age				
0 y	32 (21.9%)	32 (47.1%)	23 (16.9%)	11 (29.7%)
1 y	62 (42.5%)	17 (25.0%)	50 (36.8%)	10 (27.0%)
2 y	52 (35.6%)	19 (27.9%)	63 (46.3%)	16 (43.2%)
Month 36	N = 235	N = 71	N = 220	N = 65
Female	121 (51.5%)	36 (50.7%)	104 (47.3%)	32 (49.2%)
Age				
0 y	15 (6.4%)	11 (15.5%)	15 (6.8%)	7 (10.8%)
1 y	52 (22.1%)	18 (25.4%)	28 (12.7%)	7 (10.8%)
2 y	44 (18.7%)	13 (18.3%)	52 (23.6%)	15 (23.1%)
3 y	64 (27.2%)	17 (23.9%)	62 (28.2%)	19 (29.2%)
4 y	60 (25.5%)	12 (16.9%)	63 (28.6%)	17 (26.2%)

The mean height and weight in each treatment arm at the two time points are shown in Table 4, stratified by age. After adjusting for age, sex, and study visit, the mean height was 84.0 cm (95%CI 83.2–84.8) in the azithromycin-treated communities and 83.7 (95%CI 82.9–84.5) in the untreated communities. In the primary pre-specified analysis adjusted for age, sex, and time point, children in the azithromycin-treated communities were on average 0.31 cm taller (95%CI -0.85 to 1.47) than those in untreated communities (P = 0.60). The ICC derived from the main statistical model suggested mild clustering of height measurements within communities (ICC 0.05, 95%CI 0.02 to 0.13).

**Table 4:** Anthropometric assessment stratified by follow-up visit and age.

		Biannual Mass Azithromycin			No Mass Azithromycin	
Age	N	Height, cm	Weight, kg	N	Height, cm	Weight, kg
Month 12						
0 y	32	66.6 (65.1–68.1)	7.1 (6.7–7.5)	23	68.1 (65.3–71.0)	7.3 (6.8–7.7)
1 y	62	76.7 (75.5–78.0)	9.2 (8.9–9.5)	50	77.1 (75.9–78.2)	9.1 (8.8–9.4)
2 y	52	83.6 (82.8–85.1)	10.5 (10.1–10.9)	63	83.9 (82.3–85.6)	10.7 (10.3–11.1)
Month 36						
0 y	15	66.5 (64.9–68.1)	7.4 (7.0–7.9)	15	66.0 (64.5–67.5)	7.3 (6.8–7.7)
1 y	52	74.9 (73.6–76.1)	8.9 (8.5–9.2)	28	75.8 (73.9–77.6)	8.9 (8.5–9.3)
2 y	44	84.8 (82.9–86.7)	10.8 (10.4–11.3)	52	83.7 (82.5–85.0)	10.8 (10.4–11.1)
3 y	64	93.7 (92.1–95.2)	12.7 (12.3–13.1)	62	91.7 (90.3–93.0)	12.4 (12.0–12.9)
4 y	60	99.6 (98.2–101.0)	14.3 (13.9–14.7)	63	99.2 (97.9–100.5)	14.3 (13.9–14.7)

The weight outcome is summarized in Table 4. After adjusting for age, sex, and study visit, the average weight was 10.8 kg in the azithromycin-treated communities and 10.7 in the untreated communities—a non-significant difference (mean weight 0.09 kg heavier in the azithromycin arm, 95%CI -0.20 to 0.39; P = 0.54; ICC 0.07, 95%CI 0.03 to 0.15). Additional sensitivity analyses using all <5 year-old children at both outcome visits (i.e., including children from the 12-month visit who had been treated in the previous trial) had similar results (S1 File).

## Discussion

We failed to find an association between mass azithromycin distributions and childhood growth. Although on average children from communities treated with biannual mass azithromycin distributions over a 3-year period were slightly taller than children from untreated communities, the differences between the two treatment groups were not consistently statistically significant across different regression model parameterizations and of small magnitude.

This trial assessed the hypothesis that azithromycin functions as a growth promoting agent for preschool children. The specific mechanisms through which antibiotics may promote growth are unknown, though several theories have been proposed.[18] Antibiotics could treat or prevent infections that would otherwise divert metabolic resources. Antibiotics may also alter intestinal absorption by clearing pathogenic organisms and changing the composition of the gut microbiome, both of which may ultimately mitigate the role of bacterial infection in environmental enteropathy.[19, 20] Alternatively, antibiotics like azithromycin are known to have direct anti-inflammatory properties, which could potentially affect growth.

It is conceivable that improvements in growth could translate into improved childhood survival. It is thought that growth and nutrition are important modulators of infectious diseases. Better nutrition may enhance the ability of the immune system to clear infections, and thus make a child less susceptible to severe complications of infection. Indeed, growth promotion is one theory for the improved survival observed following mass azithromycin distributions.[7, 8] While this study does not provide evidence that mass azithromycin impacts childhood growth, it is still possible that azithromycin treatments affect growth but at a magnitude smaller than could be detected by the present study. For example, the results of the present study are consistent with a meta-analysis that estimated antibiotics to confer an additional 0.04 cm of linear growth per month.[21]

Previous randomized trials have studied the impact of antibiotics on childhood growth in the context of a specific disease, such as severe acute malnutrition, HIV, diarrhea, cystic fibrosis, or vesicoureteral reflux.[22–25] Notable among these is a placebo-controlled trial of daily prophylactic co-trimoxazole therapy for HIV-infected children in Zambia that found improved linear growth and decreased mortality in antibiotic-treated children.[5] While many of the other studies failed to find an effect of antibiotic therapy on height measurements, they were of relatively short duration and focused on changes in weight instead of height.[22–25]

Other trials have assessed whether community antibiotic distributions affect growth parameters. Two placebo-controlled studies performed in Guatemala, one of which treated school-aged children with aureomycin and another which treated preschool children with metronidazole, found improved height and weight metrics in children treated with antibiotics. However, these studies may be subject to type I error since they allocated a single cluster of children to the antibiotic intervention but performed analyses at the individual level.[26, 27] Several recent cluster-randomized trials testing more frequent vs. less frequent mass azithromycin distribution strategies for trachoma have failed to detect a difference in height or weight between the different dosing groups.[17, 28, 29] The control groups in those studies were not ideal in that they too received antibiotics—albeit at lower frequencies—and thus may have received a benefit of antibiotic therapy. The present study is an improvement over these previous reports in that no programmatic antibiotics were given in the control group, providing a better assessment of the effectiveness of antibiotics alone.

Mass antibiotics provide selection pressure for antibiotic-resistant organisms, which increases the community prevalence of resistance.[30] Although the clinical importance of such resistance is unclear given the low usage of macrolide antibiotics in developing countries, the potential for antibiotic resistance should be taken into account wherever implementation of mass azithromycin distributions is considered.[31]

Several limitations to this study should be noted. We conducted a post-test only analysis with two follow-up visits. Baseline and more frequent anthropometric measurements would have increased the precision and added to the statistical power of the study. Ages were not known with great accuracy in this study, since families in this region of Ethiopia do not keep health cards and do not record dates of birth. Imprecision in age could have biased the study if age was differentially recorded between treatment arms or if cluster-randomization left an imbalance of ages in the two groups. This study was cluster-randomized since it was performed in the context of community antibiotic treatments for trachoma. An individually randomized trial could have been powered to detect an even smaller difference between children treated with and without antibiotics. On the other hand, the cluster-randomization better approximates the effectiveness of programmatic azithromycin and allows for the possibility of both direct and spillover effects of antibiotics.[12] Finally, this study was conducted in a region of Ethiopia with hyperendemic trachoma that had already received several rounds of mass azithromycin distributions. The generalizability of the findings to an antibiotic-naïve population or a population with milder or no trachoma is unclear.

In conclusion, mass azithromycin distributions have proven extremely effective at reducing the burden of ocular chlamydia and may also have important ancillary benefits, like reducing the prevalence of respiratory infections, diarrhea, malaria, and skin infections—as well as childhood deaths.[9–11, 32] Although we hypothesized that childhood growth promotion might be an additional benefit of mass azithromycin distributions, the present study was unable to provide strong evidence of such an association.

## Supporting information

S1 Checklist CONSORT checklist. (PDF)

S1 File Supplementary material. (PDF)

S1 Protocol Manual of procedures and statistical analysis plan. (PDF)
