# Supplementary material for: Linear growth in preschool children treated with mass azithromycin distributions for trachoma: A cluster-randomized trial
Source: PLoS Negl Trop Dis. 2019 Jun 5;13(6):e0007442. doi: 10.1371/journal.pntd.0007442 (PMC6550377; doi:10.1371/journal.pntd.0007442)
Supplement: S1 File — (PDF) [file pntd.0007442.s002.pdf]

Table of Contents

Table	Description	Page
**Supplementary Table 1:**	Crude mean anthropometric values and differences between treatment groups at each time point, main analysis population ……….	2
**Supplementary Table 2:**	Anthropometric assessment stratified by follow-up visit and age, children <5 years at both time points …..……………….…….………..	3
**Supplementary Table 3:**	Crude and adjusted mean anthropometric values and differences between treatment groups, children <5 years at both time points.…….	4

Supplementary Table 1. Crude mean anthropometric values and differences between treatment groups at each time point, main analysis population (i.e., children <3 years at month 12 and <5 years at month 36).

	Azithromycin		No Mass Azithromycin			
Measurement	N	Mean (95%CI)	N	Mean (95%CI)	Difference (95%CI)	P-value
Month 12						
Height, cm	146	77.1 (75.4-78.8)	136	78.8 (77.1-80.6)	-1.74 (-4.14 to 0.67)	0.16
Weight, kg	146	9.2 (8.8-9.6)	136	9.6 (9.2-9.9)	-0.36 (-0.88 to 0.18)	0.19
Month 36						
Height, cm	235	87.6 (86.1-89.1)	220	88.2 (86.7-89.7)	-0.57 (-2.68 to 1.54)	0.60
Weight, kg	235	11.6 (11.2-11.9)	220	11.8 (11.4-12.1)	-0.20 (-0.68 to 0.29)	0.43

Supplementary Table 2. Anthropometric assessment stratified by follow-up visit and age, children <5 years at both time points

	Biannual Mass Azithromycin			No Mass Azithromycin		
Age	N	Height, cm	Weight, kg	N	Height, cm	Weight, kg
Month 12						
0 y	32	66.6 (65.1-68.1)	7.1 (6.7-7.5)	23	68.1 (65.3-71.0)	7.3 (6.8-7.7)
1 y	62	76.7 (75.5-78.0)	9.2 (8.9-9.5)	50	77.1 (75.9-78.2)	9.1 (8.8-9.4)
2 y	52	83.6 (82.8-85.1)	10.5 (10.1-10.9)	63	83.9 (82.3-85.6)	10.7 (10.3-11.1)
3 y	22	91.5 (88.4-94.5)	12.2 (11.4-12.9)	22	89.6 (86.3-92.9)	11.7 (10.8-12.5)
4 y	19	102.8 (98.9-106.6)	15.0 (13.8-16.1)	18	99.7 (96.6-102.9)	13.5 (12.7-14.3)
Month 36						
0 y	15	66.5 (64.9-68.1)	7.4 (7.0-7.9)	15	66.0 (64.5-67.5)	7.3 (6.8-7.7)
1 y	52	74.9 (73.6-76.1)	8.9 (8.5-9.2)	28	75.8 (73.9-77.6)	8.9 (8.5-9.3)
2 y	44	84.8 (82.9-86.7)	10.8 (10.4-11.3)	52	83.7 (82.5-85.0)	10.8 (10.4-11.1)
3 y	64	93.7 (92.1-95.2)	12.7 (12.3-13.1)	62	91.7 (90.3-93.0)	12.4 (12.0-12.9)
4 y	60	99.6 (98.2-101.0)	14.3 (13.9-14.7)	63	99.2 (97.9-100.5)	14.3 (13.9-14.7)

Supplementary Table 3. Crude and adjusted mean anthropometric values and differences between treatment groups, children <5 years at both time points. Crude figures are provided separately for each time point. Adjusted analyses include data from both time points.

	Azithromycin		Placebo			
Measurement	N	Mean (95%CI)	N	Mean (95%CI)	Difference (95%CI)	P-value
Crude						
Month 12						
Height, cm	187	81.3 (79.6-83.0)	176	82.2 (80.7-83.8)	-0.95 (-3.23 to 1.32)	0.41
Weight, kg	187	10.1 (9.7-10.5)	176	10.2 (9.9-10.5)	-0.09 (-0.59 to 0.41)	0.73
Month 36						
Height, cm	235	87.6 (86.1-89.1)	220	88.1 (86.7-89.6)	-0.57 (-2.68 to 1.54)	0.60
Weight, kg	235	11.6 (11.2-11.9)	220	11.8 (11.4-12.1)	-0.20 (-0.68 to 0.29)	0.43
Adjusted*						
Height, cm	422	85.4 (84.5-86.3)	396	84.7 (83.8-85.5)	0.75 (-0.49 to 1.98)	0.24
Weight, kg	422	11.1 (10.9-11.4)	396	10.9 (10.6-11.1)	0.24 (-0.11 to 0.59)	0.18

* Data from both time points modeled in mixed effects regression models with treatment arm, time, age, and sex as fixed effects and state team and individual over time as nested random effects.
