# Supplementary material for: Linear growth in preschool children treated with mass azithromycin distributions for trachoma: A cluster-randomized trial
Source: PLoS Negl Trop Dis. 2019 Jun 5;13(6):e0007442. doi: 10.1371/journal.pntd.0007442 (PMC6550377; doi:10.1371/journal.pntd.0007442)
Supplement: S1 Protocol — (PDF) [file pntd.0007442.s003.pdf]

Version 9

Tripartite International Research for the Elimination of Trachoma

Manual of Operations and Procedures

Francis. I. Proctor Foundation,
The Carter Center,
Bahar Dar Regional Laboratory

Investigators:

Teshome Gebre[2]
Zerihun Tadesse[2]
Paul Emerson, PhD[2]
Sintayehu Gebrisillasie[2]
Berhan Ayele[2]
Nicole Stoller, MPH[1]
Sun Yu, MPH[1]
Vicky Cevallos[1]
Zhaoxia Zhou[1]
Jeremy Keenan, MD, MPH[1]
Travis C. Porco, PhD, MPH (Biostatistician)[1]
Bruce D. Gaynor, MD[1]
John P. Whitcher, MD, MPH[1]
Thomas M. Lietman, MD*[1]

*Principal Investigator

[1]Francis I. Proctor Foundation for Research in Ophthalmology, San Francisco, California, USA;
[2]The Carter Center, Atlanta, Georgia, USA & Ethiopia

TABLE OF CONTENTS

1. Introduction
    1.1 Specific Aims of the Study
    1.2 Study Outcomes
        1.2.1 Main Outcomes
        1.2.2 Other Pre-specified Outcomes
    1.3 Study Design

2. Background and Rationale

3. Organization and Policies
    3.1 Collaborating Institutions
    3.2 Duties & Responsibilities of Staff
        3.2.1 Principal Investigator
        3.2.2 Study Coordinators (Proctor Foundation)
        3.2.3 Study Coordinator (The Carter Center, Ethiopia)
        3.2.4 Co-Investigators (US & Ethiopia)
        3.2.5 Microbiologist (ARSHO)
        3.2.6 Data Coordinator (The Carter Center, Ethiopia)
        3.2.7 Database Specialist (Proctor Foundation & ARSHO)
        3.2.8 Laboratory Technician (ARSHO)
        3.2.9 Biostatistician/Data Analyst at Proctor
        3.2.10 Treatment and Census Team: Local health extension workers from Ministry of Health, Ethiopia
        3.2.11 Collection Team: Local health workers/nurses from Ethiopian Ministry of Health
    3.3 Policy Matters
        3.3.1 Protocol Revisions during the Trial
    3.4 Presentations and Publications
        3.4.1 Authorship Policy
    3.5 Data Safety and Monitoring Committee
        3.5.1 Stopping (& Restarting) Rules

4. Patient Flow
    4.1 Eligibility Requirements
    4.2 Randomization
    4.3 Scheduling Visits
        4.3.1 Baseline Visit
        4.3.2 Treatment
        4.3.3 Follow-up Visits
    4.4 Adherence to Treatment
    4.5 Adverse Outcomes and Patient Death
        4.5.1 Adverse Outcomes
        4.5.2 Patient Death

5. Examination and Procedures
    5.1 Registering Participants for Examination
    5.2 Gloving of the Examiner's Hands
    5.3 Examination Positions
    5.4 Everting the Upper Eyelid
    5.5 Examining the Conjunctiva & Trachoma Grading

5.5.1 WHO Simplified Trachoma Grading Card
5.6 Field Specimen Collection Quality Control Measures
5.7 Examination Station and Diagram

6. Microbiology Laboratory Procedures for PCR
6.1 Specimen Collection for Microbiological Tests (Principal)
6.2 Materials for PCR
6.2.1 Swabs
6.2.2. Sample Tubes
6.2.3 Cooler Bags with Frozen Ice Packs
6.2.4 -20°C Freezer
6.2.5 Latex Gloves
6.3 Training of Examiners
6.3.1 Pre-Monitoring Visit Training
6.3.2 In-Country Training
6.4 Specimen Collection
6.4.1 Protocol for swabbing of the conjunctiva
6.4.2 PCR Control Swabs
6.4.3 Protocol for tubing and handling of samples
6.5 Transport and Storage of Samples
6.6 Methods
6.6.1 Description of the Roche COBAS AMPLICOR™ CT/NG Assay
6.6.2 Processing of Samples for PCR
6.6.3 Procedure for Masking Samples
6.6.4 Procedure for Pooling Samples
6.7 Quality Control
6.8 Laboratory Results Reporting

7. Macrolide Resistance Testing
7.1 Specimen Collection for Macrolide Resistance Testing
7.2 Materials for NP Collection
7.2.1 Nasopharyngeal Swabs
7.2.2 Nasopharyngeal Sample Tubes
7.2.3 Cooler Bags with Frozen Ice Packs
7.2.4 -20°C Freezer.
7.2.5 Latex Gloves
7.3 Training of Examiners
7.4 Nasopharyngeal Specimen Collection
7.4.1 Protocol for Nasopharyngeal Sample Collection
7.5 Transport and Storage of Samples
7.6 Methods
7.6.1 Procedure for Masking Samples
7.6.2 Quality Control
7.6.3 Laboratory Results Reporting

8. Quantification of Ocular Chlamydia

9. Clinical Photography
9.1 Photography Protocol
9.2 Grading of Clinical Photographs

10. Blood Testing
10.1 Fingerprick
10.2 Thick/thin blood smear
10.3 Hemoglobin
10.4 Dried blood spot
10.5 Materials for blood collection

11. Anthropometry
11.1 Height
11.2 Weight
11.3 Mid-Upper Arm Circumference
11.4 Materials for anthropometry

12. Verbal Autopsy

13. Passive Surveillance

14. Study Medication
14.1 Study Medication Description (from Pfizer, Inc.)
14.2 Dosage Information
14.3 Adverse Reactions/Side Effects
14.4 Treatment Costs
149.5 Alternate Therapies (Control Villages)
9.5.1 Tetracycline Ophthalmic Ointment
14.6 Treatment/Monitoring Schedule
14.7 Medication Procurement/Donation
14.8 Study Medication Storage and Accountability
14.9 Medication Quality Control
14.10 Checking Antibiotic Coverage

15. Protection of Human Subjects
15.1 Internal Review Board Approval
10.1.1 UCSF Committee on Human Research
10.1.2 Ethiopian Science and Technology Commission
10.1.3 Emory University Institutional Review Board
15.2 Informed Consent
15.3 Adequacy of Protection Against Risk
15.4 Inclusion of Pregnant Women & Children
15.5 Compensation to Participants

16. Data Collection and Management
16.1 Census administration
11.1.1 Form Description
11.1.2 Filling the Census Form
16.2 Field Data Collection
11.2.1 Registration Form
11.2.2 Field Form
16.3 Data Forms Management
11.3.1 Data Editing
11.3.2 Data Entry and Quality Control
11.3.3 Adjudication of Double Data Entry & Data Entry Errors
11.3.4 Data Consistency & Validity

16.4 Data Security and Storage
16.5 Data Analysis Estimation of Disease Prevalence
16.6 Data Management, Security and Quality Assurance
16.7 Sample Organization and Storage

17. Statistical Analyses
17.1 Calculation of Sample Size & Power Analysis
17.2 Inter-observer Variability

18. Appendix
13.1 Patient Verbal Consent Scripts
13.2 Census Form
13.3 Randomized List for Each Visit (The Carter Center)
13.4 Field Form (Swabbing)

19. References

1. Introduction

In this proposal we begin to address whether the long-term goal for trachoma programs should be control, local elimination, or global eradication. Mass antimicrobial administrations have been remarkably successful in reducing the prevalence of the ocular strains of chlamydia that cause trachoma. Repeated distributions progressively lower the prevalence of infection, and in some cases may even result in local elimination. However, indefinitely continuing mass treatments raises concerns about cost and antibiotic resistance. Thus one of the major remaining questions in trachoma treatment is: **When can we stop mass antibiotics?** The hope has been that adjunctive measures such as latrine construction and hygiene programs would reduce transmission sufficiently such that infection would not return. Unfortunately, no non-antibiotic measure has yet demonstrated an effect on ocular chlamydial infection. Some believe that without other effective measures, infection will return to pre-treatment levels. Others feel that if brought to a low enough level, infection will never return to baseline. The WHO currently recommends that once clinically active trachoma is brought to a low enough level with repeated mass antibiotic treatments, treatment then be targeted to individuals with clinically active disease. Another suggested approach is to target subsequent treatment to the age group thought to be a core group for transmission (pre-school children). Here, we evaluate how infection returns when antibiotics are discontinued, whether infection can be predictably eliminated with continued treatment, and whether infection can be prevented from returning with targeted treatment strategies.

TANA (U10 EY016214) was an NEI-supported collection of cluster-randomized trachoma trials in Ethiopia. In one of the trials, a total of 111 communities have received repeated mass oral azithromycin distributions over 42 months (final visit in Fall 2009). These treatments have lowered, and in some cases may have eliminated, infection. Now, we have a unique opportunity to continue to follow these communities, assessing what measures are necessary, if any, to keep infection from returning. It would take years and a great deal of resources to again set up these conditions in such a controlled setting. Continued monitoring of these communities will allow us to determine the feasibility of control, local elimination, or even eradication. In this proposal, we assess the most appropriate long-term goal for mass antibiotic distributions for trachoma.

Research question:

What is the long term goal for mass antibiotic distributions for trachoma?

1.1 Specific Aims of the Study:

I) Can we stop antibiotics after 3 years? *We hypothesize that infection will return, even from low levels. 24 communities which received repeated mass treatments for three years will be monitored, for 3 additional years.*

II) Can infection be completely eliminated if mass treatments continue for 6 years? *We hypothesize that infection will be completely eliminated in all communities. We will monitor the prevalence of infection in 12 communities which continue to receive annual mass treatments, and 12 which continue to receive biannual mass treatments.*

III) Can treatment targeted to pre-school aged children, or to households in which a pre-school aged child has clinically active trachoma, prevent infection from returning into the community? *We will monitor 12 communities where treatment is targeted to clinically active cases and their households, and another 12 communities where treatment is targeted to pre-school children.*

1.2 Study Outcomes

1.2.1 Main Outcome

Please see SAP. The primary outcome will be the prevalence of ocular chlamydial infection in a community in children 0-9 years old, as estimated from pooled Roche-Amplicor PCR at 36 months.

1.2.2. Other Pre-specified Outcomes

Please see SAP section 2.3.

1.3 Study Design

TANA was an NEI-funded cluster-randomized clinical trial assessing the efficacy of different treatment strategies for trachoma, completed in Autumn 2009. In the current project, entitled TIRET, we will continue to study and monitor a subset of the communities from TANA.

TANA Summary

TANA study questions. TANA was a group-randomized, single-masked, efficacy study designed to determine the following:

1) Is local elimination of chlamydial infection feasible with repeated mass azithromycin distributions, and is the frequency of treatments (biannual vs. annual) important to achieve *elimination*?

2) Do children aged 1-9 years old form a core group? If so, then infection in untreated adults will be reduced in communities where only children have been treated.

3) Does intensive latrine construction prevent the return of infection after a single mass-antibiotic administration?

TANA cluster randomization. The unit of randomization in TANA is the sub-kebele, which is a government-defined unit consisting of 3-5 communities. In TANA, 72 sub-kebele were randomized to one of 6 treatment arms: 4 annual mass treatments of azithromycin, 7 biannual (every six months) mass treatments of azithromycin, 4 quarterly treatments of azithromycin to children only, a single mass treatment of azithromycin, a single mass treatment of azithromycin combined with intensive latrine construction, and a delayed treatment arm, which received a single mass azithromycin treatment one year after the study began (Table 1).

Table 1: TANA Study Design

	A	B	C	D	F	G
	**12 Annual**	**12 Biannual**	**12 Children-0nly**	**12 controls**	**12 Once treated**	**12 Once + latrines**
**Month 0**	Swab And Treat	Swab And Treat	Swab And Treat		Swab And Treat	Swab And Treat
**Month 3**			Swab And Treat			
**Month 6**	Swab	Swab And Treat	Swab And Treat			
**Month 9**			Treat			
**Month 12**	Swab And Treat	Swab And Treat	Swab And Treat	Swab And Treat	Swab	Swab
**Month 18**	Swab	Swab And Treat	*As per Carter program*	*As per Carter program*		
**Month 24**	Swab And Treat	Swab And Treat			Swab And Treat	Swab And Treat
**Month 30**	Swab	Swab And Treat				
**Month 36**	Swab And Treat	Swab And Treat				
**Month 42**	Swab	Swab And Treat				
**Month 48**	Swab And Treat	Swab And Treat			Swab And Treat	Swab And Treat

TIRET Study Design

SA 1:
a) Monitor prevalence of infection in annually treated communities where treatment has ended
b) Monitor prevalence of infection in biannually treated communities where treatment has ended

SA 2:
c) Whole population treated with azithromycin annually
d) Whole population treated with azithromycin twice yearly

SA 3:
f) Treatment targeted to clinically active cases and their households only
g) Treatment targeted to children aged 0 – 5 only

The randomization scheme in this study is complicated, since it involves further cluster randomization of an existing cluster-randomized trial. We will include two treatment arms of the existing TANA trial: the annual treatment arm, and the biannual treatment arm; the following

discussion is limited to these two treatment arms. Study visits have been conducted on these two treatment arms every six months since the Spring of 2006. The first visit of TIRET will occur six months after the final TANA study visit, meaning TANA and TIRET would consist of longitudinal data on chlamydia infection every six months for 7 years. Note that In TANA, the unit of randomization was the sub-kebele, with all communities within the sub-kebele treated identically, but only a sentinel village within each sub-kebele monitored for ocular chlamydia. In TIRET the unit of randomization will be the village; individual communities from TANA will be randomized to one of six treatment arms in TIRET. Specifically, sub-kebles from the annually treated arm will contribute to the following four treatment arms: treatment cessation, continued annual mass treatment, annual targeted treatment based on clinically active trachoma, and annual targeted treatment based on PCR evidence of chlamydial infection. Sub-kebeles from the biannually treated arm in TANA will contribute one village to each of the following two treatment arms: treatment cessation, and continued biannual mass treatment (Table 2). Any villages not randomized for inclusion in TIRET will receive no further treatments, and will be monitored by the Carter Center as part of ongoing trachoma program activities. The treatment groups and study methods will be further described in the following sections.

TANA is a collaboration between the Proctor Foundation, Carter Center, and the Ethiopian Ministry of Health; Carter Center and the Ministry of Health provide local staff for antibiotic distribution, clinical examination, conjunctival swab collection, and latrine construction. We expect TIRET to be similarly structured.

Table 2: Randomization Scheme from TANA to TIRET

			Annual Treatment Arm (N=48 communities)	Biannual Treatment Arm (N=48 communities)
TANA-I	Month 0	Spring 2006	Swab & Treat	Swab & Treat
	Month 6	Fall 2006	Swab	Swab & Treat
	Month 12	Spring 2007	Swab & Treat	Swab & Treat
	Month 18	Fall 2007	Swab	Swab & Treat
	Month 24	Spring 2008	Swab & Treat	Swab & Treat
	Month 30	Fall 2008	Swab	Swab & Treat
	Month 36	Spring 2009	Swab & Treat	Swab & Treat
	Month 42	Fall 2009	Swab	Swab & Treat

Aim 1 Aim 2 Aim 3 Aim 3 | Aim 1 Aim 2

			Annual Treatments in TANA-I				Biannual Treatments in TANA-I	
			Stop - Annual (N=12)	Continue-Annual (N=12)	Target-Age (N=12)	Target-Exam (N=12)	Stop-Biannual (N=12)	Continue-Biannual (N=12)
TIRET	Month 54	Fall 2010	Swab	Swab & Treat	Swab & Targeted Tx	Swab & Targeted Tx	Swab	Swab & Treat
	Month 60	Spring 2011						Swab & Treat
	Month 66	Fall 2011	Swab	Swab & Treat	Swab & Targeted Tx	Swab & Targeted Tx	Swab	Swab & Treat
	Month 72	Spring 2012						Swab & Treat
	Month 78	Fall 2012	Swab	Swab & Treat	Swab & Targeted Tx	Swab & Targeted Tx	Swab	Swab & Treat
	Month 84	Spring 2013						Swab & Treat
	Month 90	Fall 2013	Swab	Bilateral Swab, rRNA	Swab	Swab	Swab	Bilateral Swab, rRNA
			Cost-effectiveness Analysis					

2. Background and Rationale

Trachoma is caused by repeated infection with *Chlamydia trachomatis*. *C.trachomatis* is an obligate intracellular bacterium, unable to synthesize its own ATP.[1] Chlamydia exists in two forms: the elementary body and the reticulate body. The elementary body is the infectious, non-replicating, extracellular form. Once endocytosed, the elementary body differentiates into the reticulate body, which is the intracellular form that replicates by binary fision.[2] Replication ceases approximately 72 hours following infection, after which the host cell lyses, releasing thousands of elementary bodies.[3] Classically, chlamydia is classified according to serotype: serotypes A-C are responsible for trachoma, D-K (and strains of B) for nongonococcal urethritis, and L1-L3 for lymphogranuloma venereum.[4] More recently, genotypic differences within a serotype have been explored.[5]

Recurrent infection with chlamydia in childhood causes repeated episodes of conjunctivitis, with adulthood sequelae that can lead to blindness. Acute conjunctivitis is typified by conjunctival lymphoid aggregations known as follicles, and papillary conjunctival inflammation. Adult sequelae include conjunctival scarring, entropion (in-turned eyelids), trichiasis (eyelashes scratching the ocular surface), corneal ulceration due to bacterial and fungal infection of the abraded cornea, and finally corneal scarring and blindness.[4] Humans are the definitive host.[6] Transmission is thought to occur from eye or nasal secretions by direct human contact, fomites, or from contact with eye-seeking flies, which breed in human feces.[4, 7-10]

Risk factors for trachoma have been well described. A higher prevalence of clinically active trachoma has consistently been observed in children than adults, though there is a higher prevalence of the sequelae of trachoma in adults.[11-14] Trachoma has been reported to occur more commonly in females.[11-12, 15-17] Poor facial hygiene[18-24] and poor access to water[11, 13-14, 21, 25-27] are associated with higher rates of trachoma, perhaps because of the presence of chlamydia in ocular and nasal secretions.[9] The presence of flies is associated with trachoma, and chlamydia has been isolated from flies, supporting the belief that flies transmit the disease.[8, 19-21, 23, 28-30] Trachoma is also associated with the absence of latrines,[11, 13, 20, 31] cattle ownership,[20, 23, 32] and lower altitudes,[16, 25, 33] perhaps because of increased number of flies in these situations. Trachoma is associated with crowded living conditions[14, 16, 21, 26] in rural areas.[11, 34]

Children may be a core group for the transmission of trachoma. In the case of trachoma, children are more likely to be infected,[35-38] have a longer duration of infection,[39] have higher infectious loads,[37-38, 40] and are likely to have more contacts per day with other members of the community, all of which make them likely to be a core group. Core groups were originally considered to be the individuals most likely to spread infection.[41] More formally, a core group has been defined as a subset of the population whose effective removal from transmission would bring about elimination of infection in the entire community.[42] In other words, infection persists in the non-core group due to contact with the core group. Eliminating infection in the core group prevents infection from persisting in the rest of the community.[43-44] There is emerging evidence that children are indeed a core group for trachoma (see preliminary studies).[45] If so, they would make a logical target population for treatment.

As one of the neglected tropical diseases (NTDs), trachoma has been designated as a priority eye disease by the WHO. Along with nongovernmental organizations, national health services, and Pfizer Inc., the WHO has instituted a program for the ***G***lobal ***E***limination of ***T***rachoma by the year ***2020*** (GET 2020). Part of this program is the recommendation of the "SAFE" strategy for reduction of blindness due to trachoma: ***S***urgery to repair vision-threatening trichiasis, ***A***ntibiotics to reduce the community pool of ocular chlamydia, ***F***acial hygiene to reduce chlamydial transmission, and ***E***nvironmental improvements to reduce chlamydial transmission from flies.[46-47]

Azithromycin is the antibiotic of choice for ocular chlamydia. In the past, trachoma was treated with a six-week course of daily tetracycline ointment, though adherence was typically poor. Compared to topical tetracycline, a single dose of oral azithromycin is equally or more effective, minimizing concerns about compliance.[48-51] Azithromycin is an azalide antibiotic closely related to the macrolides, with high efficacy, intracellular accumulation, and long tissue half-life.[52-54] Azithromycin has a favorable safety profile in children when given as a single dose for trachoma, or when given as long-term daily therapy in a variety of clinical settings.[55-56]

Trachoma treatment strategies must target the entire community. Ocular chlamydia infection can be asymptomatic,[37] and treated children are commonly re-infected.[57] In theory, mass antibiotic treatments treat asymptomatic carriers and prevent them from transmitting chlamydia to treated members of the community. In several studies, mass azithromycin distribution has been shown to decrease chlamydial infection and active trachoma.[40, 58-62]

Repeated mass antibiotic treatments are necessary in areas with severe trachoma. While a single mass azithromycin distribution has met with long-term success in some populations with low or moderate chlamydial infection,[40, 61] re-emergence of trachoma has been reported in hyper-endemic populations (Figure 1).[59-60, 62-63] Repeated azithromycin distributions may therefore be necessary in areas with a heavy burden of trachoma. Preliminary studies of repeated mass treatments are promising. Three rounds of annual azithromycin treatment in children 1-10 years of age nearly eliminated chlamydial infection in a moderately-affected region in Nepal.[64] Two rounds of mass azithromycin treatment, 18 months apart, reduced ocular chlamydial infection after 3.5 years of follow-up in a hyper-endemic community in Tanzania.[65] Repeated biannual mass treatments were superior to annual mass treatments in reducing ocular chlamydia in a two-year study in Ethiopia.[66] Models have suggested biannual or annual treatments may be sufficient to eliminate disease, depending on the prevalence of trachoma.[63, 67] Consistent with this, the WHO currently recommends three annual mass antibiotic distributions before reassessment for further treatment.[68]

While repeated mass antibiotic distributions are currently being implemented as part of the SAFE strategy by many trachoma programs, it is unclear when programs should stop these distributions. In areas with low levels of trachoma, it is likely that even one mass azithromycin treatment is sufficient to prevent the re-emergence of ocular chlamydia.[38, 61] In highly affected areas, ocular chlamydial infection clearly returns after a single mass azithromycin treatment (Figure 1).[59, 62, 69] In these heavily affected areas, even three annual mass antibiotic treatments is not enough to prevent the return of ocular chlamydia to a community once treatments are stopped (see preliminary studies).[70] Though there is a lack of evidence, the WHO recommends stopping annual treatment once clinical activity in children falls below 5%.[71]

Figure 1
Re-emergence of chlamydial infection after mass treatment in Ethiopia

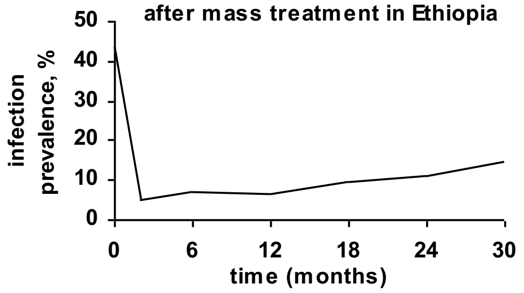

Once ocular chlamydia is reduced to a low level, targeted treatments may be useful to control or further reduce disease. In communities with low levels of trachoma, the WHO has advocated treating only those families in which at least one member has clinically active disease.[72] This recommendation may be based on research showing that ocular chlamydia clusters at the village, compound, household, and bedroom level, suggesting the importance of intra-familial transmission of disease.[12, 73, 74, 75, 76, 77] A clinical trial in Nepal found promise for this strategy (see preliminary studies), as did an observational study in Australia.[58], [78] Other studies have not; mass azithromycin treatments targeted to the households of children with clinically active trachoma did not prevent the

return of chlamydial infection after two annual rounds of treatment in Vietnam, and was less effective at reducing clinical activity compared to mass treatment in Mali.[79, 80] Alternatively, because children may form a core group for chlamydial infection, targeted treatment to children independent of trachoma status may be effective. In support of this theory, targeted treatment to all children was equivalent to mass azithromycin treatment in reducing clinically active trachoma in the Malian study.[80] Of note, no trials have as of yet assessed a targeting strategy after repeated mass antibiotic treatments, either as as a way to maintain low levels of infection, or as a way to further reduce ocular chlamdyia infection.

Nucleic acid amplification tests (NAATs) are the gold standard for detecting chlamydial infection.[81] Nucleic acid amplification tests (NAATs) have been performed by direct hybridization,[82] ligase chain reaction (LCR)[83] and polymerase chain reaction (PCR).[84] The most commonly used research method currently is PCR. Amplification targets for chlamydia include the cryptic plasmid, chromosomal genes (omp1 and omp2), and rRNA.[85] Quantitative PCR can be performed to assess the number of copies of the DNA target, and hence the infectious load, in a sample.[86] PCR is useful since it can detect non-viable, non-cultivatable pathogens, leading to a sensitivity greater than tissue culture;[87] disadvantages include cost and the risk of false positives from cross-contamination.[88] The commercially-available AMPLICOR (Roche Diagnostics, Branchburg, NJ) assay targets DNA within the cryptic plasmid, and is widely used in trachoma research.[38, 45, 61, 69, 89] The rRNA-based APTIMA assay (Gen-Probe, San Diego, CA) targets the 16S rRNA of *C. trachomatis*.[90] The rRNA-based assay has recently been shown to be more sensitive than DNA-based tests for the detection of sexually-transmitted and ocular chlamydia (see preliminary studies)[91-92].

Most trachoma programs do not believe they have the resources to use NAATs for detecting ocular chlamydia, and instead use clinical activity on conjunctival examination as a proxy for infection. Although many grading systems have been developed for trachoma,[93-100] only the WHO simplified system is widely used by trachoma programs and researchers to assess for clinical activity.[100] This system consists of the presence or absence of 5 signs (Table 4).[100] In general, individuals with follicular trachoma and/or intense trachoma (TF and/or TI) are considered to have clinically active trachoma, though some consider clinically active trachoma to be just TF. This simplified system demonstrated acceptable non-specialist inter-observer reliability,[101] and the WHO currently recommends basing treatment decisions on the prevalence of clinical activity using this system.[71]

A number of different studies have noted that the clinical diagnosis of trachoma as determined by the WHO grading system does not correlate well with laboratory testing for the presence of ocular chlamydial infection.[102] Some individuals with active clinical disease show no evidence of infection by NAAT,[103-107] whereas other persons without clinical disease do test positive for infection.[104, 106-108] Agreement between clinical exam and PCR is low.[107] Before treatment, 25-78% of clinically active individuals have no evidence of infection by NAAT (false positives), and 12-76% of infected individuals had no signs of clinical activity (false negatives).[38, 58, 73, 109-110]

The diagnostic properties of the clinical examination may improve after mass azithromycin treatments. As the prevalence of infection decreases after mass azithromycin treatments, using clinical activity as a measure of infection results in more false positives (88-94%), and a similar amount of false negatives (16-50%).[38, 58, 109] However, after mass antibiotic treatments, the proportion of clinically normal individuals without infection (i.e., negative predictive value) increases, from 58% to 97% in an area of hyperendemic trachoma, suggesting that the chance of missing an infected individual using the clinical exam is low.[109] Therefore, after mass antibiotic treatments, although using the clinical examination as a diagnostic test to direct treatment decisions

will result in over-treatment of uninfected individuals (false positives), the relative number of infected persons who go untreated may still be quite low, given a sufficient negative predictive value.

The presence of infection in an individual is not independent of infection in other members of a community, and analyses must take this into account. While some trachoma studies have been randomized at the community level, results have usually been analyzed at the level of the individual,[32, 36] which may falsely narrow confidence intervals and inflate statistical significance.[111] One way to avoid these statistical issues is to randomize treatment strategies to the community (e.g. neighborhood, village, school district), and analyze the data at the level of randomization.[112]

Economic analysis are important aspects of clinical trials for trachoma, especially given the global burden of trachoma blindness.[113-118] Economic evaluations allow decision makers to choose interventions that maximize health benefits for each unit of cost. Numerous studies have explored an appropriate methodology for cost-effectiveness analysis for trachoma programs.[119-121] Switching from topical tetracycline to oral azithromycin was found to cost £4-20 per cured infection.[48] Surgical repair of trichiasis has been found to be cost-effective.[122-123] Mass azithromycin treatment was found to be more cost-effective than targeted azithromycin treatment in an area with hypoendemic trachoma (see preliminary studies).[124] In an analysis of different world regions, mass azithromycin treatment was estimated to cost $23,000 per Disability Adjusted Life Year (DALY) averted in the region of Africa that includes Ethiopia.[123, 125] DALYs combine morbidity and mortality into a single metric; the cost per DALY quantifies the amount of money needed to prevent loss of life or quality of life. DALYs were developed by the WHO for cost-effectiveness analysis of global health interventions.[126] Note that the cost-effectiveness of trachoma interventions is especially important in areas with a low prevalence of chlamydia infection, such as after mass antibiotic treatments, since mass treatments in this setting may spend resources unnecessarily. The cost-effectiveness of targeted treatment strategies in low-prevalence areas is therefore of interest, since it could guide treatment decisions.

Significance. The current rationale of the trachoma control program is to reduce chlamydial infection to a low level with antibiotics and then prevent it from coming back with better hygiene and fly control. While antibiotics can reduce the prevalence, it is not clear whether repeat mass treatments can eliminate infection or not. The common wisdom is that they cannot. There is a large difference between eliminating infection and just controlling it by reducing it to a low level. With elimination, infection will not return even after treatment is discontinued. If a low level of infection persists, then continued intervention is necessary. Continued long term intervention with antibiotics brings up issues of drug resistance and sustainability that have yet to be addressed.

The development of a proven long term rationale for mass azithromycin treatment is *essential* if the control program is to reach its goal of eliminating blinding trachoma from even the most hyper-endemic areas by the year 2020. Much is riding on the success of the trachoma elimination program—not only is trachoma one of the leading causes of blindness worldwide, but the strategy for elimination may have implications for other diseases. A bacterial disease has never been eliminated by mass antibiotic distributions. If ocular chlamydial infection can be eliminated by repeat antibiotic treatment, and if it can be done by targeting only a core group of the population, then this may provide a valuable strategy against a variety of bacterial diseases.

3. Organization and Policies

The Carter Center and the Proctor Foundation will jointly implement field research. The Carter Center will be responsible for organizing field operations and for liaising with all parties within Ethiopia, including the Ministry of Health, for the implementation of this research. The Proctor Foundation will be responsible for providing the necessary technical assistance and

coordinating with The Carter Center, both in the field and at headquarters. The Carter Center and Proctor will abide by the Memorandum of Understanding (MOU), which includes mutually agreeable language specifying management roles and responsibilities, reporting, principal and co-investigators, and attribution of data and research. It was drafted and signed by the organization representatives from both collaborating parties.

3.1 Collaborating Institutions

Francis I. Proctor Foundation

The Proctor Foundation is an organized research unit at the University of California, San Francisco. The Foundation has a 56 year history of research in ocular infectious and inflammatory diseases and runs one of the leading corneal fellowship training programs in the United States. Proctor Foundation Faculty has been involved in prevention of blindness research in developing countries since the foundation's inception. The impetus for establishing the foundation in 1947 was to eradicate trachoma in the American Southwest and in other parts of the world.

From this initial vision we have expanded our research efforts to include the other major causes of blindness worldwide, with a continuing emphasis on infectious and inflammatory eye diseases. Dr. Thomas Lietman, Principal Investigator of the study at Proctor Foundation, will be assisted by numerous co-investigators, two study coordinators, a microbiologist, a data management specialist, laboratory PCR processing staff, and a biostatistician at UCSF.

Our experience working in Ethiopia has been very successful to date – with both our TEF (Trachoma Elimination and Follow-up) study in the Gurage region, and TANA (Trachoma Amelioration in Northern Amhara) in the Amhara region. We have partnered with ORBIS International and The Carter Center, respectively, to conduct/complete these large scale projects.

The Carter Center – Ethiopia

In October 2000, The Carter Center, with funding from Lions-Carter Center SightFirst Initiative, accepted the Ethiopian government's invitation to work on controlling trachoma in the Amhara region. When the program began in Amhara in 2000, trachoma was highly endemic among children less than 10 years old and adult women. Studies reported that approximately 88 percent of all children aged 1-10 years had active trachoma and an estimated 20 percent of adult women less than 40 years old were infected.

This baseline study further encouraged the program to conduct a needs assessment, which included interviews with schoolteachers. Studies revealed that most schoolteachers had minimal knowledge of the disease or its prevention. Teachers expressed interest in learning more and requested health education materials and structured guides for teaching trachoma prevention in the classroom.

The invaluable feedback led to discussions between The Carter Center and the Amhara Regional Health and Education bureaus. A working group developed a trachoma prevention curriculum and corresponding health education materials to cover all components of the SAFE strategy with an emphasis on "facial cleanliness" and "environmental sanitation." Each lesson encouraged students to identify other problems related to hygiene in their own communities and come up with their own solutions.

In addition to promoting school health education, the program continues ongoing health education promoting personal and environmental hygiene within the community. These activities target especially women and children.

To improve environmental sanitation, the program is promoting household latrine construction and use. From 2003 to June 2004, more than 70,000 household latrines were built in the Amhara region.

The Carter Center also worked with partner nongovernmental organizations to advocate for donation of Zithromax® from Pfizer Inc. As a result, in 2003, the program started mass treatment with azithromycin in trachoma-endemic communities. In 2004, a total of 625,422 people at risk for trachoma received treatment with azithromycin.

The Carter Center, Ethiopia Country Representative is Mr. Teshome Gebre and the Technical Director of the Carter Center's Trachoma Control Program is Dr. Paul Emerson. The focus of the country program has been on trachoma control, and delivery of quality eye care and blindness prevention programs to the majority of the population that live in rural areas (72%), largely un-served by trained eye care professionals.

The Carter Center is guided by a fundamental commitment to human rights and the alleviation of human suffering; it seeks to prevent and resolve conflicts, enhance freedom and democracy and improve health. The trachoma control program does not just fight disease, it fights the conditions that perpetuate disease: poverty, poor sanitation, lack of knowledge, and hopelessness. Together, the Carter Center and its program partners are working to build a brighter future for those at risk for this devastating disease.

Partnering with the Carter Center has yielded very positive results. The organization has an established infrastructure for large scale health promotion and improvement programs, as well as a strong working relationship with the Ethiopian Ministry of Health – both of which have contributed to the success of our field research.

Bahar Dar Regional Laboratory – Ethiopia

The Regional Laboratory in Bahar Dar was established to be a center of excellence for the development of laboratory systems in the region. Construction of this CDC and USAID supported laboratory was completed in 2011.

3.2 Duties & Responsibilities of Staff

3.2.1 Principal Investigator

- Develop study design, specific aims and outcome measures, with help of biostatistician and study coordinators, partners.
- Obtain grant funding and with help of partners, develop grant budget
- Ensure that staff follow through on protocol and properly execute all areas of research
- Ensure that all ethical approval is maintained
- Write or add major contributions to all study-related publications
- Ensure proper masking procedure for staff involved in the study
- Supervise training certification for all trachoma examiners

3.2.2 Study Coordinators (Proctor Foundation)

- Ensure the execution of the study per protocol
- Coordinate with the collaborating center, The Carter Center, Ethiopia (TCCE), particularly TCCE Study Coordinator in execution of the study
- Manage correspondence between all collaborating organizations and parties
- Maintain all ethical clearances for the study, including IRB renewals, ESTC and BUA and DSMC-related approvals
- In collaboration with TCCE Study Coordinator, prepare all forms and documents necessary for fieldwork (randomized registration forms, exam sheets, fieldwork documents directing team, etc)

- Train both Proctor and Ethiopian health worker team for each collection/treatment visit; direct team (with help of TCCE Study Coordinator) while in Ethiopia
- Arrange logistics and itineraries for traveling team members in Ethiopia
- Purchase, maintain, and organize transport of all necessary study supplies to/within Ethiopia
- Coordinate and oversee shipping of collected study samples per protocol and IATA regulations
- Maintain communication and partnership with Principal Investigator regarding all study activities and plans

3.2.3 Study Coordinator (The Carter Center, Ethiopia)

- At baseline, obtain permission from zonal health leaders to continue each state team in the TIRET study. Meet with and obtain permission/consent from all kebele and sub-kebele leaders.
- Supervise and train collection team (with emphasis on any new members) throughout each collection, especially after departure of Proctor study coordinators
- Train and supervise all Zithromax distribution teams
- Train and supervise all census taking teams
- Maintain all fieldwork documents and records
- Complete all collection and treatment reports
- Work with Data Coordinator to maintain treatment and census database
- Oversee shipment of study samples after departure of Proctor team
- Assist Proctor Coordinators in coordinating logistics in Ethiopia

3.2.4 Co-Investigators (US & Ethiopia)

- Assume responsibility for the study in the absence of the Principal Investigator
- Supervise ophthalmic assistants, ophthalmic nurses, local health agents and other Proctor field team members in the field to ensure conformity to study procedures
- Communicate with Study Coordinator and Principal Investigator to ensure the execution of the study as per the protocol

3.2.5 Microbiologist (ARSHO, Bahar Dar Regional Lab)

- Train and supervise laboratory technicians for all lab procedures concerning the study, including Polymerase Chain Reaction (PCR) procedures
- Perform all macrolide resistance testing
- Follow standard laboratory procedures for all tests
- Keep records of all tests performed
- Maintain quality control measures as per approved standards
- Make sure all equipment is calibrated and maintained
- Maintain stock of laboratory reagents and supplies

3.2.6 Data Coordinator (The Carter Center, Ethiopia)

- With aid of TCCE Study Coordinator, create and maintain database for all ***treatment related data*** for the study
- Monitor correct receipt of treatment/census data from the Study Coordinator using blue census books for each treatment or census visit
- Enter all data from treatment census books in clear database format for each visit and/or supervise data entry operators for any errors or omissions

- Analyze and provide data when requested by co-investigators or staff from Proctor Foundation
- Appropriately back-up all data
- Develop consistency checks in the data management

3.2.7 Database Specialist (Proctor Foundation)

- Create and maintain database for all ***collection and results-related data*** for the study
    - Monitor correct receipt of exam sheets after each collection visit
    - Enter all data from exam sheets in clear database format for each visit
    - Supervise double entry to ensure accurate recording (first entry by Database Specialist, second entry by temporary Proctor staff)
    - Develop consistency checks in the data management—verify any inconsistencies or questions regarding exam forms through communication with Study Coordinator
    - Analyze and provide data regarding collection and results for study staff when needed, such as for publications or DSMC meeting
    - Back-up all data appropriately
    - Follow-up on any missing data or lab results

Database Specialist (Bahar Dar Regional Lab)

- Organize all study samples for PCR processing in the Bahar Dar Regional laboratory
    - Monitor correct receipt of study samples (using corresponding exam sheets) after each monitoring visit
    - Prepare samples for PCR processing
    - Organize work flow of PCR processing for laboratory technicians, in consultation with Study Coordinator and Principal Investigator
    - Maintain all samples in storage freezers; create and maintain clear map of study samples for each freezer

3.2.8 Laboratory Technician (ARSHO, Bahar Dar Regional Lab)

- Pool and prepare all PCR samples, and process using COBAS Amplicor machine
- Perform regular maintenance checks of COBAS machine, under direction of microbiologist
- Report PCR results to Database Specialist
- Maintain cleanliness and maintenance of molecular biology laboratory and all equipment

3.2.9 Biostatistician/Data Analyst at Proctor

- Receive all data from the Data Coordinator/Study Coordinator TCCE and Database Specialists at Proctor Foundation, Bahar Dar Regional Laboratory, and ARSHO Laboratory.
- Review data for quality control purposes
- Ensure appropriate masking
- Prepare data analysis plan for annual Data & Safety Monitoring Committee (DSMC) meetings. Help analyze and prepare all presented data for DSMC meetings, DSMC reports, and all TIRET study publications

3.2.10 Treatment and Census Team: Local health extension workers from Ministry of Health, Ethiopia

- During census phase, travel to enrolled state teams and obtain patient information as required for all Census forms
- During treatment phase:
  - Complete training by TCCE Study Coordinator and officials from Local Zonal Health Desk regarding proper treatment protocols and procedures
  - Travel to enrolled state teams and administer study medication to the study subject per protocol
  - Counsel and motivate patients for follow-up and monitoring visits
  - Inform patients of available eye care facilities and procedures in local health centers and hospitals
  - Collect information on the nature of any Adverse Events experienced by study subjects, and report this back immediately to TCCE Investigators

3.2.11 Collection Team: Local health workers/nurses from Ethiopian Ministry of Health

- At each collection phase, complete on-going training and certification in clinical examination and collection procedures
- Prepare all study-related materials before travel to study sites
- In each state team, mobilize and identify all randomly selected participants
- Explain study purpose and procedure and obtain verbal consent for enrollment from each participant or participant guardian
- Under supervision of TCCE Study Coordinator and/or trained Proctor staff, perform clinical exam for each patient and collect all participant study samples according to protocol. Store and record all samples correctly for transport, organization, and processing
- Counsel and motivate patients for follow-up and monitoring visits
- Inform patients of available eye care facilities and procedures in local health centers and hospitals
- Collect information on the nature of any Adverse Events experienced by study subjects, and immediately report to TCCE Investigators

3.3 Policy Matters

3.3.1 Protocol Revisions during the Trial

Any changes to the protocol made during the course of the study will be incorporated in the revised protocol and the Manual of Operations and Procedures (MOP); any new forms will be incorporated in addendum. The protocol changes should be submitted and approved by the IRB of both the collaborating centers and by the Data and Safety Monitoring Committee (DSMC).

3.4 Presentations and Publications

All presentations and publications should include acknowledgement of the funding sources and give credit to the collaborating organizations and/or individuals involved.

3.4.1 Authorship Policy

Papers Presenting Primary Results

Acknowledgements will include grant source(s) and/or NIH grant(s), and the Data Safety and Monitoring Committee.

3.5 Data Safety and Monitoring Committee

A Data Safety and Monitoring Committee will be formed according to NIH guidelines. Members of the TANA DSMC have agreed to continue serving for the TIRET study. The Data Safety and Monitoring Committee will meet before the study commences and annually, and be observant for any severe and unexpected threats to the safety of the patients. They will have the authority to reduce any such threat, by discontinuing the enrollment of patients (were data to imply that these patients face unreasonable risk to antibiotics). As well as generally monitoring the safety and efficacy of treatment during the study, the DSMC will be empowered to enact formal stopping rules (see Stopping Rules, below).

3.5.1 Stopping (and Restarting) Rules

The WHO has recommended that trachoma programs revaluate their strategy after 3 years of implementation, but because we are monitoring intensely during this project, we have the opportunity to supervise treatment far more closely. In fact, we suspect from our previous studies that the treatments may be so effective that infection will be eliminated in some of the study communities long before 48 months. While there is an argument that continued mass treatments would prevent any reintroduced infection from taking hold, the balance between the risks and benefits of treatment will have changed. We will propose several types of stopping rules to the DSMC, all relatively conservative.

1) **Efficacy.** If an arm of Specific Aim 1 (biannual versus annual treatment) or Specific Aim 2 (treating children only versus treating everyone) is clearly superior in terms of both efficacy and resources, then the DSMC will have the opportunity to stop an arm. We will propose stopping rules to the DSMC at the pre-treatment meeting (using conservative, O'Brian-Fleming boundaries, with appropriate data and analysis provided by the biostatistician).

2) **Adverse effects.** More than 36 million doses of oral azithromycin have now been distributed in Ethiopia for trachoma (personal communication with Teshome Gebre, December 16, 2009), and reports of serious side effects are essentially non-existent. This may be due in part to minimal surveillance. It also may be due to the fact these are extremely rare with a single dose of azithromycin. In fact, where carefully monitored, there were actually fewer Gastro-intestinal (GI) side effects after taking azithromycin.[127] We will follow up on all reports of adverse events from local health centers/posts, as well as conduct a regular randomly selected survey to identify any possible post-treatment serious adverse effects. (Note that we are studying the resistance generated by antibiotic distributions in a separate grant, and all of this information will be made available to the DSMC). The DSMC will be given authority to discontinue treatments if the incidence of adverse events in treated arms becomes alarming.

3) **Stopping rule for individual state teams/communities.** If we are relatively certain that there is no ocular chlamydia left in a community, then continued treatment may not be warranted. If we are unable to identify ocular chlamydia in the sampled individuals of a state team on 2 consecutive visits, then an elimination endpoint is reached.

4. Patient Flow

4.1 Eligibility Requirements

As part of The Carter Center/ITI trachoma control program, about 1,200,000 of the population in the Amhara area will receive azithromycin. For this study, those sub-kebeles (Government-defined groups of approximately 5 sentinel development teams) that are within 4 kilometers of the center of the town of Gundeweyn as determined by Global Positioning System (GPS) readings will be excluded. This is because state teams falling within this diameter are semi-urban areas which are thought to have a lower prevalence of trachoma.

All age groups and sexes are eligible to receive azithromycin as per each study arm outlined above, except those contraindicated by Federal Ministry of Health, which currently are:

- Those self-reported as pregnant
- Children under six months old
- Those known to be allergic to azithromycin or macrolides such as erythromycin

These three exceptions will be treated with topical tetracycline eye ointment. Women uncertain of their pregnancy status may be offered an immediate-result pregnancy test if deemed appropriate by ethics committees.

4.2 Randomization

All 111 state teams from Arms A and B of the previous TANA study in Goncha Seso Enesie woreda will be eligible for randomization into the study. 12 state teams will be assigned to each of the six study arms:

- Arm J: Monitor after Annual Treatment ended
- Arm K: Monitor after Biannual Treatment ended
- Arm L: Annual Treatment
- Arm M: Biannual Treatment
- Arm N: Treatment for clinically active cases
- Arm O: Treatment for all children aged 0 – 5

Three to five development teams exist within each of the 72 enrolled sub-kebele. One development team from each sub-kebele will be randomly selected to undergo monitoring at each of the study time points (by clinical examination and collection of samples for DNA amplification). In each development team and at each collection, sixty individuals from each of two age groups will be selected at random from the census for monitoring:

- 60 persons age 0 to ≤ 9. Referred to as “children” for study purposes.
- 60 persons age ≥10. Referred to as “adults” for study purposes.

The list of these 120 individuals will be randomly re-selected at each collection time point, as the study works to monitor prevalence at the community versus the individual level.

Please note that this is a change from the age groups in the Research Design Section of the Grant (1-5 year olds, etc., page 49 of Grant Text). Since Specific Aim 2 involves treatment of children aged 0 to ≤ 9 years old, monitoring of a random sample of individuals within and outside this age group would provide more valuable information for this study.

4.3 Scheduling Visits

The time schedule for examination and treatment is shown below in Table 3.

Table 3: Examination and Treatment Schedule

	J	K	L	M	N	O
	**Stop-Annual**	**Stop-Biannual**	**Continue-Annual**	**Continue-Biannual**	**Target-Age**	**Target-Exam**
**Month 0**	Swab	Swab	Swab & Treat	Swab & Treat	Swab & Targeted Tx	Swab & Targeted Tx
**Month 6**				Swab & Treat		
**Month 12**	Swab	Swab	Swab & Treat	Swab & Treat	Swab & Targeted Tx	Swab & Targeted Tx
**Month 18**				Swab & Treat		
**Month 24**	Swab	Swab	Swab & Treat	Swab & Treat	Swab & Targeted Tx	Swab & Targeted Tx
**Month 30**				Swab & Treat		
**Month 36**	Swab	Swab	Bilateral Swab	Bilateral Swab	Swab	Swab

4.3.1 Baseline Visit

At baseline, before treatment, all households in the sampled development teams will be enumerated, and censuses of household members who are residents of the development team will be recorded on the Census/Registration form. Monitoring during this visit will be performed on a random sample of 60 people ages 0 to ≤ 9 years and 60 people age 10 and older. Monitoring will consist of clinical examination of the right upper tarsal conjunctiva using the WHO simplified trachoma grading, followed by swabbing of the conjuctiva. Samples will be tested with DNA amplification for the presence of C. trachomatis infection. Upon completion of examination and swabbing, antibiotic treatment will be distributed to the entire population of Arms L and M, to the clinically active cases and their households in Arm N, and to all children aged 0 – 5 in Arm O.

4.3.2 Treatment

After the baseline census and monitoring/collection is completed, treatment will be administered to all appropriate study development teams according to protocol. A treatment team, separate from the baseline enumeration and examination teams, will administer the antibiotic. The treatment team will consists of two local health extension workers (regional health staff) from each Kebele involved in the study. Each treatment cycle, this team participates in a training regarding antibiotic treatment and recording techniques. The training will be taught by Carter Center staff and members of the Zonal Health Desk, similar to the work flow of the TANA study.

During the baseline treatment phase for Arms L and M, Health Extension Workers will seek out each person on the census, administer the medication, and record whether or not each person has been treated. The treatment team will document the individual reasons for not being treated (e.g. death, temporary

absence, permanent migration, refusal for treatment, etc.). The antibiotic dosages distributed will also be documented.

In Arm N, antibiotics will be distributed only to those with clinically active trachoma and their households. Therefore, treatment will occur after the examination phase by the trained collection team, with team members going house to house as necessary to deliver antibiotics.

Treatment in Arm O will also occur after the examination phase, with team members seeking out all children aged 0 – 5 on the census. Medication will be administered and dosages documented. As with Arms L and M, the treatment team will document individual reasons for not being treated (e.g. death, temporary absence, permanent migration, refusal for treatment, etc.).

4.3.3 Follow-up Visits

Follow-up visits for all study arms are outlined in Table 3. At each monitoring visit, a random sample of 60 children age 0 to ≤ 9 years, and 60 adults, age 10 years and older in the appropriate study arms will be examined clinically. Swabs will be taken from these participants for DNA amplification in order to assess the presence of chlamydial infection.

4.4 Adherence to Treatment

Adherence to azithromycin treatment will be essentially 100% of those treated since administration of the single dose of antibiotic is directly observed by The Carter Center treatment distribution team.

4.5 Adverse Outcomes and Patient Death

4.5.1 Adverse Outcomes

In the event of an adverse outcome, the patient will no longer be enrolled in the study and an alternative treatment for trachoma (e.g. tetracycline ointment) will be administered if the patient needs to continue treatment.

If a patient experiences a serious adverse outcome, they will be advised to alert the chairman of the development team, who will then inform the health care representative. This person will in turn inform The Carter Center site manager, who will contact the Co-Investigator in Addis Ababa. In addition, periodic reports on adverse events will be made to the Data Safety and Monitoring Committee.

All adverse events will be recorded on the census for each individual who experiences them, and they will be monitored as usual until the study is completed. If, for any reason, they will need further eye care, they will be referred to the nearest health center for examination and treatment, and the most appropriate action will be taken to provide immediate care.

4.5.2 Patient Death

The infant mortality rate is quite high in this area of Ethiopia. All deaths since the first census will be carefully recorded during at second census before the 12 month visit. Since the major causes of infant mortality in the area are diarrhea, respiratory infections, and malaria, it is possible that receiving two to four Azithromycin treatments may actually have a positive effect. All death records (if available) will be maintained by the Data Coordinator at The Carter Center Ethiopia. The incidence of mortality for each study arm will be made available to the DSMC by the biostatistician.

5. Examination and Procedures

During registration and census-taking, each of the individuals from sampled state teams who are to be monitored for the study will be assigned a random identification number for laboratory and database anonymity. Examination of each individual will be performed as per the protocol outlined below:

5.1 Registering Participants for Examination

Prior to clinical examination the Registration Assistant will identify the participant, verify his/her age, and assign the next random identification number from the randomization list.

5.2 Gloving of the Examiner's Hands

Any hand that will touch a participant's face or eyelids must be gloved for the examination. The examiner will put latex gloves on both of their hands prior to touching the participants' eyelid, and a new pair of gloves will be used for each participant. Purell® Instant Hand Sanitizer will be available for hand sanitization.

5.3 Examination Positions

The examining position to be used in the field for young children will be the classic pediatric ophthalmic examination technique. With the aid of a helper seated directly opposite to the examiner, the child will be positioned with his/her head between the examiner's knees, with the child's face looking upwards toward the examiner. The legs of the child will be straddled across the helper and the arms held gently across the child's chest. Care should be taken to keep the child's eyes above the level of the examiner's knees, in order to properly take the conjunctival swab.

For examining adults and older children, the participant should stand or sit facing the seated examiner, such that the participant's eyes are at the examiner's eye-level.

5.4 Everting the Upper Eyelid

For all participants, only the right upper eyelid will be examined for this study. The only exception to this is if the right eye is difficult to examine due to eye disease or injury, in which case the left eye will be examined.

In order to avoid passing contamination from the child into the eye, once the examiner dons a new pair of gloves, their gloved hands should not be used to position the child. The examiner uses their fingertips to grasp the central portion of the participant's upper lid eyelashes. The upper lid is then everted, using a finger of the examiner's other hand (or the end of a sterile swab) as a fulcrum, positioned superior to the tarsal plate. The everted lid is held in place by the examiner's non-dominant hand holding the eyelashes against the orbital rim, thus keeping the examiner's dominant hand free for swabbing the participant's tarsal conjunctiva later.

5.5 Examining the Conjunctiva & Trachoma Grading

The clinical examination will be performed by trained ophthalmic nurses/technicians, public health specialists or ophthalmologists. The examiner uses the 2.5X magnifying ocular loupes (Wilson Ophthalmics) to assess the tarsal conjunctiva of the everted upper right eyelid. The examiner will grade the conjunctiva according to the World Health Organization Simplified Trachoma Grading Scale, as shown in Table 4 below. If necessary, a hand-held torch light will be used by the examiner for illumination of the conjunctiva. The examiner tells the Trachoma Grade to the tuber, who then records the grade onto the Examination form.

Inter- and intra-examiner reliability will be performed, but not as a part of this study, as the clinical examination is not being used as an outcome.

Additional Conjunctival Exam and Swabbing. Some study participants will receive multiple conjunctival swabbings, which may include the upper left conjunctiva. In study arms J and K, we will randomly select 200 children for an additional conjunctival swab of the upper left conjunctiva in the same method described above. Multiple conjunctival swabs from the same participant will be compared with each other to monitor the swabbing techniques of each nurse. No participant will be swabbed more than three times per visit. Multiple conjunctival swabbings does not increase risk to participants.

Table 4: WHO Simplified Trachoma Grading Scale

Definitions of the WHO Simplified Trachoma Grading Scale:

TF (Trachomatous Inflammation – Follicular): the presence of five or more follicles in the upper tarsal conjunctiva.

TI (Trachomatous Inflammation – Intense): pronounced inflammatory thickening of the upper tarsal conjunctiva that obscures more than half of the normal deep tarsal vessels.

TS (Trachomatous Scarring): the presence of scarring in the tarsal conjunctiva.

TT (Trachomatous Trichiasis): at least one eyelash rubs on the eyeball.

CO (Corneal Opacity): easily visible corneal opacity over the pupil.

Clinically active trachoma: *defined as either **TF** or **TI**. If WHO guidelines recommend that TF alone is the most appropriate sign to follow, then we will easily be able to report this.*

Chlamydial infection (infectious trachoma): *defined as positive PCR test for C. trachomatis.*

5.5.1 WHO Simplified Trachoma Grading Card

The WHO simplified trachoma grading card will be used as the reference standard for grading trachoma in the field. The card shows color photograph standards of each trachoma grade, clear descriptions of each grading category, and the standard abbreviations for the each trachoma grade.

5.6 Field Specimen Collection Quality Control Measures

The diagram shows the stations for examination and swabbing of patients, designed for the most efficient patient flow and to minimize errors or confusion between team members. This diagram shows the Station Configuration for a four-person team.

Quality assurance for conjunctival swab collection will include taking duplicate swabs on a randomly chosen 5% of individuals.

5.7 Examination Station and Diagram

Figure 2 illustrates a diagram for examining and swabbing patients that has been designed for efficient examination procedures and to minimize and errors or confusion between team members. This is depicted by the Station Configuration. It shows the station set-up of a four-person team.

Figure 2: Examination Station Configuration

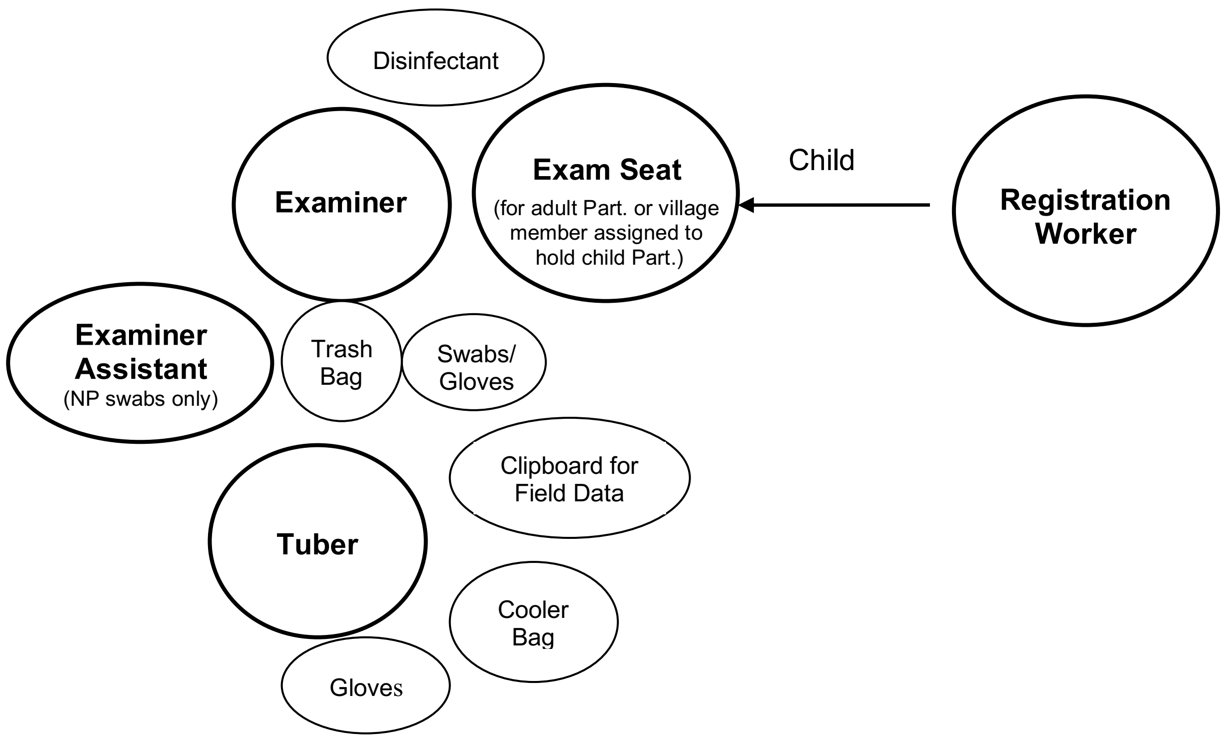

5.8. School Trachoma Examination Study.
Currently screening for trachoma is done by choosing a random sample of children aged 0-9 years from a district, and estimating prevalence of trachoma by examining these children. This should provide an accurate estimate of trachoma prevalence in the community. However, this methodology is time-consuming. Screening programs for other neglected tropical diseases have used schools as a sentinel site for screening. In this sub-study, we propose examining children from schools in our study area, and comparing the prevalence of trachoma from this school population with that from the village-based monitoring we are doing for the clinical trial.

6. Microbiology Laboratory Procedures for PCR

6.1 Specimen Collection for Microbiological Tests (Principal)

Samples will be collected with reference to age, gender, household, and development team, but participant names will not be included in laboratory records. Samples will thus not be associated with the individual's name, but with a 6-digit random identification number, masking laboratory personnel and preventing identification of the individuals infected. Lab results will not be available for weeks if not months, typically after the average duration of an ocular chlamydial infection. Thus all individuals will be treated according to their study arm, whether or not their lab tests reveal evidence of chlamydial infection.

6.2 Materials for PCR

6.2.1 Swabs

Specimens will be collected using sterile, individually-wrapped Dacron polyester-tipped swabs with a plastic shaft (manufactured by Fisherbrand®), as recommended by the COBAS AMPLICOR™ CT/NG protocol.

6.2.2 Sample Tubes

All field samples for DNA testing will be collected into sterile 2.0ml microcentrifuge tubes, manufactured by RPI®.

6.2.3 Cooler Bags with Frozen Ice Packs

Insulated cooler bags (California Innovations™) will be used to carry samples to and from the field. In addition, frozen gel ice packs designed to thaw slowly will be used to maintain the temperature in the cooler bags during transport.

6.2.4 -20°C Freezer

A standard -20°C freezer located at the Motta Health Center will be used strictly for the storage and freezing of ice packs and samples. This freezer is kept in a locked room on the grounds of the Health Center, which is under 24-hour security guard supervision.

6.2.5 Latex Gloves

Fisherbrand® latex examination gloves will be used to perform conjunctival examinations for trachoma. Each glove will be used once and never shared between participants. Used gloves will be collected in a trash bag and incinerated at the local Health Center incineration facility.

6.3 Training of Examiners

6.3.1 Pre-Monitoring Visit Training

All specimen collectors and graders on the Proctor team new to the study will complete a three-hour training at the Proctor Foundation before departing for Ethiopia. The training will focus on 1) study background and rationale, 2) how to

perform the clinical exam and 3) how to perform all collection procedures and protocols in order to standardize procedures among graders and tubers. The training will be led by the Proctor Study Coordinators, with input from the Database Manager and laboratory technician on proper collection and data recording techniques. The Principal Investigator or Co-investigator will offer in-depth training on performing the clinical exam.

6.3.2 In-Country Training

Before the start of the TANA study, all local health workers assigned to complete study fieldwork underwent a series of rigorous trainings and certification in clinical grading and swab collection. At the start of each successive collection phase, examination team members (both local health workers from the Ministry of Health and Proctor staff) will participate in a full day of training at a staging site in the town of Gundeweyn, Ethiopia. The Principal Investigator and Study Coordinators will offer on-going training and supervision to all specimen collectors and graders in the field throughout the TIRET study monitoring visits.

6.4 Specimen Collection

6.4.1 Protocol for swabbing of the conjunctiva

1. Upon taking a swab from the box, the tuber should announce what type of swab it is:
    - An unmarked “plain swab,” indicating no control or second swab collection necessary for this patient.
    - A "blue air swab" (negative field control), indicating examiner must collect a second swab for this patient. Swab sachet will be marked with blue color.
    - A “green air swab” (RNA field control), indicating examiner must collect a second swab for this patient. Swab sachet will be marked with green color.

    The swab sachet should be opened in a sterile manner, revealing only the tip of the swab shaft, with the swab head itself remaining sterile deep within the sachet.

2. When ready to swab, the examiner should slowly pull out the swab (if removed too quickly the Dacron swab tip can unravel). The gloved hand should be held no closer than 1 inch from the Dacron polyester swab tip during the entire swabbing procedure, in order to avoid contamination of the swab tip.

3. As the examiner holds the right eyelid in the everted position, they swab the upper tarsal conjunctiva of the child with a gloved hand, using a steady and firm swab. Ideally the swab should be placed flat, with its entire length parallel to the conjunctiva to give the greatest surface area of contact, and if necessary, this may require repositioning of the child’s head by the examiner. The swab should be drawn firmly in one direction over the conjunctiva with enough pressure to cause blanching of the conjunctival vessels. The swab should then be rotated 120 degrees along its axis and the newly revealed fresh region of Dacron on the swab tip should now be drawn firmly across the conjunctiva. The swab should be rotated another 120 degrees along its axis and the conjunctiva swabbed for a third time. This will ensure sufficient collection of conjunctival epithelial specimen for PCR analysis in the lab.

    Note: An aggressive twisting motion toward the cornea can push the conjunctiva upwards (towards eyebrow) which can lead to the swab falling onto cornea.

Thus a pure lateral motion or slight twisting toward the eyebrow is preferable, in order to ensure safety of the cornea at all times. Quick movements should be avoided (less control, increased contamination, risk of touching the cornea). Instead, a slow, steady motion should be carried out. Care should be taken to avoid swabbing the eyelashes. Traction on the lower lid by the non-swabbing hand (or by an assistant) can keep the lower lid lashes from inadvertently touching the swab. Possibilities: The non-dominant (non-swabbing) hand should be placed on the patient's forehead to give greater steadiness. This may also serve to alert the examiner to any movement by the patient (tactile instead of just visual). Also, this action will help steady the head of a child in case of movement.

4. RNA swabs will be collected in the 84-month study visit for Arms L and M. The examiner will repeat steps 2 and 3 with a second swab, taking care to hold the swab shaft outside of the score line (about 3 inches away from the swab tip).

6.4.2 Control Swabs

A negative field control for PCR ("blue air swab") will be taken on a randomly chosen 5% of monitored children to assess the frequency of contamination. For each negative field control, the examiner will pass a sterile Dacron swab within 1 inch of the child's conjunctiva. Control swabs are taken after the original swab but before changing gloves for the next patient.

During the 84-month study visit, another 5% of monitored children will be randomly chosen to receive a negative field control for RNA ("green air swab"), following the same procedure as listed for the PCR control.

6.4.3 Protocol for tubing and handling of samples

The tubing and handling protocol must be carefully followed in order to prevent contamination and ensure the safe transport of the samples to the Carter Center (Addis Ababa) and the Bahar Dar Regional Health Laboratory. The person in charge of labeling, tubing, arranging, and handling the samples needs to perform this task in the most orderly and attentive manner.

1. Both hands of the tuber should be gloved at all times. The tuber's gloves only need to be changed when any potential contamination of the gloves occurs. The tuber opens the capped, hinged lid of a microcentrifuge tube, which has been labeled with the participant's random identification number.

2. The swab is inserted by the examiner (using the still-gloved hand that swabbed the participant's conjunctiva) into the microcentrifuge tube held by the tuber. The swab shaft should only be inserted until the Dacron swab head is fully in the tube. The tuber should lower the cap onto the swab shaft held by the examiner, and the examiner should quickly break the swab shaft using a swift downward snapping wrist movement (this will be demonstrated in the field the first day).

3. The tuber should screw the cap of the microcentrifuge tube tightly and place it in the sample collection box, located in the cooler bag filled with frozen ice packs. The flap of the cooler bag should be closed between each patient. The cooler bag should be in as cool a place as possible in the field, in a shaded area out of the sun.

4. Upon returning from the field each day, the samples will be immediately taken to the Motta Health Center and stored in a commercial -20°C freezer, reserved solely for storage of specimens and ice packs. All samples will be in sample boxes, labeled with the state team and district names for easy future identification.

6.5 Transport and Storage of Samples

In accordance with the Roche COBAS AMPLICOR™ CT/NG protocol, swab samples taken in the field will be transported on ice in a closed, insulated container until arrival at the Bahar Dar Regional Laboratory, where they will be stored at -80°C for later analysis.

6.6 Methods

Laboratory testing is the current standard of care for the identification of *C.trachomatis* infections in the U.S. After collection, all samples will be filled with 1 ml of M4RT media at the Proctor laboratory, and tested for *C. trachomatis*.

6.6.1 Description of the Roche COBAS AMPLICOR™ CT/NG Assay

The conjunctival swabs will be tested for the presence of *C. trachomatis* using the Roche COBAS AMPLICOR™ CT/NG assay. This assay is a qualitative *in vitro* test for the detection of *C. trachomatis* plasmid DNA. The assay is based on four major processes; specimen preparation: PCR amplification of target DNA using CT specific complementary primers; hybridization of the amplified DNA to oligonucleotide probes specific to the target; and detection of the probe-bound amplified DNA by colorimetric determination.

6.6.2 Processing of Samples for PCR

Samples are handled as per COBAS AMPLICOR™ CT/NG sample processing protocol, with the following modifications:

1. Samples are boiled for 10 minutes at 100°C. Boiling of samples is an accepted treatment method to remove substances that may be inhibitory to the PCR amplification process.
2. Samples are pooled.

6.6.3 Procedure for Masking Samples

In order to mask the location origin, the control status, and the clinical exam grading of the conjunctival samples collected in the field, an identification number different from the random number will be assigned to each child. The PCR results will be recorded according to this laboratory identification number, thus masking the lab until all the samples have been processed. The Database Manager will then link the lab ID number to the random sample number to reveal the test results by state team.

6.6.4 Procedure for Pooling Samples

At baseline, all samples will be processed individually. At post-treatment visits, samples will be divided by state team and age group (0 to ≤ 9 years and 10 and older). Within a state team and an age group, samples will be randomly pooled 5 per tube as described below (note that if the number of samples in a group is not divisible by 5, then a single 4-pool, 3-pool, 2-pool, or unpooled tube may be necessary). If, in a state team, the proportion of pooled positives is greater than 50%, then samples will be re-pooled with 2 per tube, and the PCR repeated. Analysis of pooled results is described in Section 2, “Pooling of chlamydial tests”.

In order to pool the conjunctival samples in the lab, the microbiology lab staff will assign a new pool ID number for every sample, and samples will be stored at -80°C freezer until PCR testing (if not processed that day).

6.7 Quality Control

1) A *C. trachomatis*(+) control and a *C. trachomatis*(-) control is included in each test run of the COBAS AMPLICOR™ CT/NG.
2) To test the effect of sample processing, a known positive sample is processed and tested in each test run. (This control is helpful when testing large numbers of negative samples.)
3) An internal control intended to identify specimens that contain polymerase inhibitor is run routinely on each sample. The internal control helps identify false negative results.

6.8 Laboratory Results Reporting

All lab results will be kept in computer files as well as in hard-copy form by the Database Manager. The principal investigator and the DSMC will be updated regularly on the progress of the lab work throughout the course of the study.

7. Macrolide Resistance Testing

7.1 Specimen Collection for Macrolide Resistance Testing

Nasopharyngeal samples will be collected with reference to age, gender, household, and state team, but participant names will not be included in laboratory records. Samples will thus not be associated with the individual's name, but with a 6-digit random identification number, masking laboratory personnel and preventing identification of individuals.

7.2 Materials for NP Collection

7.2.1 Nasopharyngeal Swabs

Specimens will be collected using sterile, individually-wrapped pediatric calcium alginate swabs with a malleable metal swab shaft for patient comfort and safety (manufactured by Fisherbrand®).

7.2.2 Nasopharyngeal Sample Tubes

All field samples for DNA testing will be collected into sterile 2.0ml microcentrifuge tubes, manufactured by RPI®.

7.2.3 Cooler Bags with Frozen Ice Packs

Insulated cooler bags (California Innovations™) will be used to carry samples to and from the field. In addition, frozen gel ice packs designed to thaw slowly will be used to maintain the temperature in the cooler bags during transport.

7.2.4 -20°C Freezer

A standard -20°C freezer located at the Mota Health Center will be used strictly for the storage and freezing of ice packs and samples. This freezer is kept in a locked room on the grounds of the Health Center, which is under 24-hour security guard supervision.

7.2.5 Latex Gloves

Fisherbrand® latex examination gloves will be used to perform nasopharyngeal examinations. Each glove will be used once and never shared

between participants. Used gloves will be collected in a trash bag and incinerated at the local Health Center incineration facility.

7.3 Training of Examiners

The first day of fieldwork is a training day for all specimen collectors and graders on the Proctor and Carter Center Ethiopia teams. The Principal Investigator and Study Coordinators will offer on-going training and supervision to all specimen collectors and graders throughout the monitoring visit.

7.4 Nasopharyngeal Specimen Collection

7.4.1 Protocol for Nasopharyngeal Sample Collection

During collection visits that include nasopharyngeal sample collection, 15 swabs in the box will be marked to indicate participant selection for NP sample collection.

1) In this case, upon taking a DNA swab from the box, the tuber should announce what type of swab it is:
   - An unmarked "plain swab", indicating no control or second swab collection necessary for this patient.
   - A "blue air swab" (negative field control), indicating examiner must collect a second swab for this patient. Swab sachet will be marked with blue color.
   - An "NP swab", indicating examiner must also collect a nasopharyngeal swab for this patient. Swab sachet will be marked green.

2) If the swab is an "NP swab" marked green, a trained team member will immediately be called to assist the exam team. Standing behind the examiner, the assistant will select a pediatric calcium alginate swab (manufactured by Fisherbrand®), and open the swab sachet in a sterile manner (revealing only the tip of the swab shaft, with the swab head itself remaining sterile deep within the sachet). Immediately after completion of the conjunctival eye swab collection (and if necessary, control swab collection), the examiner will remove the swab from the sachet and place the tip down the participant's nasopharynx.

3) The examiner will quickly rotate the swab 120° three times back and forth, and then remove the swab from the nose.

4) The examiner will place the swab in a tube containing 1.0 mL of STGG (skim milk, tryptone, glucose, and glycerin) media. The assistant will cut the handle off using sterile scissors (cleaned with alcohol pads between participants). The tuber will close the cap of the STGG tube with the swab immersed.

7.5 Transport and Storage of Samples

The nasopharyngeal swab samples in STGG will be initially stored in the field at 4°C using an insulated storage bag filled with Fisher brand ice gel packs and then transferred to -20°C.

All Nasopharyngeal samples will be transported on ice in a closed, insulated container until arrival in Addis Ababa, where they will be stored at -80°C for later analysis.

7.6 Methods

The nasopharyngeal swab for pneumococcal resistance testing will be transported and processed using standard microbiological techniques. Isolates will be confirmed *S. pneumoniae* by growth on *Streptococcus*-selective media, alpha-hemolysis, susceptibility to

optochin and bile salt solubility. MIC values for azithromycin and trimethoprim/sulfamethoxazole will be determined using standardized broth dilution.

7.6.1 Procedure for Masking Samples

The microbiologist and all lab staff will be masked to the state-team of origin.

7.6.2 Quality Control

Control isolates will be used to confirm the reliability of antibiotic susceptibility testing.

7.6.3 Laboratory Results Reporting

All lab results will be kept in computer files as well as in hard-copy form by the Microbiologist.

8. Quantification of Ocular Chlamydia.

Identification of individuals with high chlamydial load is potentially important, as these individuals likely form the major reservoir for chlamydial re-infection, and therefore may be preventing trachoma elimination in communities.We propose to unpool all swab pools that are positive for chlamydia from each of the 72 communities at each of the 4 annual visits in TANA since 2010 and to test each specimen separately using the Abbott m2000 RealTi*m*e system. We will assess the variability of chlamydial DNA load by performing tests on duplicate swabs from the same study subject. We will compare the chlamydial DNA load in individuals with and without clinical conjunctival inflammation as graded at the study visits using the WHO simplified grading scale. We will also compare chlamydial load in the communities treated with annual mass azithromycin versus those treated with biannual mass azithromycin.

9. Clinical Photography

9.1 Photography Protocol

To measure accuracy of grading and inter-grader reliability, photographs will be taken of the upper right tarsal conjunctiva of a subset of children examined for conjunctival swabbing. Clinical photography will be performed before conjunctival swabbing. A handheld Nikon D70 digital camera with a macro lens (1:1) will be used. This causes no damage to the eye, is well tolerated by children, and is a standard clinical procedure at UCSF.

9.2 Grading of Clinical Photographs

A trained grader at the Proctor Foundation, UCSF, will grade the photograph of the conjunctiva according to the WHO simplified trachoma grading scale as either active (TF or TI) or inactive. The grader will be masked as to the treatment given.

9.3. Additional Photography

In study villages N and O, additional photographs will be taken with the iPhone of the upper right tarsal conjunctiva of all children (aged 6 months to five years) examined for conjunctival swabbing. Photography causes no damage to the eye and is well tolerated by children and is a standard clinical procedure at UCSF.

10. Blood Testing

During the 36-month study visit, all children who present for a conjunctival exam will also receive blood testing. For any child who is febrile, a rapid diagnostic test for malaria will be conducted. Trained examiners will collect a finger- or heelstick of capillary blood which will be used for a thick blood smear to assess for malaria parasitemia, hemoglobin testing (HemoCue, Ängelholm, Sweden), and dried blood spots for future PCR testing. Blood will be collected by a gloved health worker using aseptic technique. The finger- or heel-stick site will be disinfected using an alcohol

swab. Once the alcohol dries, the health worker will prick the finger/heel with a disposable lancet, and subsequently apply moderate pressure approximately 1 cm behind the site of the puncture to obtain sufficient drops of blood. The lancet will be safely disposed of in a biohazard waste container. Gloves will be changed between each patient.

After greeting the study participant, **the examiner will call out the following information to the recorder:**

- Participant's random number (read from random number sticker)
- Participant's Age and Gender

During the exam, the recorder writes all the data on the data sheet corresponding to the village location. Specifically, the recorder:

- Locates the sticker matching the number for the child and places it on the data sheet
- Records the age and gender of each child
- Writes all measurements announced by the Examiner

Inform the mother that you will prick her child's finger to obtain blood to test for malaria and anemia. Describe the finger prick procedure and reassure her. The blood specimen should be collected as described below to minimize the discomfort of the child and to ensure enough blood is collected.

It is important to handle all blood specimens with care because they may be infectious.
Wear gloves. New gloves must be worn for each child.
Clean spills. If there are any blood spills or splashes, you must clean it up immediately with the approved disinfectant (10% bleach or chlorhexidine solution) and wipe it up with absorbent material.
Disposal of sharps. All disposable lancets must be disposed of properly in the sharps container.
No food. Food or drink is not allowed at the blood collection station.

10.1. Finger Prick

Prepare the disposable lancet. Make sure to use a NEW disposable lancet for each child. **Do not** re-use lancets! Check that the lancing device is set on the appropriate setting for young children.

Position the child for the finger stick. Make sure that the child's right hand is warm and relaxed. Hold the child's thumb, middle, or ring finger on his/her right hand (from the top of the knuckle to the tip of the finger) between your left thumb and finger and disinfect with an individually packaged alcohol wipe in small outward circles.

After the alcohol dries, to stimulate blood flow towards the sampling point (puncture site), use your thumb to lightly press the child's thumb or finger from the top of the knuckle towards the tip. For the best blood flow and least pain, prick the side of the thumb-tip or fingertip, not the center. While applying light pressure towards the thumb-tip or fingertip, hold the lancing device in your right hand and prick the thumb or finger. If the finger prick is performed properly, one prick should be sufficient to collect the required amount of blood.

Allow the blood to ooze out. Wipe away the first 2 or 3 drops of blood with gauze. If necessary, re-apply light to moderate pressure towards the thumb--tip or fingertip (approximately 1 cm behind the site of the finger prick) until another drop of blood appears. ***Note:*** **Do not** squeeze too hard (avoid "milking") as it may dilute the blood with tissue plasma.

10.2. Thick smear and thin smear

One glass slide will be prepared to collect and thick and thin smear from each child. Two drops of blood will be placed on the labeled glass slide; one will be spread across half of the slide. The smears will be allowed to air dry, and then fixed in the field using 100% methanol for 30 seconds. Smears will be transported at room temperature each day to the Gundaweyn health center. Within 24 hours of collection, the smears will be stained with 3% Giemsa stain for 30-45 minutes,10and parasite density will be read using a light microscope by two experienced microscopists masked to community treatment assignment. Microscopists will record the number of parasites and the number of gametocytes observed per 200 white blood cells. Parasites and gametocytes will be considered present if either microscopist observes them, with sensitivity analyses being performed only counting presence if both observe. If necessary, any discrepancies will be reviewed by a third microscopist.

- Place a drop of blood in the center (1 cm from the edge of the slide) of a clean, dust-free, and grease-free slide.

- To make the thick blood smear, spread it evenly with a disposable wooden applicator or with another clean slide in a round shape with a diameter of 1cm. The blood smear should be about 1cm away from the edge of the slide.

- The correct thickness of a thick blood smear is one through which newsprint is barely visible (when the blood is still wet, not dry).

- Allow the blood smear to air dry flat. Do not heat the slides, this will damage the parasites. Be sure to protect the slide from dust and insects. Do not refrigerate slides, as this may cause the smears to detach from the slide during the staining procedure.

- When dry, place the thick blood smear into the slide box.

- After enough blood is collected, release the pressure immediately to allow recirculation of the blood. Repeat the last two steps until enough blood has been collected. Apply a piece of cotton to the finger prick site using slight pressure until the bleeding has stopped.

10.3. Hemoglobin
A portable spectrophotometer (HemoCue, Ängelholm, Sweden) will be used for hemoglobin testing. A cuvette will capture a drop of blood at the fingerprick site until it is completely full. The cuvette will be will be placed in the cuvette holder of the HemoCue device for an immediate hemoglobin reading and disposed of in an appropriate biohazards waste container. In all cases, the study participant will be identified by his/her study random identification number. Any children with fever, any level of malaria parasitemia, or severe anemia will be referred to a local health clinic for care.

- Remove a cuvette from the container. Reseal the container immediately. (The recorder can help the examiner with this.)

- When the blood drop is large enough, fill the microcuvette in one continuous process. **Do not refill!** If there is not enough blood to fill the microcuvette, you must start again with a new microcuvette. Wipe any excess blood from the sides of the microcuvette with clean gauze or a paper towel, but be careful not to touch the open end of the microcuvette.

- Look for any air bubbles in the filled microcuvette. If air bubbles are present, discard the microcuvette and obtain a new drop of blood using a new microcuvette. (Small bubbles around the edge of the microcuvette can be ignored.)

- Place the filled microcuvette in the cuvette holder. Gently slide the cuvette holder to the measuring position to be analyzed immediately. (This **must** be performed within 10 minutes after filling the microcuvette!)

- After 15 – 60 seconds, the hemoglobin value will be displayed. The Examiner should read the hemoglobin value aloud so the Recorder can write it on the examination form. The value will remain on display as long as the cuvette holder is in the measuring position. The analyzer will turn off automatically after 5 minutes.

- Carefully, dispose of the used microcuvette in the sharps container.

10.4. Dried blood spot

Four drops of blood will be placed onto FTA Elute cards (Whatman, Kent, UK) and labeled with the study participant's random identification number. Once blood spots are completely dry, they will be placed in a sealed individual storage bag and stored at room temperature in a locked cabinet in the study coordinators' office.

- Place 4 large drops of blood directly from the thumb or finger onto the large circle on the filter paper, making a square.

- Leave the filter paper to air dry for a few minutes, then place the sample into a small plastic bag along with a dessicant packet.

- Leave the bag open for a few minutes more, and when the blood is **completely** dry, roll down the top of the bag and close with a piece of masking tape.

- Store the filter paper samples (in the small plastic bag) in a larger Ziploc bag. Keep all filter paper samples in a safe, dry place at room temperature.

10.5. Materials for blood collection

Gloves (120 pairs)
Lancing device, if desired (1)
Disposable lancets (100)
Alcohol wipes (70)
Cotton balls (70)
Gauze
10% household bleach or 4% chlorhexidine solution to clean spills
Absorbent material for spills
Sharps container (1)

Filter paper (70)
Small plastic bags (70)
Desiccant packs (70)
Masking tape
Large Ziploc bags (handful)

HemoCue machine
Extra set of AA batteries (4)
Cuvettes (60)
Q-tips (handful)

Glass slides (70)
Cover slips (70)
Disposable wooden applicator sticks (70)
Slide box

Clipboard with exam forms

11. Anthropometry
Anthropometric measurements in children can be important indicators of morbidity and predictors of mortality. Trained health workers will take anthropometric measurements, including height, weight, and mid-upper arm circumference (MUAC) (according to WHO guidelines and recommendations) of children (aged 6 months to 5 years) from the 24 communities in study arms K (stop biannual) and M (continue biannual). These measurements will be recorded in March 2012 and 2014.
After greeting the study participant, the examiner will call out the following information to the recorder:

- Participant's random number (read from random number sticker)
- Participant's Age and Gender

During the exam, the recorder writes all the data on the data sheet corresponding to the village location. Specifically, the recorder:

- Locates the sticker matching the number for the child and places it on the data sheet
- Records the age and gender of each child
- Writes all measurements announced by the Examiner

Next, the examiner should observe the child for signs of kwashiorkor, marasmus, and pedal edema:

Kwashiorkor: look for edema (or swelling); thin, sparse, or discolored hair; and skin with discolored patches that may crack and peel.
Marasmus: look for severe wasting; the appearance of 'skin and bones;' and a face that looks like an old man's.
Pedal edema: look for swelling due to excess fluid in the foot. Press the child's foot with your thumb. If the foot is swollen and the indentation remains after you press it, the child has edema.

Once you've observed the child for these 3 signs of malnourishment, the child is then ready to have his/her height, weight, mid-upper arm circumference (or MUAC) measured.

11.1. Height
A lightweight measuring board (ShorrBoard) will be used to measure the participant's height to the nearest 0.1cm. Depending on a child's age and ability to stand, measure the child's length or height. A child's length is measured lying down (recumbent). Height is measured standing upright.

If a child is less than 2 years old, measure recumbent length. Circle **L** on the exam form to indicate that length was measured.

If a child is aged 2 years or older and able to stand, measure standing height. Circle **T** on the exam form to indicate that height was measured.

If the child has braids or hair ornaments that will interfere with length/height measurements, remove them if possible. Check that shoes and socks have also been removed. Whether measuring length or height, the mother is needed to help with measurement and to soothe and comfort the child. Explain to the mother the reasons for the measurement, and the steps in the procedure. Answer any questions she might have. Show her and tell her how she can help you. Explain that it is important to keep the child still and calm to obtain a good measurement.

Measure length
Cover the length board with a chuck for hygiene and for the baby's comfort.

Explain to the mother that she will need to place the baby on the length board and then help to hold the baby's head in place while you take the measurement. Show her where to stand when placing the baby down (i.e. opposite you, on the side of the length board away from the tape). Also show her where to place the baby's head (against the fixed headboard) so that she can move quickly and surely without distressing the baby.

When the mother understands your instructions and is ready to assist:
Ask her to lay the child on his back with his head against the fixed headboard, compressing the hair.

Quickly position the head so that an imaginary vertical line from the ear canal to the lower border of the eye socket is perpendicular to the board. (The child's eyes should be looking straight up.) Ask the mother to move behind the headboard and hold the head in this position.

Speed is important. Standing on the side of the length board where you can see the measuring tape and move the footboard:
Check that the child lies straight along the board and does not change position. Shoulders should touch the board, and the spine should not be arched. Ask the mother to inform you if the child arches the back or moves out of position.

Hold down the child's legs with the one hand and move the footboard with the other. Apply gentle pressure to the knees to straighten the legs as far as they can go without causing injury. ***Note:*** it is not possible to straighten the knees of newborns to the same degree as older children. Their knees are fragile and could be easily injured, so apply minimum pressure.

If a child is extremely agitated and both legs cannot be held in position, measure with one leg in position.

While holding the knees, pull the footboard against the child's feet. The soles of the feet should be flat against the footboard, toes pointing upwards. If the child bends the toes and prevents the footboard from touching the soles, scratch the soles slightly and slide in the footboard quickly when the child straightens the toes.

Upon reading the measurement, the examiner will clearly call out the number to the recorder.
Record the child's length in centimeters to the last completed 0.1 cm. (0.1 cm = 1 mm) Keeping the child in place, release the sliding footboard, and prepare to repeat the measurement. Re-position and measure the child three times.

Measure standing height
Ensure that the height board is on level ground. Check that shoes, socks and hair ornaments have been removed.

Working with the mother, and kneeling in order to be at the level of the child:
Help the child to stand on the baseboard with the weight of the child is evenly distributed on both feet. The heels of the feet are placed together with both heels touching the base of the vertical board. Place the feet pointed slightly outward at a 60 degree angle. The back of the head, shoulder blades, buttocks, calves, and heels should all touch the vertical board. Arms hang freely by the sides of the body with the palms facing the thighs. ***Note:*** Standing with all body parts touching the board may be difficult for some children, in which case, help the child to stand on the board with one or more contact points touching the board.

Ask the mother to hold the child's knees and ankles to help keep the legs straight and feet flat, with heels and calves touching the vertical board. Ask her to focus the child's attention, sooth the child as needed, and inform you if the child moves out of position.

Position the child's head so that a horizontal line from the ear canal to the lower border of the eye socket runs parallel to the baseboard. To keep the head in this position, hold the bridge between your thumb and forefinger over the child's chin.

Ask the child to inhale deeply and to stand fully erect without altering the position of the heels. If necessary, push gently on the tummy to help the child stand to full height.

Still keeping the head in position, use your other hand to pull down the headboard to rest firmly on top of the head and compress the hair.

Upon reading the measurement, the examiner will clearly call out the number to the recorder. Record the child's height in centimeters to the last completed 0.1 cm. (0.1 cm = 1 mm) Keeping the child in place, release the sliding headboard, and prepare to repeat the measurement. Re-position and measure the child three times.

11.2. Weight

The SECA 874 scale will be used to weigh infants and children to the nearest 0.1 kg (Seca Corporation, Hanover, MD). Infants and young children can also be weighed simultaneously with their parent or guardian by the unique "mother-baby" function (parent or guardian is weighed and then the infant or child while held by the parent). Body mass index (BMI) will then be calculated later and used to determine the nutritional status of participants as severe malnutrition (BMI <15.9kg/m2), moderate malnutrition (BMI = 16-16.9 kg/m2), mild malnutrition (BMI =17-18.4kg/m2) and normal (BMI> 18.5kg/m2) (WHO, 1995).

Explain to the mother that we want to weigh her child to see how he or she is growing. If she has a baby or a child who is unable or unwilling to stand, she will hold the child on the scale.

Undress the child, removing as much clothing as possible. Explain that the child needs to remove outer clothing and shoes in order to obtain an accurate weight. If the baby is wearing a diaper, the diaper should be removed. If any heavy clothes remain on the child, make a note in the *Comments* column of the exam sheet.

Turn the power on the scale when you are ready to begin weighing.

If the child is less than 2 years old or is unable to stand, you will use the 2 in 1 weighing function (called *tared* weighing).

The **2 in 1** function enables the weight of babies and small children to be determined while an adult holds them.

Press the start key with no load on the scale. The scale is ready for use when it sets to 0.00

If necessary, switch the weight display to KG: hold down the 2 in 1 key for about 3 seconds.

Press the start key with no load on the scale. Wait until the display shows 0.00

Ask the adult to remove her shoes and stand in the middle of the scale (on the footprints) without the child. She may need to adjust any long garments that could cover the display.

After the mother's weight appears on the display, tell her to remain standing on the scale. Press the **2 in 1** key to activate the function.
The scale stores the weight of the adult and the display returns to zero
When 0.00 and NET appear in the display, hand the child to the adult. The scale will determine the weight of the child. Once the value is stable for about 3 seconds, the weight is measured.

The positioner will clearly call out the child's weight to the recorder. Record the child's weight to the nearest 0.01 kg. (x3)

If a mother is very heavy (e.g. more than 100kg) and the baby's weight is relatively low (e.g. less than 2.5kg), the baby's weight may not register on the scale. In such cases, have a lighter person hold the baby on the scale.

To turn off the **2 in 1** function, press the **2 in 1** key.
* The **2 in 1** function remains on until you press the **2 in 1** key again, or until the scale switches off automatically.

If several children are to be weighed consecutively, it is important that it is always the same adult who performs the measurement and that this person's weight does not change (e.g. due to a piece of clothing being removed).

If the child is 2 years or older, you will weigh the child alone if the child will stand still. Talk with the child about the need to stand still. Communicate with the child in a sensitive, non-frightening way.

Press the start key with no load on the scale. The scale is ready for use when it sets to 0.00

Ask the child to stand in the middle of the scale, on the footprints. Once on the scale, the child must stand still. The HOLD function is automatically activated for weights over 1.5kg/3.3lbs. The display flashes until a stable weight has been measured. The display is then frozen until the next weighing operation.

The positioner will clearly call out the child's weight to the recorder. Record the child's weight to the nearest 0.01 kg. (x3)

Note: If the child jumps on the scale or won't stand still, you will need to use the tared weighing procedure instead.

If no further weighing operations are performed, the scale switches off automatically after 2-3 minutes.

11.3. Mid-Upper Arm Circumference

The child's MUAC will be measured at the midpoint between the tip of the shoulder and the tip of the elbow, to the nearest millimeter using TALC's MUAC tape (St Albans, Herts, AL1 5TX, UK) and interpreted according to WHO guidelines. The MUAC tape has red, yellow, and green sections marked in millimeters to help identify malnutrition in both children and adults. Red to yellow is 110 mm and yellow to green is 125 mm. For children 6 months to 5 years of age whose MUAC is in the red section, in other words, less than 110mm, are severely malnourished and in need of an intervention. MUAC readings in the yellow section indicate that the child may need access to a supplementary feeding program. Any child with severe acute malnutrition, defined as a weight for height Z-score <-3.0, or severe chronic malnutrition, defined as a height for age Z score <-3.0 will be referred to a local health clinic for care.

First, **find the midpoint of the upper arm**. Have the child stand up straight with feet together, and the right arm bent 90 degrees at the elbow, palm facing up. The examiner is positioned behind the child. The most upper edge of the posterior border of the acromion process of the scapula is located and marked. Hold the zero end of the measuring tape at this mark and extend the tape down the back of the child's arm to the tip of the olecranon process (the bony part of the mid-elbow). The examiner reads the measurement aloud to the recorder. Keeping the tape in position, locate the spot which is half the distance from the acromion to the olecranon processes (i.e. the midpoint of the upper arm). The recorder will mark the midpoint on the back of the child's arm.

To **measure mid-upper arm circumference**, have the child stand up straight with the arms relaxed at the sides. The examiner will stand facing the child's right side. The measuring tape is placed around the upper arm at the marked point.

Wrap the tape around the arm, pulling it to lie flat against the surface of the skin. Be careful not to pull the tape too tightly (to compress the skin). Look away as you are positioning the tape. When it feels like it's in position, look at the tape and read the number which is on the line of the MUAC tape. **Upon measuring, the examiner will clearly call out the number to the recorder.** Record to the nearest 0.1 cm (0.1 cm = 1 mm). Keeping the child in place, release the MUAC strip, reposition, and measure for a total of 3 measurements.

11.4. Materials for Anthropometry

- ShorrBoard
- seca scale with 1 set of test weights
- MUAC strips (2)
- Chucks and alcohol swabs
- Pens and sharpie markers
- Trash bags
- Extra set of AA batteries (6)
- Clipboard with exam forms

12. Verbal Autopsy

12.1. Verbal Autopsy Interview Protocol

There is a lack of information on cause-specific mortality in developing countries due to poor infrastructure, such as non-existent or poor quality vital registration systems, but this information is important to develop effective health interventions and help facilitate research initiatives to decrease rates of preventable deaths. The data on cause of death are critical for establishing high quality health policy. This enables evaluation of patterns in epidemiology and guidance on allocation of resources that may be in limited supply.

Trained interviewers will conduct verbal autopsies in the local language (Amharic) using the WHO standard verbal autopsy method questionnaire. The questionnaire will be administered to the next of kin of any deceased child under 10 years of age (0 – 9 years) during the study (starting with the baseline census, which was conducted in July 2010) in order to determine the cause of death. Verbal autopsies will be reviewed by two to three masked physicians who will assign the cause of death.

13. Passive Surveillance

Mass oral antibiotics may reduce mortality. For example, the TANA study showed a reduction in childhood mortality in treatment arms that received any mass azithromycin distribution, compared to the delayed treatment arm, which served as an untreated control arm 47. We wish to further assess this potential mortality benefit of mass azithromycin. In the study area, two health extension workers from each kebele keep records on all births and deaths. We propose to study the records of health extension workers from each health post, in order to assess whether mortality was reduced in treatment arms given more frequent mass antibiotic treatments. A data collector will visit each kebele health post to record information from the log books of the health extension workers. Information recorded will include the age, gender, and subkebele location of each newborn and deceased individual, as well as the date of birth or death. The cause of death will also be recorded. No names or personal identifiers will be recorded. All information will be recorded directly into a computer spreadsheet. All available information for the entire study period will be collected. We will then assess whether mortality is reduced in the time period directly following a mass antibiotic distribution, and whether the causes of death differ in different treatment arms.

14. Study Medication

14.1 Study Medication Description (from Pfizer, Inc.)

Zithromax® is supplied for oral administration as film-coated, modified capsular shaped tablets containing azithromycin dehydrate equivalent to either 250mg or 500mg azithromycin and the following inactive ingredients: dibasic calcium phosphate anhydrous, pregelatinized starch, sodium croscarmellose, magnesium stearate, sodium lauryl sulfate, hydroxypropyl methylcellulose, lactose, titanium dioxide, triacetin and D&C red #30 aluminum lake.

Zithromax® for oral suspension is supplied in bottles containing azithromycin dehydrate powder equivalent to 300mg, 600mg, 900mg, or 1200mg azithromycin per bottle and the following inactive ingredients: sucrose; sodium phosphate, tribasic, anhydrous; hydroxypropyl cellulose; xanthan gum; FD&C Red #40; and spray dried artificial cherry, crème de vanilla and banana flavors. After constitution, each 5mL of suspension contains 100mg or 200mg of azithromycin.

14.2 Dosage Information

Azithromycin will be administered as a single dose, in tablet form for adults and in oral suspension form for children. Dosing will be as per the WHO recommendations for treatment of active trachoma:

- Single dose of one gram of azithromycin for adults
- Single dose of 20mg/kg in children (up to the maximum adult dose of 1g)
- Height-based dosing of children will be acceptable, as per The Carter Center's program—note that this is supported by the WHO PBD group.

Individuals who are either under the age of 6 months, pregnant, or allergic to macrolides/azalides will be treated with 1% tetracycline eye ointment to be applied twice daily to both eyes for a 6 week period. If the appropriate ethical committee in Ethiopia

suggests pregnancy tests for self-reported pregnant women, or if the women are unsure of their pregnancy status, then they may be offered an on-site pregnancy test.

14.3 Adverse Reactions/Side Effects

Approximately 10 million doses of oral azithromycin have now been distributed for trachoma, and reports of serious side effects are essentially non-existent. This may be due in part to minimal surveillance. It also may be due to the fact these are extremely rare with a single dose of azithromycin. In fact, where carefully monitored, there were actually fewer GI side effects after taking azithromycin. We will create a network to identify any possible post-treatment serious adverse effects.

Azithromycin is generally well-tolerated. The most common side effects of azithromycin and erythromycin are diarrhea or loose stools, nausea, abdominal pain, and vomiting, each of which may occur in fewer than one in twenty persons who receive azithromycin. Rarer side effects include abnormal liver function tests, allergic reactions, and nervousness. Diarrhea due to *Clostridium difficile* has been rarely reported.

The **adverse reactions** that may occur after taking azithromycin will be explained to individuals prior to enrollment in this study. All individuals who have been given azithromycin will be told to immediately communicate side-effects to local health extension workers, who will relay the message to representatives of The Carter Center program, which will ensure that appropriate medical care will be provided, and that the frequency and severity of adverse events can be assessed.

14.4 Treatment Costs

The azithromycin (Zithromax®) has been donated by the Pfizer Corporation free of charge. There will be no costs to acquiring the study medication.

14.5 Alternate Therapies (Control Villages)

Control arms will be administered azithromycin immediately after they are swabbed.

14.5.1 Tetracycline Ophthalmic Ointment

Tetracycline ophthalmic ointment (1%) is the current standard treatment in Ethiopia for ocular trachoma, and will be distributed to study patients who are not eligible to receive azithromycin.

14.6 Treatment/Monitoring Schedule

The treatment and monitoring schedule for all study arms is shown in Table 2.

14.7 Medication Procurement/Donation

Pfizer, Inc. will provide the donation of Zithromax® (azithromycin), which will be shipped directly to Ethiopia and received by a representative of the Ethiopian Ministry of Health, who will manage the customs process and transport the medication from the port to a storage site. The exemption of duties and taxes will be settled by the Ethiopian Customs Authorities and the Ethiopian Ministry of Health.

14.8 Study Medication Storage and Accountability

Zithromax® tablets will be stored between 15° to 30°C (59° to 86°F), as recommended by the Pfizer Labs. A record of the exact number of tablets distributed and quantity of oral suspension dispensed will be kept by The Carter Center treatment distribution team.

14.9 Medication Quality Control

Study medication will be stored in The Carter Center project office prior to use. The ophthalmic assistant and the study coordinator will regularly check and record the study medication expiry dates. The expiration dates on the medication containers will be strictly monitored and all expired study medicine will be discarded appropriately.

14.10 Checking Antibiotic Coverage

In order to observe the antibiotic coverage of participating state teams, the ophthalmic nurses and public health nurses who participate in the antibiotic distribution will mark the dorsal aspect of the right hand of all treated individuals in a randomly chosen number of state teams. The treatment distribution team will then return to the treated state team the following afternoon or the next day, to survey every household to determine the number of marked individuals in each state team. This number is then compared to the total population in each state team from the census to establish an antibiotic coverage level. The return visit to the treated state teams provides an opportunity to identify and treat any individuals who missed the antibiotic distribution initially.

15. Protection of Human Subjects

15.1 Internal Review Board Approval

As part of the trachoma elimination program, the registration process of azithromycin by the Drug Administration and Control Authority, Ethiopia, has already been finished. The mass distribution of the azithromycin plan is prepared together with the Zonal and Regional Health authorities of the Southern Nations Nationalities and Peoples Region (SNNPR).

15.1.1 UCSF Committee on Human Research

The University of California, San Francisco, Committee on Human Research will annually review study protocol for ethical approval.

15.1.2 Ethiopian Ministry of Science and Technology

The study protocol will be reviewed and granted ethical approval by the Science and Technology Commission of Ethiopia before any study activities begin.

15.1.3 Emory University Institutional Review Board

The Emory University Institutional Review Board will annually review study protocol for ethical approval.

15.2 Informed Consent

The chairman of each state team will be asked for permission to include the state team in the study. Additionally, the study will be discussed with all adult family members in the state teams by the participating Carter Center staff members who speak Amharic or other local language.

At each collection visit, all adults 18 years of age and older will be informed about the possible risks and benefits of the treatment and swab taking, and asked to give a verbal consent. Young adults and children below 18 years of age, who cannot give consent by law, will be included in the study only following the receipt of verbal informed consent from a parent or guardian. Verbal assent will be obtained by any child over the age of 7. If, at any time, a parent or guardian elects to withdraw themselves or a family member from the study, it will be made clear that they will be offered the same medical treatment outside the study.

Blood testing: Blood testing will be approximately as uncomfortable as a pin prick to the finger. The major risk of this procedure is infection of the puncture site, though using

aseptic technique this rarely occurs. These communities are familiar with the pinprick test because children who present at a health center with fever are offered the pinprick for a malaria smear.

Anthropometric measurements: There are very minimal risks associated with the measuring board, scale, or MUAC tapes aside from minimal anxiety during the measurements. Examiners will do their best to ensure that the parent or guardian of the child understands the process of assessing anthropometric measurements. The examiners will attempt to minimize discomfort for all study participants before, during, and after the measurements are taken. Children with wasting, stunting, malaria, or anemia will be referred for appropriate treatment, which will typically be to the nearest health center.

Verbal autopsy: As verbal autopsy requests the respondent to answer questions about a deceased child, it is possible that he or she will experience emotional stress and grief related to the death of the child. Interviewers will be trained to address these situations appropriately. If the respondent is in need of a mental health intervention, referrals will be made by the interviewer.

15.3 Adequacy of Protection Against Risk

There are several layers of procedures to help minimize study-associated risk to participants. The risk of antibiotic treatment will be minimized by treating only those who fit in the approved age and inclusion category, as well as by regularly scheduled follow-up examinations by a trained trachoma grader. Should the antibiotic be ineffective to an individual, the study medication will be discontinued for them. In the event of any adverse effects, appropriate medical care will be provided by the local health center.

15.4 Inclusion of Pregnant Women & Children

All participants, regardless of gender, will be accepted. Pregnant women will be excluded from receiving oral azithromycin, and will be offered topical tetracycline eye ointment in its place.

All participants will be included in the study. Appropriate verbal assent will be obtained from all participants over the age of 10 entering the study. Appropriate verbal consent will be obtained from parent/guardians of children <18 years of age entering the study. (See Appendix 13.1)

15.5 Compensation to Participants

There is no cost to the participant and there is no reimbursement for overall participation in this study. Each participant will receive free ophthalmic examinations during the course of the study.

16. Data Collection & Management

A census will be conducted in the study area before distribution of the drug in order to more accurately carry out treatment (e.g. to avoid having people come from neighboring areas for treatment). Seventy-two state teams will be randomized between the 6 study arms: Treatment completed, Treatment continued , Annual Treatment, Biannual Treatment, Clinically Active-Identified Treatment, Lab-Identified Treatment.

In order to standardize procedures among the examiners, training will be given in advance on the grading system and the swab collection protocol. The Carter Center Study Coordinator, with help from the Proctor Study Coordinators, will be responsible for the overall day-to-day supervision of the census, collection and treatment teams in the field, as well as the completion of all forms and proper recording, storage and transport of study samples.

The baseline visit began with the enumeration/census taking of each enrolled state team, followed by examination and swab collection in each state team assigned for monitoring at baseline. In order to ensure time comparability, the team will work to complete collection for each time point of the study in 2-6 weeks. After the examination/collection phase is completed, the distribution of azithromycin will be completed (according to study protocol) in 2-3 weeks time.

All census and treatment data will be double-entered in Ethiopia by TCCE Data Coordinator and team within 1 month of return from the field. All data from the exam sheets (names of state team and sub-kebele, collection date, random number, clinical exam result, gender, age and any quality controls, such as duplicate and/or negative field swabs) will be double-entered at Proctor by the Database Specialist and temporary support staff within 1 month of return from the field. PCR results will be double-entered at Proctor within 1 week of obtaining results. Any inconsistencies will be resolved by the appropriate study coordinator (The Carter Center or Proctor), and if necessary, by consultation with the appropriate examiner or lab personnel. Patient names will not be made available to UCSF personnel: individuals will be identified by ID number only. Data will be backed up upon entry, and forms will be kept in locked cabinets through the conclusion of the study.

As in preliminary studies, quality assurance will include negative field control swabs taken on 5% of participants. Pooling requires knowledge of which samples are controls and which are not. The Proctor database manager is responsible for organizing the samples for the pooling plan, and the lab specialist processes PCR, including the pooling work. Coverage levels will be measured directly, relative to the census.

16.1 Census Administration

Basic protocols for census taking are available in the Amharic language.

16.1.1 Form Description

The census form that will be used in this study will be prepared by The Carter Center Ethiopia and printed in Amharic. It will contain columns for the full name of the individuals, age, and gender, as well as columns for assigning household number and individual numbers for further identification of individuals within each household.

16.1.2 Completing the Census Form

The census form will be completed in Amharic by a census team selected by The Carter Center Ethiopia. The census team will visit each state team and inform the chairman to alert each head of household in the village. Each head of household will be interviewed to provide the full name, age, and gender of each member of the household. Each household will be assigned a number and each individual within a household will also be assigned a number.

16.2 Field Data Collection

16.2.1 Registration Form

The Registration Form is prepared by The Carter Center and contains the full spelling of each individual's name, age, gender and household number, taken directly from the census.

16.2.2 Field Form

The Field Form contains columns for placing the random numbered stickers for each sample collected in the field, age, gender, and the clinical exam results for each participant. Sub-kebele and state team name, date, GPS location, and the names of examination team members are also included at the top of each form.

16.3 Data Forms Management

All completed Registration Forms, Examination Forms and Treatment/Census books will be given to The Carter Center Study Coordinator at the end of each workday and stored in a safe, secure place for transport to Addis Ababa. The Study Coordinator will check the forms for completeness and accuracy.

- **Treatment/Census Books:** Treatment coverage calculated and recorded, books organized and stored in secure place at TCCE office in Addis Ababa.
- **Exam forms** should be immediately photocopied, with one copy stored at TCCE, and the other transported to the Proctor Foundation for double data entry under supervision of the Database Manager. .
- **Registration Forms:** Forms have patient information/names and will be organized by TCCE Study Coordinator and stay in Ethiopia at TCCE office, stored in secure, locked area.

16.3.1 Data Editing

Before data entry, the forms will be reviewed and cross-checked for consistency and completeness. If the forms are not filled out completely, the Study Coordinator (TCCE or Proctor) will contact the person responsible for completing the form to provide missing data or clarify any inconsistent data. The Study Coordinator is the only person who is authorized to add missing data or make any changes to the study forms. All changes will be made in a different color ink and initialed and dated.

16.3.2 Data Entry and Quality Control

The treatment data collected in the field will be entered into a database at The Carter Center Ethiopia office in Addis Ababa. A double entry will be done to minimize errors. The study coordinator will do the final editing. The collection fieldwork data will be entered into a database by Proctor Foundation's Database Manager, who will also oversee the work of the staff member helping to double-enter the data. The Proctor Database Manager will also enter all PCR results into the database.

16.3.3 Adjudication of Double Data Entry and Data Entry Errors

Double data entry will be conducted and adjudicated at the Carter Center in Addis Ababa to minimize data entry errors. All errors will be recorded.

16.3.4 Data Consistency & Validity

Through range checks, the data entry software ensures to a large extent that there are no inconsistencies or invalid data. Data will also be checked by the Database Manager for consistency and errors.

Specific software has been developed to verify consistency. The software will create an error file with relevant data such as the form identification, field names and data. The database manager will then consults the forms, resolve the inconsistency and enter the correct data. The corrected consistency error will then be electronically merged with the full database to give the corrected file.

16.4 Data Security and Storage

Access to any patient information will be protected by a password or locked in a secure storage room. All files are password protected and saved on a secure server, which is backed up each business day at Proctor. The Carter Center will maintain at least two sets of back-up CDs at any particular time, kept in a place different from the computer center.

Forms will be kept until the conclusion of the study. The DSMC can make requests to have access to the data at any point during the course of the study.

16.5 Data Analysis Estimation of disease prevalence

Computerized randomization is utilized to prepare samples for pooling. PCR pooling will be conducted and results tabulated by state team. For PCR tests (at baseline), the prevalence in 0-≤9 year olds (2-pool: due to higher disease higher prevalence) and in those ≥ 10 (5-pool: lower disease prevalence) is determined directly. For pooled results, we use maximum likelihood estimation to determine what prevalence in the population (by the 2 age-groups, by state team) has the highest likelihood of resulting in the observed pooled results (the likelihood is a relatively simple function of the number of positive and negative 5-pools, positive and negative 4-pools, etc., with appropriate combinatorics) Note that this is a modification from the grant text (Equation 1, page 37: this equation can only provide an estimate if all pools are made up of the same number of samples).

16.6 Data Management, Security and Quality Assurance

Please see SAP sections 7 and 8.

16.7 Sample Organization and Storage

Once samples arrive at the Carter Center, the Study Coordinator uses an electronic data capture system (Brady Code Reader 3.0) to scan the random number stickers on examination sheets. Random number stickers on the sample tubes are also scanned. Both are saved as Excel files and STATA statistical software is then used to double-check the two files for completeness and accuracy.

Samples are stored at The Carter Center in a -20° freezer. Samples are organized according to collection time point.

17. Statistical Analyses

Specific Aim I is to be addressed by comparing baseline infection rates in 0-9 year-old children in communities in arms **Stop-Annual and Stop-Biannual** with rates at 36 months, using a paired-sample test (see below for details).

Specific Aim II is to be addressed by comparing 36-month infection rates in all individuals in communities in arms **Continue-Annual** with 36-month infection rates in all individuals in communities in **Continue-Biannual** (see below for details).

Specific Aim III is to be addressed by comparing 36-month infection rates in children in arms **Target-Exam** and **Target-Age** (see below for details).

Specific Aim 1

For Aim 1, we propose to conduct a single paired T-test for all communities aggregated together, whether from **Stop-Annual** or from **Stop-Biannual**, transforming the prevalence if needed by the square or cube root to achieve approximate normality (square-root transformation was performed in TANA I). Prespecified analyses will also examine each arm **Stop-Annual** and **Stop-Biannual** separately, and together using ANOVA (including whether or not the community had come from the **Stop-Annual** or the **Stop-Biannual** prior to cessation of mass administration).

Residual plots (versus baseline value, versus predicted values and a Q-Q plot) will be investigated for outliers, nonlinearity, non-homogeneous variances and gross violations of normality.

Specific Aim 2

For Aim 2, we propose to conduct a single unpaired T-Test comparing arms **Continue-Annual** and **Continue-Biannual** at 36 months, including (1) treatment arm (annual or biannual) and (2) the prevalence value at the beginning of TIRET as the two covariates. Specifically, the outcome variable is the 36-month prevalence of infection among children, in each community. We anticipate using square-root transformed prevalence.

Residual plots (versus baseline value, versus predicted values and a Q-Q plot) will be investigated for outliers, nonlinearity, non-homogeneous variances and gross violations of normality.

Note that it is possible that both arms will show zero prevalence by the end of the study, indicating that both methods are successful in eliminating infection according to our measure. We consider this, in itself, to be an interesting result. Since comparison of the final zero prevalences is not a meaningful one, in this case the primary analysis will compare the time to elimination (defined specifically as the time between the beginning of the TIRET study and the first estimated zero-prevalence visit) between the two arms using a Wilcoxon rank-sum test. Also note that no-identifiable-infection in any of the communities is itself a very interesting result.

Specific Aim 3

For Aim 3, we propose to conduct a single ANCOVA comparing arms **Target-Exam** and **Target-Age** at 36 months, including (1) treatment arm (household targeting vs all preschool) and (2) the prevalence value at the beginning of TIRET as the two covariates. Specifically, the outcome variable is the 36-month prevalence of infection among children, in each community. The predictors are the estimated community-specific prevalence at the beginning of TIRET (in children), and the treatment arm. The test statistic will be the T-statistic for the regression coefficient for treatment arm, and matching will be accounted for.

As before, residual plots (versus baseline value, versus predicted values and a Q-Q plot) will be investigated for outliers, nonlinearity, non-homogeneous variances and gross violations of normality.

Note that as in Aim 2, it is possible that both arms will show zero prevalence by the end of the study, indicating that both methods are successful in completely eliminating infection according to our measure. In this case, the primary analysis will compare the time to elimination (defined specifically as the time between the beginning of the TIRET study and the first estimated zero-prevalence visit) between the two arms using a Wilcoxon rank-sum test.

17.1 Calculation of Sample Size & Power Analysis

Please see SAP section 5.1.

17.2 Inter-observer Variability

Although inter-observer variability is a large concern with grading trachoma in the field, it should be noted that the clinical exam is NOT an outcome of this study. For other, programmatic purposes, inter-observer variability will be estimated by having all examiners grade a series of patients, masked to their colleagues' grading. The clinical examination in the field will be made by trained and qualified trachoma graders (public health nurses, ophthalmic assistants, and ophthalmic nurses), who have undergone a formal training session (and, in most cases, a number of refresher sessions) and have been selected to work in the field based on their grading accuracy.

Lab tests will include a negative field control on 5% of individuals (blue control). Previously we have found these to be ~99.1% negative, indicating a small amount of field contamination or false positive PCR testing—note that the specificity of the Amplicor test is estimated to be ~99.5%.

18. Appendix

(Please see attached documents)

- **18.1 Patient Verbal Consent Scripts**
- **18.2 Census Form**
- **18.3 Randomized List for Each Visit (The Carter Center)**
- **18.4 Field Form (Swabbing)**

19. References

1. McClarty G. Chlamydial metabolism as inferred from the complete genome sequence. In: Stephens R, ed. *Chlamydia: intracellular biology, pathogenesis, and immunity*. Washington, DC: ASM Press; 1999:69-100.

2. Peeling R, Brunham RC. Chlamydiae as pathogens: new species and new issues. *Emerging infectious diseases.* 1996;2:307-319.

3. Hackstadt T. Cell Biology. In: Stephens R, ed. *Chlamydia: intracellular biology, pathogenesis, and immunity*. Washington, DC: ASM Press; 1999:101-138.

4. Sanford-Smith J. Trachoma. *Eye diseases in hot climates*. Oxford: Butterworth Heinemann; 1997:105-120.

5. Dean D, Stephens RS. Identification of individual genotypes of Chlamydia trachomatis from experimentally mixed serovars and mixed infections among trachoma patients. *Journal of clinical microbiology.* 1994;32:1506-1510.

6. Schachter J. Infection and disease epidemiology. In: Stephens R, ed. *Chlamydia: intracellular biology, pathogenesis, and immunity*. Washington, DC: ASM Press; 1999:139-169.

7. Emerson P, Lindsay SW, Alexander N, Bah M, Dibba SM, Faal HB, Lowe KO, McAdam KP, Ratcliffe AA, Walraven GE, Bailey RL. Role of flies and provision of latrines in trachoma control: cluster-randomised controlled trial. *Lancet.* 2004;363:1093-1098.

8. Miller K, Pakpour N, Yi E, Melese M, Alemayehu W, Bird M, Schmidt G, Cevallos V, Olinger L, Chidambaram J, Gaynor B, Whitcher J, Lietman T. Pesky trachoma suspect finally caught. *British Journal of Ophthalmology.* 2004;88(6):750-751.

9. Gower E, Solomon AW, Burton MJ, Aguirre A, Muñoz B, Bailey R, Holland M, Makalo P, Massae P, Mkocha H, Mabey DC, West SK. Chlamydial positivity of nasal discharge at baseline is associated with ocular chlamydial positivity 2 months following azithromycin treatment. *Invest Ophthalmol Vis Sci.* 2006;47:4767-4771.

10. Lee S, Alemayehu W, Melese M, Lakew T, Lee D, Yi E, Cevallos V, Donnellan C, Zhou Z, Chidambaram JD, Gaynor BD, Whitcher JP, Lietman TM. Chlamydia on children and flies after mass antibiotic treatment for trachoma. *American Journal of Tropical Medicine and Hygiene.* 2007;76:129-131.

11. Zerihun N. Trachoma in Jimma zone, south western Ethiopia. *Tropical Medicine and International Health.* 1997;2(12):1115-1121.

12. West S, Munoz B, Turner VM, Mmbaga BBO, Taylor HR. . The epidemiology of trachoma in central Tanzania. *International journal of epidemiology.* 1991;20(4):1088-1092.

13. Tielsch J, West KP Jr, Katz J, Keyvan-Larijani E, Tizazu T, Schwab L, Johnson GJ, Chirambo MC, Taylor HR. The epidemiology of trachoma in southern Malawi. *American Journal of Tropical Medicine and Hygiene.* 1988;38:393-399.

14. Luna E, Medina NH, Oliveira MB, de Barros OM, Vranjac A, Melles HH, West S, Taylor HR. Epidemiology of trachoma in Bebedouro State of São Paulo, Brazil: prevalence and risk factors. *International journal of epidemiology.* 1992;21:169-177.

15. West S, Muñoz B, Lynch M, Kayongoya A, Mmbaga BB, Taylor HR. Risk factors for constant, severe trachoma among preschool children in Kongwa, Tanzania. *American journal of epidemiology.* 1996;143:73-78.

16. Sahlu T, Larson C. The prevalence and environmental risk factors for moderate and severe trachoma in southern Ethiopia. *Journal of Tropical Medicine and Hygiene.* 1992;95:36-41.

17. Ngondi J, Onsarigo A, Adamu L, Matende I, Baba S, Reacher M, Emerson P, Zingeser J. The epidemiology of trachoma in Eastern Equatoria and Upper Nile States, southern Sudan. *Bulletin of the World Health Organization.* 2005;83:904-912.

18. West S, Congdon N, Katala S, Mele L. Facial cleanliness and risk of trachoma in families. *Archives of ophthalmology.* 1991;109(6):855-857.

19. Taylor H, West SK, Mmbaga BB, Katala SJ, Turner V, Lynch M, Muñoz B, Rapoza PA. Hygiene factors and increased risk of trachoma in central Tanzania. *Archives of Ophthalmology.* 1989;107:1821-1825.

20. Ngondi J, Matthews F, Reacher M, Onsarigo A, Matende I, Baba S, Brayne C, Zingeser J, Emerson P. Prevalence of Risk Factors and Severity of Active Trachoma in Southern Sudan: An Ordinal Analysis. *American Journal of Tropical Medicine and Hygiene.* 2007;77:126-132.

21. Schémann J, Sacko D, Malvy D, Momo G, Traore L, Bore O, Coulibaly S, Banou A. Risk factors for trachoma in Mali. *International journal of epidemiology.* 2002;31:194-201.

22. Abdou A, Nassirou B, Kadri B, Moussa F, Munoz BE, Opong E, West SK. Prevalence and risk factors for trachoma and ocular Chlamydia trachomatis infection in Niger. *British Journal of Ophthalmology.* 2007;91:13-17.

23. Schémann J, Guinot C, Ilboudo L, Momo G, Ko B, Sanfo O, Ramde B, Ouedraogo A, Malvy D. Trachoma, flies and environmental factors in Burkina Faso. *Transactions of the Royal Society of Tropical Medicine and Hygiene.* 2003;97:63-68.

24. Taylor H, Velasco FM, Sommer A. The ecology of trachoma: an epidemiological study in southern Mexico. *Bulletin of the World Health Organization.* 1985;63:559-567.

25. Baggaley R, Solomon AW, Kuper H, Polack S, Massae PA, Kelly J, Safari S, Alexander ND, Courtright P, Foster A, Mabey DC. Distance to water source and altitude in relation to active trachoma in Rombo district, Tanzania. *Tropical Medicine and International Health.* 2006;11:220-227.

26. Katz J, West KP Jr, Khatry SK, LeClerq SC, Pradhan EK, Thapa MD, Ram Shrestha S, Taylor HR. Prevalence and risk factors for trachoma in Sarlahi district, Nepal. *British Journal of Ophthalmology.* 1996;80:1037-1041.

27. Bailey R, Downes B, Downes R, Mabey D. Trachoma and water use; a case control study in a Gambian village. *Transactions of the Royal Society of Tropical Medicine and Hygiene.* 1991;85:824-828.

28. Taylor H. A simple method for assessment of association between synanthropic flies and trachoma. *American Journal of Tropical Medicine and Hygiene.* 1988;38:623-627.

29. Cumberland P, Hailu G, Todd J. Active trachoma in children aged three to nine years in rural communities in Ethiopia: prevalence, indicators and risk factors. *Transactions of the Royal Society of Tropical Medicine and Hygiene.* 2005;99:120-127.

30. Brechner R, West S, Lynch M. Trachoma and flies. Individual vs. environmental risk factors. *Archives of ophthalmology.* 1992;110:687-689.

31. Burton M, Holland MJ, Makalo P, Aryee EA, Alexander ND, Sillah A, Faal H, West SK, Foster A, Johnson GJ, Mabey DC, Bailey RL. Re-emergence of Chlamydia trachomatis infection after mass antibiotic treatment of a trachoma-endemic Gambian community: a longitudinal study. *Lancet.* 2005;365:1321-1328.

32. West S, Muñoz B, Lynch M, Kayongoya A, Chilangwa Z, Mmbaga BB, Taylor HR. Impact of face-washing on trachoma in Kongwa, Tanzania. *Lancet.* 1995;345:155-158.

33. Alemayehu W, Melese M, Fredlander E, Worku A, Courtright P. Active trachoma in children in central Ethiopia: association with altitude. *Transactions of the Royal Society of Tropical Medicine and Hygiene.* 2005;99:840-843.

34. Ezz al Arab G, Tawfik N, El Gendy R, Anwar W, Courtright P. The burden of trachoma in the rural Nile Delta of Egypt: a survey of Menofiya governorate. *The British journal of ophthalmology.* 2001;85:1406-1410.

35. Taylor H, Rapoza PA, West S, Johnson S, Munoz B, Katala S, Mmbaga BBO. The epidemiology of infection in trachoma. *Investigative Ophthalmology and Visual Science.* 1989;30:1823-1833.

36. Schachter J, West SK, Mabey D, Dawson CR, Bobo L, Bailey R, Vitale S, Quinn TC, Sheta A, Sallam S, Mkocha H, Mabey D, Faal H. Azithromycin in control of trachoma. *Lancet.* 1999;354:630-635.

37. West E, Munoz B, Mkocha H, Holland MJ, Aguirre A, Solomon AW, Bailey R, Foster A, Mabey D, West SK. Mass treatment and the effect on the load of Chlamydia trachomatis infection in a trachoma-hyperendemic community. *Investigative Ophthalmology and Visual Science.* 2005;46:83-87.

38. Solomon AW, Holland MJ, Alexander ND, et al. Mass treatment with single-dose azithromycin for trachoma. *The New England journal of medicine.* Nov 4 2004;351(19):1962-1971.

39. Bailey R, Duong T, Carpenter R, Whittle H, Mabey D. The duration of human ocular Chlamydia trachomatis infection is age dependent. *Epidemiol Infect.* 1999;123:479-486.

40. Solomon A, Holland MJ, Alexander ND, Massae PA, Aguirre A, Natividad-Sancho A, Molina S, Safari S, Shao JF, Courtright P, Peeling RW, West SK, Bailey RL, Foster A, Mabey DC. Mass treatment with single-dose azithromycin for trachoma. *New England Journal of Medicine.* 2004;351:1962-1971.

41. Yorke J, Hethcote HW, Nold A. Dynamics and control of the transmission of gonorrhea. *Sexually Transmitted Diseases.* 1978;5:51-56.

42. Plummer F, Nagelkerke NJ, Moses S, Ndinya-Achola JO, Bwayo J, Ngugi E. The importance of core groups in the epidemiology and control of HIV-1 infection. *AIDS.* 1991;5(Suppl 1):S169-S176.

43. May R. The transmission and control of gonorrhea. *Nature.* 1981;291:376-377.

44. Thomas J, Tucker MJ. The development and use of the concept of a sexually transmitted disease core. *Journal of Infectious Diseases.* 1996;174(Suppl 2):S134-S143.

45. House JI, Ayele B, Porco TC, et al. Assessment of herd protection against trachoma due to repeated mass antibiotic distributions: a cluster-randomised trial. *Lancet.* Mar 28 2009;373(9669):1111-1118.

46. West S. Blinding trachoma: prevention with the SAFE strategy. *Am J Trop Med Hyg.* 2003;69(Suppl 5):18-23.

47. Emerson P, Burton M, Solomon AW, Bailey R, Mabey D. The SAFE strategy for trachoma control: using operational research for policy, planning, and implementation. *Bulletin of the World Health Organization.* 2006;84:613-619.

48. Bowman R, Sillah A, Van Dehn C, Goode VM, Muqit MM, Johnson GJ, Milligan P, Rowley J, Faal H, Bailey RL. Operational comparison of single-dose azithromycin and topical tetracycline for trachoma. *Investigative Ophthalmology and Visual Science.* 2000;41:4074-4079.

49. Bailey R, Arullendran P, Whittle HC, Mabey DCW. Randomized controlled trial of single-dose azithromycin in treatment of trachoma. *Lancet.* 1993;342:453-456.

50. Dawson C, Schachter J, Sallam S, Sheta A, Rubinstein RA, Washton H. A comparison of oral azithromycin with topical oxytetracycline/polymyxin for the treatment of trachoma in children. *Clin Infect Dis.* 1997;24:363-368.

51. Tabbara K, Abu-el-Asrar A, al-Omar O, Choudhury AH, al-Faisal Z. Single-dose azithromycin in the treatment of trachoma. *Ophthlamology.* 1996;103:842-846.

52. Tabbara K, al-Kharashi SA, al-Mansouri SM, al-Omar OM, Cooper H, el-Asrar AM, Foulds G. Ocular levels of azithromycin. *Archives of ophthalmology.* 1998;116:1625-1628.
53. Lode H, Borner K, Koeppe P, Schaberg T. Azithromycin--review of key chemical, pharmacokinetic and microbiological features. *Journal of Antimicrobial Chemotherapy.* 1996;37 (Suppl C):1-8.
54. Karcioglu Z, El-Yazigi A, Jabak MH, Choudhury AH, Ahmed WS. Pharmacokinetics of azithromycin in trachoma patients: serum and tear levels. *Ophthalmology.* 1998;105:658-661.
55. Whitty C, Glasgow KW, Sadiq ST, Mabey DC, Bailey R. Impact of community-based mass treatment for trachoma with oral azithromycin on general morbidity in Gambian children. *Pediatr Infect Dis J.* 1999;18:955-958.
56. Ruuskanen O. Safety and tolerability of azithromycin in pediatric infectious diseases: 2003 update. *Pediatric Infectious Disease Journal.* 2004;23(2 Suppl):S135-S139.
57. West S, Muñoz B, Bobo L, Quinn TC, Mkocha H, Lynch M, Mmbaga BB, Viscidi R. Nonocular Chlamydia infection and risk of ocular reinfection after mass treatment in a trachoma hyperendemic area. *IOVS.* 1993;34:3194-3198.
58. Holm SO, Jha HC, Bhatta RC, et al. Comparison of two azithromycin distribution strategies for controlling trachoma in Nepal. *Bulletin of the World Health Organization.* 2001;79(3):194-200.
59. Schachter J, West SK, Mabey D, et al. Azithromycin in control of trachoma. *Lancet.* Aug 21 1999;354(9179):630-635.
60. West S, Munoz B, Mkocha H, Holland MJ, Aguirre A, Solomon AW, Foster A, Bailey RL, Mabey DC. Infection with Chlamydia trachomatis after mass treatment of a trachoma hyperendemic community in Tanzania: a longitudinal study. *Lancet.* 2005;366:1296-1300.
61. Burton MJ, Holland MJ, Makalo P, et al. Re-emergence of Chlamydia trachomatis infection after mass antibiotic treatment of a trachoma-endemic Gambian community: a longitudinal study. *Lancet.* Apr 9-15 2005;365(9467):1321-1328.
62. Chidambaram JD, Alemayehu W, Melese M, et al. Effect of a single mass antibiotic distribution on the prevalence of infectious trachoma. *Jama.* Mar 8 2006;295(10):1142-1146.
63. Melese M, Chidambaram JD, Alemayehu W, et al. Feasibility of eliminating ocular Chlamydia trachomatis with repeat mass antibiotic treatments. *Jama.* Aug 11 2004;292(6):721-725.
64. Gaynor BD, Miao Y, Cevallos V, et al. Eliminating trachoma in areas with limited disease. *Emerging infectious diseases.* May 2003;9(5):596-598.
65. West S, Munoz B, Mkocha H, Gaydos C, Quinn T. Trachoma and ocular Chlamydia trachomatis were not eliminated three years after two rounds of mass treatment in a trachoma hyperendemic village. *IOVS.* 2007;48:1492-1497.
66. Melese M, Alemayehu W, Lakew T, et al. Comparison of annual and biannual mass antibiotic administration for elimination of infectious trachoma. *Jama.* Feb 20 2008;299(7):778-784.
67. Lietman T, Porco T, Dawson C, Blower S. Global elimination of trachoma: how frequently should we administer mass chemotherapy? *Nature medicine.* May 1999;5(5):572-576.
68. Solomon A, Burton M. What's new in azithromyin? *Community eye health / International Centre for Eye Health.* Dec 2004;17(52):54-56.
69. West SK, Munoz B, Mkocha H, et al. Infection with Chlamydia trachomatis after mass treatment of a trachoma hyperendemic community in Tanzania: a longitudinal study. *Lancet.* Oct 8 2005;366(9493):1296-1300.
70. Lakew T, House J, Hong KC, et al. Reduction and return of infectious trachoma in severely affected communities in Ethiopia. *PLoS Negl Trop Dis.* 2009;3(2):e376.
71. Solomon AW, Zondervan M, Kuper H, Buchan JC, Mabey DCW, Foster A. *Trachoma control: a guide for programme managers*. Geneva: World Health Organization; 2006.
72. *Primary Health Care Level Management of Trachoma (WHO/PBL/93.33)*. Geneva: World Health Organization; 1993.
73. Burton MJ, Holland MJ, Faal N, et al. Which members of a community need antibiotics to control trachoma? Conjunctival Chlamydia trachomatis infection load in Gambian villages. *Investigative ophthalmology & visual science.* Oct 2003;44(10):4215-4222.
74. Katz J, Zeger SL, Tielsch JM. Village and household clustering of xerophthalmia and trachoma. *Int J Epidemiol.* Dec 1988;17(4):865-869.
75. Bailey R, Osmond C, Mabey DC, Whittle HC, Ward ME. Analysis of the household distribution of trachoma in a Gambian village using a Monte Carlo simulation procedure. *Int J Epidemiol.* Dec 1989;18(4):944-951.
76. Courtright P, Sheppard J, Lane S, Sadek A, Schachter J, Dawson CR. Latrine ownership as a protective factor in inflammatory trachoma in Egypt. *Br J Ophthalmol.* Jun 1991;75(6):322-325.
77. Polack SR, Solomon AW, Alexander ND, et al. The household distribution of trachoma in a Tanzanian village: an application of GIS to the study of trachoma. *Trans R Soc Trop Med Hyg.* Mar 2005;99(3):218-225.
78. Laming AC, Currie BJ, DiFrancesco M, Taylor HR, Mathews JD. A targeted, single-dose azithromycin strategy for trachoma. *Med J Aust.* Feb 21 2000;172(4):163-166.
79. Atik B, Thanh TT, Luong VQ, Lagree S, Dean D. Impact of annual targeted treatment on infectious trachoma and susceptibility to reinfection. *Jama.* Sep 27 2006;296(12):1488-1497.

80. Schemann JF, Guinot C, Traore L, et al. Longitudinal evaluation of three azithromycin distribution strategies for treatment of trachoma in a sub-Saharan African country, Mali. *Acta Trop.* Jan 2007;101(1):40-53.
81. Solomon AW, Peeling RW, Foster A, Mabey DC. Diagnosis and assessment of trachoma. *Clinical microbiology reviews.* Oct 2004;17(4):982-1011, table of contents.
82. Dean D, Palmer L, Pant CR, Courtright P, Falkow S, O'Hanley P. Use of a Chlamydia trachomatis DNA probe for detection of ocular chlamydiae. *Journal of clinical microbiology.* 1989;27:1062-1067.
83. Miyashita N, Matsumoto A, Niki Y, Matsushima T. Evaluation of the sensitivity and specificity of a ligase chain reaction test kit for the detection of Chlamydia trachomatis. *J Clin Pathol.* 1996;49:515-517.
84. Bailey R, Hampton TJ, Hayes LJ, Ward ME, Whittle HC, Mabey DC. Polymerase chain reaction for the detection of ocular chlamydial infection in trachoma-endemic communities. *J Infect Dis.* 1994;170:709-712.
85. Mahony J, Luinstra KE, Sellors JW, Chernesky MA. Comparison of plasmid- and chromosome-based polymerase chain reaction assays for detecting Chlamydia trachomatis nucleic acids. *Journal of clinical microbiology.* 1993;31:1753-1758.
86. Solomon A, Holland MJ, Burton MJ, West SK, Alexander ND, Aguirre A, Massae PA, Mkocha H, Muñoz B, Johnson GJ, Peeling RW, Bailey RL, Foster A, Mabey DC. Strategies for control of trachoma: observational study with quantitative PCR. *Lancet.* 2003;362:198-204.
87. Jaschek G, Gaydos CA, Welsh LE, Quinn TC. Direct detection of Chlamydia trachomatis in urine specimens from symptomatic and asymptomatic men by using a rapid polymerase chain reaction assay. *Journal of clinical microbiology.* 1993;31:1209-1212.
88. Ostergaard L, Birkelund S, Christiansen G. Use of polymerase chain reaction for detection of Chlamydia trachomatis. *Journal of clinical microbiology.* 1990;28:1254-1260.
89. Bass CA, Jungkind DL, Silverman NS, Bondi JM. Clinical evaluation of a new polymerase chain reaction assay for detection of Chlamydia trachomatis in endocervical specimens. *J Clin Microbiol.* Oct 1993;31(10):2648-2653.
90. Boyadzhyan B, Yashina T, Yatabe JH, Patnaik M, Hill CS. Comparison of the APTIMA CT and GC assays with the APTIMA combo 2 assay, the Abbott LCx assay, and direct fluorescent-antibody and culture assays for detection of Chlamydia trachomatis and Neisseria gonorrhoeae. *J Clin Microbiol.* Jul 2004;42(7):3089-3093.
91. Chernesky M, Jang D, Portillo E, Chong S, Smieja M, Luinstra K, Petrich A, Macritchie C, Ewert R, Hayhoe B, Sarabia A, Thompson F. Abilities of APTIMA, AMPLICOR, and ProbeTec assays to detect Chlamydia trachomatis and Neisseria gonorrhoeae in PreservCyt ThinPrep Liquid-based Pap samples. *Journal of clinical microbiology.* 2007;45:2355-2358.
92. Yang J, Schachter J, Moncada J, Habte D, Zerihun M, House JI, Zhou Z, Hong KC, Maxey K, Gaynor BD, Lietman TM. Comparison of an rRNA-based and DNA-based nucleic acid amplification test for the detection of Chlamydia trachomatis in trachoma. *British Journal of Ophthalmology.* 2007;91:293-295.
93. MacCallan A. Ophthalmic conditions in the government schools and their amelioration *Ophthalmoscope.* 1908;vi:856-863, 947-952.
94. MacCallan A. The epidemiology of trachoma. . *British Journal of Ophthalmology* 1931;15:369-411.
95. Expert committee on trachoma: third report. . *World Health Organization Technical Report Series* 1962;234:1-48.
96. Fourth WHO scientific group on trachoma research. *World Health Organization Technical Report Series* 1966;330:1-24.
97. Darougar S, Jones BR. Trachoma. *British medical bulletin.* 1983;39:117-122.
98. Dawson C, Jones BR, Tarizzo ML. *Guide to trachoma control : in programmes for the prevention of blindness*. Geneva: World Health Organization; 1981.
99. Melese M, Alemayehu W, Bejiga A, Adamu Y, Worku A. Modified grading system for upper eyelid trachomatous trichiasis. *Ophthalmic epidemiology.* 2003;10:75-80.
100. Thylefors B, Dawson CR, Jones BR, West SK, Taylor HR. A simple system for the assessment of trachoma and its complications. *Bulletin of the World Health Organization.* 1987;65(4):477-483.
101. Taylor H, West SK, Katala S, Foster A. Trachoma: evaluation of a new grading scheme in the United Republic of Tanzania. *Bulletin of the World Health Organization.* 1987;65:485-488.
102. Wright H, Taylor HR. Clinical examination and laboratory tests for estimation of trachoma prevalence in a remote setting: what are they really telling us? *Lancet Infect Dis.* 2005;5:313-320.
103. Baral K, Osaki S, Shreshta B, Panta CR, Boulter A, Pang F, Cevallos V, Schachter J, Lietman T. Reliability of clinical diagnosis in identifying infectious trachoma in a low-prevalence area of Nepal. *Bull World Health Organ.* 1999;77:461-466.
104. Bird M, Dawson CR, Schachter JS, Miao Y, Shama A, Osman A, Bassem A, Lietman TM. Does the diagnosis of trachoma adequately identify ocular chlamydial infection in trachoma-endemic areas? *J Infect Dis.* 2003;187:1669-1673.
105. Thein J, Zhao P, Liu H, Xu J, Jha H, Miao Y, Pizzarello L, Tapert L, Schachter J, Mabon M, Osaki-Holm S, Lietman T, Paxton A. Does clinical diagnosis indicate ocular chlamydial infection in areas with a low prevalence of trachoma? *Ophthalmic Epidemiol.* 2002;9:263-269.

106. Bailey R, Hayes L, Pickett M, Whittle HC, Ward ME, Mabey DC. Molecular epidemiology of trachoma in a Gambian village. *Br J Ophthalmol.* 1994;78:813-817.
107. Miller K, Schmidt G, Melese M, Alemayehu W, Yi E, Cevallos V, Donnellan C, Olinger L, Fantaye D, Gaynor B, Whitcher JP, Lietman T. How reliable is the clinical exam in detecting ocular chlamydial infection? *Ophthalmic Epidemiol.* 2004;11:255-262.
108. Bobo L, Novak N, Muñoz B, Hsieh YH, Quinn TC, West S. Severe disease in children with trachoma is associated with persistent Chlamydia trachomatis infection. *J Infect Dis.* 1997;176:1524-1530.
109. Bird M, Dawson CR, Schachter JS, et al. Does the diagnosis of trachoma adequately identify ocular chlamydial infection in trachoma-endemic areas? *The Journal of infectious diseases.* May 15 2003;187(10):1669-1673.
110. Bobo LD, Novak N, Munoz B, Hsieh YH, Quinn TC, West S. Severe disease in children with trachoma is associated with persistent Chlamydia trachomatis infection. *The Journal of infectious diseases.* Dec 1997;176(6):1524-1530.
111. Murray D. Planning the analysis. *Design and analysis of group-randomized trials*. New York: Oxford University Press; 1998:77-130.
112. Eisenberg J, Lewis BL, Porco TC, Hubbard AH, Colford JM Jr. Bias due to secondary transmission in estimation of attributable risk from intervention trials. *Epidemiology.* 2003;14:442-450.
113. World_Bank. Appendix B. The global burden of disease, 1990. *World Development Report 1993: investing in health*. Oxford: Oxford University Press; 1993:213-225.
114. Murray C, Lopez AD. The global burden of disease in 1990: final results and their sensitivity to alternative epidemiological perspectives, discount rates, age-weights, and disability weights. In: Murray C, Lopez AD, ed. *The Global Burden of Disease*. Cambridge, MA: Harvard University Press; 1996:247-293.
115. Ranson M, Evans TG. The global burden of trachomatous visual impairment: I. Assessing prevalence. *Int Opthalmol.* 1995-1996;19:261-270.
116. Evans T, Ranson MK. The global burden of trachomatous visual impairment: II. Assessing burden. *Int Opthalmol.* 1995-1996;19:271-280.
117. Frick K, Basilion EV, Hanson CL, Colchero MA. Estimating the burden and economic impact of trachomatous visual loss. *Ophthalmic epidemiology.* 2003;10:121-132.
118. Cook JFK, Baltussen R, Resnikoff S, Smith A, Mecaskey J, and Kilima P. Loss of vision and hearing. In: Jamison D, Breman JG, Measham AR, Alleyne G, Claeson M, Evans DB, Jha P, Mills A, Musgrove P, ed. *Disease control priorities in developing countries (2nd edition)*. New York: Oxford University Press; 2006.
119. Frick K, West SK. The SAFE strategy for trachoma control: planning a cost-effectiveness analysis of the antibiotic component and beyond. *Ophthalmic epidemiology.* 2001;8:205-214.
120. Frick K, Hanson CL, Jacobson GA. Global burden of trachoma and economics of disease. *The American journal of tropical medicine and hygiene.* 2003;69:1-10.
121. Frick K, MeCaskey JW. Resource allocation to prevent trachomatous low vision among older individuals in rural areas of less developed countries. *Doc Ophthalmol.* 2002;105:1-21.
122. Frick K, Keuffel EL, Bowman RJ. Epidemiological, demographic, and economic analyses: measurement of the value of trichiasis surgery in The Gambia. *Ophthalmic epidemiology.* 2001;8:191-201.
123. Baltussen R, Sylla M, Frick KD, Mariotti SP. Cost-effectiveness of trachoma control in seven world regions. *Ophthalmic Epidemiol.* 2005;12:91-101.
124. Frick K, Lietman TM, Holm SO, Jha HC, Chaudhary JS, Bhatta RC. Cost-effectiveness of trachoma control measures: comparing targeted household treatment and mass treatment of children. *Bulletin of the World Health Organization.* 2001;79:201-207.
125. Murray C. Rethinking DALYs. In: Murray C, Lopez AD, ed. *The Global Burden of Disease*. Cambridge: Harvard University Press; 1996:1-89.
126. World Health Organization. Disability weights, discounting and age weighting of DALYs. 2010; http://www.who.int/healthinfo/global_burden_disease/daly_disability_weight/en/index.html. Accessed April 19th, 2010, 2010.
127. Fry AM, Jha HC, Lietman T, et al. Secondary effects of mass chemoprophylaxis with azithromycin to eliminate blindness due to trachoma in Nepal: Adverse and beneficial effects. *Clinical Infectious Diseases.* 2002;35(4):395-402.

TIRET

Statistical Analysis Plan

Confidential

Version 1.02, 10 September 2012

Prepared by:

Travis C. Porco, PhD, MPH
Division of Preventive Medicine and Public Health
Department of Epidemiology and Biostatistics
Box 0412
513 Parnassus Avenue Room S351
University of California, San Francisco
San Francisco, California 94143-0412
Telephone: (510) 323-3143
Email: travis.porco@ucsf.edu

1 Introduction

This document (Statistical Analysis Plan, SAP) describes the planned analysis and reporting for the **Trachoma Amelioration in Northern Amhara (TANA) II** study, University of California, San Francisco. It includes specifications for the statistical analyses and tables to be prepared for the final Clinical Study Report.

The proposed study is a Phase IV clinical trial to compare methods to reduce the level of ocular chlamydial infection using mass administration of azithromycin (Pfizer, CAS 83905-01-5), continuing the existing Trachoma Amelioration in Northern Amhara (TANA I) trial. Trachoma, resulting from repeated infection by ocular strains of *Chlamydia trachomatis*, is the world's leading infectious cause of blindness.

The content of this Statistical Analysis Plan meets the requirements stated by the US Food and Drug Administration and conforms to the American Statistical Association's Ethical Guidelines.

The following documents were reviewed in preparation of this Statistical Analysis Plan:

- Trachoma Amelioration in Northern Amhara (TANA) II, Manual of Operations
- ICH Guidance on Statistical Principles for Clinical Trials
- Statistical Analysis Plan, Trachoma Amelioration in Northern Amhara, (T. Lietman, principal investigator)

The planned analyses described in this Statistical Analysis Plan will be included in future manuscripts. Note, however, that exploratory analyses not necessarily identified in this Statistical Analysis Plan may be performed to support the analysis. All post-hoc or unplanned analyses which have not been delineated in this Statistical Analysis Plan will be clearly documented as such in the final Clinical Study Report, manuscripts, or any other document or submission.

Finalization of this document will take place prior to the enrollment of patients; the final version of this document will be numbered 2.0. All subsequent changes will be indicated by detailed change log in an Appendix.

B. Gaynor, J. Keenan, T. Lietman, and N. Stoller have contributed to this plan.

2 Investigational Plan

2.1 Introduction to the Study Design

The proposed TANA II study is an international, group-randomized, clinical trial to assess strategies to eliminate infection with *C. trachomatis*. Communities (referred to as *state teams* by the Ethiopian Government) will be randomized to one of six treatment strategies, and followed longitudinally. These communities will be randomized from the pool of communities which were studied in the previous study, TANA I (Trachoma Amelioration in Northern Amhara).

2.2 The first TANA Study (TANA I)

The TANA I study evaluated four main questions:

1. Is **biannual** mass administration of single-dose oral azithromycin to all members of a community more effective in eliminating infection due to *C. trachomatis* than **annual** mass administration of single-dose oral azithromycin to all members of a community?
2. Can targeted treatment limited to children-only yield significant reductions of infection among untreated adults in the same community?
3. Does installation and promotion of latrine usage reduce the rate of *C. trachomatis* infection among children in communities that receive mass antibiotic treatments?
4. Does mass administration of oral azithromycin reduce mortality in children aged one to nine?

Communities were randomized into one of six study arms:

Specific Aim 1:

- Annual mass administration of single-dose azithromycin to all individuals; follow-up complete at 42 months
- Biannual mass administration of single-dose azithromycin to all individuals; follow-up complete at 42 months

Specific Aim 2

- Quarterly mass administration of single-dose azithromycin to children-only; follow-up complete at 12 months
- Mass administration delayed for one year (serves as a control group); follow-up complete at 12 months

Specific Aim 3

- Single mass administration of azithromycin only at baseline; follow-up complete at 24 months
- Single mass administration of azithromycin at baseline, followed by intensive construction of latrines and promotion of latrine usage; follow-up complete at 24 months

2.3 Study Objectives and Endpoints of TANA II

2.3.1 Primary Objectives

The new study will have three Specific Aims, using communities monitored in the Specific Aim I of TANA I:

I) Can we stop antibiotics after 3 years? *We hypothesize that infection will return, even from low levels. 24 communities which received repeated mass treatments for four years will be monitored for 3 additional years.*

II) Can infection be completely eliminated if mass treatments continue for 6 years? *We hypothesize that infection will be completely eliminated in all communities. We will monitor the prevalence of infection in 12 communities which continue to receive annual mass treatments, and 12 which continue to receive biannual mass treatments.*

III) Can treatment targeted to cases and their contacts prevent infection from returning into the community? *We hypothesize that identifying cases clinically and treating these cases will delay or even prevent reemergence at a far lower cost than continued mass treatment of all individuals. We will monitor 12 communities where treatment is targeted to clinically active cases and their households, and another 12 communities where treatment is targeted to pre-school children.*

Monitored timepoints include 0, 12, 24, and 36 months after the beginning of TANA II (starting approximately 4 years after the beginning of TANA I). The prevalence of ocular chlamydial infection will be estimated by choosing 60 children aged 0-9 years old at random from the census of each community (anticipating that ≥50 children will be monitored), and conducting pooled PCR estimation of the prevalence, as described in the MOP. Note also that we will sample 60 individuals 10 years and older (for secondary outcomes). Individuals will NOT be monitored longitudinally throughout the trial; this is in part because during the course of the 3 year trial, individual children will age in and out of the years associated with the highest prevalence of infection (typically 3-5 year-olds). A new sample of children at each scheduled visit will serve to estimate the prevalence in that community and maintain a consistent age range in the sample. Thus communities will be followed longitudinally, but individuals in the community will not (as in TANA I).

2.4 Study Population and Communities

For this proposal, we will enroll communities which had previously been part of the Annual and Biannual (twice-yearly) study arms in TANA I. These communities will be re-randomized into one of six new study arms. These arms (with mnemonic abbreviations) are:

Specific Aim I:

- **Stop-Annual:** Communities from arm A (annual) from the current TANA I study, randomized to cessation of mass administration of azithromycin
- **Stop-Biannual:** Communities from arm B (biannual, twice-yearly) from the current TANA I study, randomized to cessation of mass administration of azithromycin

Specific Aim II

- **Continue-Annual:** Communities from arm A (annual) from the current TANA I study, randomized to continuation of annual mass administration of azithromycin
- **Continue-Biannual:** Communities from arm B (biannual) from the current TANA I study, randomized to continuation of *biannual* mass administration of azithromycin

Specific Aim III

- **Target-Exam**: Communities from arm A (annual) from the current TANA I study, randomized to a new strategy in which mass administration of azithromycin is discontinued, but field teams continue to visit the communities annually and treat all members of a household whenever any preschool (age 0–5) child in a household has clinical signs of trachomatous infection
- **Target-Age**: Communities from arm A (annual) from the current TANA I study, randomized to a new strategy in which mass administration of azithromycin is continued, but only for preschool (age 0–5) children rather than all members of the community

Specific Aim I is to be addressed by comparing baseline infection rates in 0-9 year-old children (month 48 after the TANA I baseline treatment) in communities in arms **Stop-Annual and Stop-Biannual** with rates at 36 months (month 84 after the TANA I baseline treatment), using a paired-sample test (see below for details).

Specific Aim II is to be addressed by comparing 36-month infection rates in children in communities in arms **Continue-Annual** with 36-month infection rates in children in communities in **Continue-Binnual** (see below for details).

Specific Aim III is to be addressed by comparing 36-month infection rates in children in arms **Target-Exam** and **Target-Age** (see below for details).

The drug resistance specific aim will determine (i) whether there is evidence that continuing to treat biannually results in more macrolide resistance to pneumococcus than does continuing to treat annually, (ii) whether targeted azithromycin treatments result in less macrolide resistance than mass treatments of the entire community, and (iii) whether or not evidence continues to support the idea that macrolide resistance in pneumococcus rapidly drops after cessation of mass administration of azithromycin (as we found in earlier studies).

The mortality specific aim will determine whether or not we continue to find evidence that mass administration of azithromycin reduces mortality in children.

Note that the following arm designations are used in internal TANA/TIRET documentation: Stop-Annual: Arm J; Stop-Biannual: Arm K; Continue-Annual: Arm L; Continue-Biannual: Arm M; Target-Age: Arm N; Target-Exam: Arm O.

2.4.1 Secondary Objectives

Analysis plans for all pre-specified secondary objectives will be presented during the first DSMC meeting. These include descriptive statistics, as well as comparisons between different study arms; note that since communities are drawn from the same pool of communities (annually or biannually treated as appropriate) that secondary comparisons are natural to perform. Secondary objectives will include:

Descriptive statistics for each of the 6 TANA II Arms, including prevalence of infection in children 0-9 years, prevalence of infection in individuals ≥10 years, prevalence of clinical activity (TF and/or TI) in children 0-9 years, and antibiotic coverage at each scheduled treatment.

For Specific Aim I (analysis of discontinuation of treatment), secondary analyses will include comparison of clinical activity in children at the beginning of TANA II and 36 months later (similar to the primary analysis of infection in children); comparison of infection in individuals aged ≥10 years; and correlation between clinical activity and infection in children at the community level at 36 months to assess whether clinical activity is an acceptable marker for identifying communities with the most infection post treatment.

For Specific Aim II (analysis of continuation of treatment), a secondary analysis will determine the proportion of communities which have achieved complete elimination in annually and biannually treated communities (**Continue-Annual** and **Continue-Biannual**). We will assess clinical activity in children at 36 months. The correlation between clinical activity and infection in children at the community level at 36 months will be used to assess whether clinical activity is an acceptable marker for identifying communities which have achieved elimination. A possible 42 month data collection will be discussed at the first DSMC meeting, and any revisions to the outcomes implied or indicated by this will be discussed at that time.

For Specific Aim III, several secondary comparisons will be considered: (a) comparison of **Target-Exam** with **Continue-Annual** (is targeting households as good as mass administration?), (b) comparison of **Target-Exam** with **Stop-Annual** (how does targeting households compare to simple cessation of mass administration?) (c) comparison of **Target-Age** with **Continue-Annual** (is targeting preschoolers as good as mass administration?), and finally (d) comparison of **Target-Age** with **Stop-Annual** (is targeting preschoolers better than complete cessation of treatment?) The mean prevalence of infection in individuals ≥10 years will be compared between **Stop-Annual** and **Target-Age**; this will reveal any degree of herd protection offered to adults by preventing transmission in pre-school children (House et al., *Lancet* 2009). Clinical activity in children 0-9 years at 36 months will be compared between **Target-Exam** and **Target-Age**.

An additional primary specific aim concerns comparison of mortality. Additionally, we propose to analyze anthropometric measurements, passive surveillance reports, and malaria. Details are provided below.

3 Study Methods

3.1 Overall Design

See Manual of Operations.

3.2 Study Population

See Manual of Operations.

3.3 Randomization

Communities originally from the annual arm of TANA I will be randomly allocated to **Stop-Annual**, **Continue-Annual**, **Target-Exam**, and **Target-Age**. Communities originally from the biannual arm of TANA I will be randomly allocated to **Stop-Biannual** and **Continue-Biannual**. In TANA I, 50 state teams (from 12 subkebeles) received annual treatment, and 61 state teams (from 12 subkebeles) received biannual treatment. Consideration has been given whether to make the TANA II randomization a simple random sample of the appropriate group of TANA I state teams, or to choose a single random state team from each of TANA I's randomized subkebeles.. Note that choosing one state team from each TANA I subkebele means that the primary Specific Aim III analysis will be between matched communities (i.e. each Target-Exam state team will be matched with a Target-Age state team from the same TANA I subkebele). Specific Aim I will compare 0 and 36 month prevalence in the same community (matched), and Specific Aim II will compare prevalence at 36 months in **Continue-Annual** versus **Continue-Biannual** (unmatched).

Randomization in a cluster-randomized trial at the community level is sometimes conducted in a manner that not only allocates communities to each arm with equal probability, but which is transparently random to all involved. A list of the state teams, by subkebele, from both the annual arm and the biannual arm of TANA I will be prepared. One state team from each annual subkebele will be randomly assigned to **Stop-Annual**, **Continue-Annual**, **Target-Exam**, and **Target-Age**. Any remaining communities will not be allocated to any arm. Similarly, for the biannual arm of the previous TANA I trial, a list of all state teams will be prepared by subkebele, and a single state team from each of the 12 subkebele will randomly be chosen and assigned to arms **Stop-Biannual** and **Continue-Biannual**.

Because there were not at least 4 state teams per subkebele in arm A of TANA I, we will randomly choose a state team from each subkebele to the greatest extent possible. After this has been completed, some subkebeles (with only 3 state teams) will not have an assignment to all 4 arms. Other subkebeles (with more than 4 state teams) will have unselected state teams, from which the remaining assignments will be made.

3.4 Masking

Field collection teams are masked to the treatment status of the communities they collect from. Laboratory personnel are masked to the treatment status (arm) of all samples. The subject of masking is discussed in further detail in the Manual of Operations.

4 Planned Analyses

4.1 Interim Analysis

No formal interim analysis is planned.

4.1.1 Stopping rules

Stopping rules for benefit, harm, and futility will be discussed with the DSMC (see Section 6.2) and would require explicit declaration of an interim analysis plan. We anticipate that, as with TANA I, the DSMC will be given authority to stop some or all Arms of the study due to unanticipated adverse events. We also anticipate that, as with TANA I, the DSMC will be given authority to authorize unscheduled mass treatments to communities in Specific Aims 1 or 3, when the mean prevalence in the arm exceeds 15% in children 0-9 years of age (see MOP, 3.5.1). We currently do not plan statistical efficacy stopping rules. We do not anticipate early stopping rules for efficacy, although this will be discussed with the DSMC prior to the initiation of the trial.

4.2 Final Analyses

All final, planned analyses identified in this Statistical Analysis Plan will be performed only when the final data are available.

5 Statistical methods

5.1 Sample size planning

The sample size was determined based on the results of previous studies (TEF and TANA I). For definiteness, the prevalence after one year of treatment of children in the childhood-treatment arm of TANA I revealed a standard deviation of 0.043 (House et al., *Lancet* 2009). For conservatism, we will assume the standard deviation is 0.05 in the outcome.

5.1.1 Trachoma Specific Aim 1

In Aim 1, we wish to determine whether or not infection will return by three years after discontinuation. The null hypothesis is that infection rates will be stable over this period of time. We assumed a standard deviation determined by the twelve-month PCR results for the prevalence in children in arms A and B *aggregated together (to preserve masking)*; twelve communities will provide approximately 80% power to detect a difference of 5% in the prevalence of trachoma (assuming a paired T-test for sample-size planning). Twelve communities will be randomized into each of arms **Stop-Annual** and **Stop-Biannual**.

5.1.2 Trachoma Specific Aim 2

In Aim 2, we wish to compare annual and biannual treatment at the time 36 months after the beginning of the new TANA II study. The outcome variable is the prevalence within each community as estimated by the RNA-based, pooled sampling from the entire communty. We assumed an effect size of 6% difference in the prevalence. Assuming a standard deviation of 0.05, standard formulas (Friedman et al, 1998) reveal that twelve communities provide at least 80% power (two-sided alpha, unpaired T-test assumed for sample size planning). Twelve communities will be randomized to each arm: **Continue-Annual** and **Continue-Biannual**.

5.1.3 Trachoma Specific Aim 3

In Aim 3, we wish to compare treatment of entire households when a child aged 6 months to 5 years is found to be clinically active to the policy of simply treating all children aged 6 months to 5 years (and no household members). As in Aim 2, we assumed an effect size of 6% difference in the prevalence. Assuming a standard deviation of 0.05, standard formulas (Friedman et al., 1998) reveal that twelve communities provide at least 80% power (two-sided alpha, unpaired T-test assumed for sample size planning). Twelve communities will be randomized to each arm: **Target-Exam** and **Target-Age**. Notice that in practice this will be a paired analysis, due to the fact that one state team from each arm will be selected from each of the 12 annually-treated subkebeles from TANA I.

5.1.4 Macrolide Resistance Primary Aim

Based on a standard deviation of approximately 0.15 (derived from the previous TEF study) in the prevalence of macrolide resistant pneumococcus, we estimate that twelve villages in each group will provide approximately 80% power to detect a difference (in absolute terms) of 20% in the prevalence of macrolide resistance, with a two-sided alpha of 0.05 (using the standard formula for the sample size of a two-group T-test, cited from Chow et al, 2003), conservatively inflating the final sample size by approximately 15% because of the use of the nonparametric Wilcoxon rank-sum statistic instead of the T-test (see Lehman, 2006).

5.1.5 Mortality Primary Aim

Assuming a total of 40 children aged 0.5-5 in each state team would give conservatively 2700 person-years of follow-up in each of two groups to be compared. We assumed a mortality difference of 50% between the two groups and estimate that we have somewhat over 80% power to detect such an effect, using negative binomial (or Poisson) regression. See Appendix for details.

5.1.6 Loss to follow-up

We expect no loss of communities based on prior studies; in the event of any loss to follow-up at the community level, we will use last observation carried forward and conduct the statistical analysis as indicated using these values. In the event of missing baseline values, missing prevalences will be computed using imputation from the mean of other communities as follows. For any missing baseline value from any of arms **Stop-Annual**, **Continue-Annual**, **Target-**

Exam, and **Target-Age**, the mean will be imputed using the mean of the baseline prevalence taken over all these arms (since they all will have been treated identically up to that point and no programmatic differences related to TANA II will have been undertaken). For any missing value from any of arms **Stop-Biannual** and **Continue-Biannual**, the mean will be imputed using the mean of the baseline prevalence taken over these two new arms (since, as for arms taken from TANA I annual, these communities all will have been treated identically up to that point and no programmatic differences related to TANA II will have been undertaken yet).

5.2 Analysis Populations

The primary analysis is at the community level (prevalence among communities in each arm). We will, however, consider the following analytic populations:

- The **screening population**, which is to include all subjects who provide (a) baseline screening (including any demographic, visual acuity, or photographic data), and (b) informed consent.
- The **safety/coverage population**, which is to include all patients who receive any amount of planned study medication (azithromycin or tetracycline). Coverage will be summarized by age for each community.

Major protocol deviations will be analyzed at the community level. The following deviations are possible:

- scheduled mass administration did not take place
- mass administration of antibiotic took place at the wrong time
- administration of antibiotic occurred outside the intended group
- scheduled sample collection takes place outside the prescribed window
- insufficiently many children are available for sampling
- coverage of the target group is too low

5.3 Data Collection and Quality Assurance

5.3.1 Quality assurance and security

Data collection forms, training, security, and quality assurance are discussed in the Manual of Operations.

5.3.2 Analysis sets

Data sets for analysis will be produced at the Proctor central site by database manager ZZ. Each will be a Microsoft Excel® worksheet containing a single header line whose variable names match the Access database. Each analysis set will be in the form of a rectangular table in which each column corresponds to a single variable and each row to an observation. All missing values will be coded explicitly using the string `NA` (as used in the R software). Codes for categorical

variables (such as 1 for male, and 2 for female) will be avoided in favor of self-documenting character strings (such as Male, Female) whenever possible. Automated checks will be made to ensure consistency and that each variable in the analysis set has in-range values (protecting against negative ages, spelling errors in categorical factors, and similar errors).

A detailed codebook will be prepared, containing for each variable, (a) the form from which the variable derived, (b) the text of the question (when relevant), (c) all possible values for the variable, and (d) summary statistics for the variable. Note that all codes and character strings that represent categorical factors will be clearly defined in the codebook. Units for each continuous variable (e.g. mm vs. mm^2, logMAR, etc.) will be unambiguously indicated for each variable. Each release of the analysis set will be accompanied by the corresponding version of the codebook. Version numbering with dates will be strictly observed. Standard report-generation software included with the R statistical and data analysis package will be used to ensure consistency of the codebook and analysis set at all times.

5.3.3 Data monitoring reports

Data monitoring reports will be prepared based on analysis data sets. These will be prepared using report-generation software. Monitoring reports will include (a) baseline and follow-up census reports for each community, (b) antibiotic distribution and clinical activity reports, and (d) data quality reports. These will be reviewed at the central site, and communicated to the field teams.

5.4 Baseline characteristics

5.4.1 Demographics and Patient History

All demographic and history variables (in particular, age, gender, occupation, and national origin) determined at presentation or enrollment will be summarized by counts and percentages tabulated by community.

Age-specific breakdowns will be available for each community from the baseline census. Additional baseline characteristics will be recorded for each community: altitude as determined by GPS, distance to the nearest main road, distance to the regional administrative center (Gandewayn).

Detailed records of all government health activities (education, vaccination, or treatment programs) in the region will be collected.

5.4.2 Prior and concurrent medication

We will present the percentage receiving study medication by age, gender, and community. For both arms **Target-Exam** and **Target-Age**, we will record the number of preschool children and the number of households with a member showing clinical activity, so that strictly comparable data will be available for each.

5.4.3 Compliance and coverage

Detailed coverage reports for each visit (broken down by age, gender, and target group, e.g. preschool children, household members) will be presented for each visit, and for each antibiotic (a small number of individuals receive tetracycline ointment).

5.5 Hypothesis Tests

5.5.1 General considerations

Final agreement on test statistics will be made at the first meeting of the Data and Safety Monitoring Committee prior to the collection of any data.

All primary hypothesis tests will be conducted with an alpha of 0.05. Note that the subjects for the primary trachoma-related study questions for each of the 3 Specific Aims are completely independent, and these are viewed as independent clinical trials. Additionally, we consider the mortality and drug-resistance aims to be separate primary analyses, each with a separate hypothesis to be conducted at an alpha of 0.05.

The study hypotheses are listed as bidirectional effects; we will never conduct one sided tests.

Effect sizes will be reported along with all hypothesis tests, together with corresponding confidence intervals. As emphasized earlier, no interim analysis is planned.

Because the legitimacy of the hypothesis tests being conducted depends on the assumptions (i.e. normality and homoskedasticity for linear models) the adequacy of the statistical model must be checked. Methods which will be employed may include (a) residual plots (vs. baseline value, vs. predicted values, and Q-Q plots), (b) jackknife influence estimates, and (c) when appropriate, tests for normality (including the Anderson-Darling and Shapiro-Wilk procedures).

All analyses are conducted at the community level, thereby accounting for the group-randomization. When comparing between two arms, we square-root transformed the prevalence. When analyzing the change in prevalence longitudinally, we analyzed the prevalence itself. We included TIRET-baseline prevalence as a covariate. When comparing communities that had been randomized to different treatments pre-TIRET (in TANA), we did not include TIRET-baseline as a covariate as this would be a post-randomization correction.

Unless stated otherwise, *children* will always refer to individuals between the ages of 0 and 9, including individuals aged nine years old up to the day prior to their tenth birthday; *adult* will refer to individuals from the date of their tenth birthday and older. Pre-school children will refer to children between the ages of 0 and 5. Slightly different study populations are used for mortality assessments (age 0.5 to 5), due to the fact that oral azithromycin is not given in this study to those less than 6 months of age.

If arms are discontinued early (for instance, because the average prevalence exceeded the treatment threshold of 15%), the prescribed analysis will be conducted with the available data (an appropriate adjustment for alpha will be made, if necessary).

5.5.2 Prespecified comparisons for each primary aim

Trachoma Specific Aim 1

For Aim 1, we propose to conduct a single paired Wilcoxon test for all communities aggregated together, whether from **Stop-Annual** or from **Stop-Biannual**. This will be the primary analysis. In addition, prespecified analyses will also examine each arm **Stop-Annual** and **Stop-Biannual** separately, and together using ANOVA (including whether or not the community had come from the **Stop-Annual** or the **Stop-Biannual** prior to cessation of mass administration).

Residual plots (versus baseline value, versus predicted values and a Q-Q plot) will be investigated for outliers, nonlinearity, non-homogeneous variances and gross violations of normality.

Additional secondary analyses are discussed below.

Trachoma Specific Aim 2

For Aim 2, we propose to conduct a linear model (ANCOVA) comparing arms **Continue-Annual** and **Continue-Biannual** at 36 months, including (1) treatment arm (annual or biannual) and (2) the prevalence value at the beginning of TANA II as the two covariates. Specifically, the outcome variable is the 36-month prevalence of infection among children, in each community. The test statistic is the Huber regression coefficient corresponding to the study arm. Significance will be assessed by Monte Carlo permutation testing with at least 10000 replicates.

Residual plots (versus baseline value, versus predicted values and a Q-Q plot) will be investigated for outliers, nonlinearity, non-homogeneous variances and gross violations of normality.

Note that it is possible that both arms will show zero prevalence by the end of the study, indicating that both methods are successful in eliminating infection according to our measure. We consider this, in itself, to be an interesting result. Since comparison of the final zero prevalences is not a meaningful one, in this case the primary analysis will compare the time to elimination (defined specifically as the time between the beginning of the TANA II study and the first estimated zero-prevalence visit) between the two arms using a Wilcoxon rank-sum test. Also note that no-identifiable-infection in any of the communities is itself a very interesting result. The analysis of time to elimination will be conducted regardless of whether or not the prevalence is zero.

In addition, a survival analysis of time to elimination will be performed to compare the two arms. Elimination will be defined using both children and adults.

Additional secondary analyses are discussed below.

Trachoma Specific Aim 3

For Aim 3, we propose to conduct a single ANCOVA comparing arms **Target-Exam** and **Target-Age** at 36 months, including (1) treatment arm (household targeting vs all preschool) and (2) the prevalence value for children at the beginning of TANA II as the two covariates. Specifically, the outcome variable is the 36-month prevalence of infection among children, in each community. The test statistic will be the estimated Huber regression coefficient for

treatment arm. Significance will be assessed using Monte Carlo permutation testing with at least 10000 replicates (matching will be accounted for).

As before, residual plots (versus baseline value, versus predicted values and a Q-Q plot) will be investigated for outliers, nonlinearity, non-homogeneous variances and gross violations of normality.

Note that as in Aim 2, it is possible that both arms will show zero prevalence by the end of the study, indicating that both methods are successful in completely eliminating infection according to our measure. In this case, the primary analysis will compare the time to elimination (defined specifically as the time between the beginning of the TANA II study and the first estimated zero-prevalence visit) between the two arms using a Wilcoxon rank-sum test.

Trachoma, Secondary analyses

Additional natural prespecified analyses regarding the final (36-month) prevalence in children from 2.4.1 above include:

1. Arm **Target-Exam** and **Continue-Annual**, i.e. treating symptomatic preschoolers and their households to continued mass administration for the whole community.
2. Arm **Target-Exam** and **Stop-Annual**, i.e. treating symptomatic preschoolers and their households to no further treatment.
3. Arm **Target-Age** and **Continue-Annual**, i.e. treating all preschoolers to continued mass administration for the whole community.
4. Arm **Target-Age** and **Stop-Annual**, i.e. treating all preschoolers to no further treatment.
5. Arm **Stop-Annual** and **Continue-Annual**
6. Arm **Stop-Biannual** and **Continue-Biannual**

Note also that infection in individuals 10-years and older will be compared, and clinical activity in individuals 0-9 years old will be compared. We emphasize the special importance of the following comparison (which will not be treated together with all the other comparisons of adults in a multiple comparisons sense): the comparison of individuals 10-years and older in **Stop-Annual** and **Target-Age** will assess whether adults are protected from re-infection by residing in communities where pre-school children continue to receive treatments, offering more evidence of herd protection in mass treatment campaigns (House et al., *Lancet* 2009).

Each of these comparisons will be conducted at the 0.01 level, two sided. The corresponding confidence intervals will be reported.

A time series analysis will also be conducted in which linear mixed effects regression will be applied to compare the data at multiple time points between different arms. We will also conduct pooled regressions.

Additional secondary analyses will be conducted using mathematical transmission modeling. Such analyses will be used to estimate the optimal stopping time of mass administration, the optimal restart policy, or to estimate transmission and recovery parameters for mass-action and related disease transmission models.

Additional geographic analyses will be conducted. Specifically, we will model spatiotemporal autocorrelation between changes in prevalence in a given village and its neighbors. Such analyses will be clearly labeled as exploratory and will be distinguished from primary or secondary prespecified comparisons.

Primary aim for drug resistance

We propose a Wilcoxon rank-sum test for several comparisons of nasopharyngeal pneumococcal macrolide resistance at 36 months, to be two-sided at an alpha of 0.05.

We plan to use the fraction of sampled children in whom resistant isolates were detected as the primary outcome, although the fraction of isolates which are resistant will also be assessed.

A baseline comparison of all annually treated state teams and all biannually treated state teams will be conducted. These state teams were randomized during the TANA I study and treated for four years, so this is a randomized comparison. The hypothesis test will be conducted using the Wilcoxon rank-sum test. The median prevalence of drug resistance in each arm will be reported, along with the means and the difference in the means.

As a primary prespecified comparison, we will conduct a one-way ANOVA comparing the prevalence at 36 months in children aged 0-9 between the Continue-annual, Target-exam, and Target-age arms. The square root or other transformation may be used in the event of failure of normality or homoskedasticity. Pairwise comparisons will be conducted using the Tukey HSD method in the event of an overall significant difference. The Kruskal-Wallis procedure will be used in the event transformations cannot achieve adequate normality or homoskedasticity.

As a secondary prespecified comparison, we will aggregate the Stop-annual arm and Stop-biannual arms, and record the difference between the baseline prevalence and the 36 month prevalence for each. Change will be assessed using the Wilcoxon signed rank test, to determine whether there is evidence that the prevalence of resistance drops. We will also report the confidence interval for the estimated change.

In general, for each of the prespecified trachoma comparisons we have discussed, we intend a corresponding comparison of macrolide resistance (this includes each primary prespecified comparison, and each of the six comparisons listed above). This comparison will be conducted at the same significance level as the corresponding trachoma comparison. Of particular interest are the comparisons between the stop arms and the continue arms (Stop-annual vs Continue-annual as a Wilcoxon rank-sum (unpaired) test, Stop-biannual vs Continue-biannual as a Wilcoxon rank-sum (unpaired) test, Stop-annual and Stop-biannual together vs Continue-annual and Continue-biannual together as an ANCOVA wherein baseline resistance is included as a covariate, items 5 and 6 above). Also, the comparison between annual and biannual is of interest, to determine whether or not more antibiotic is associated with more resistance (comparison of Continue-annual and Continue-biannual, controlling for prevalence at baseline); this will be conducted using both linear statistical models (including linear mixed models and pooled regression) and mathematical transmission models.

We will also perform genetic testing on all pneumococcal isolates, including for the most common genetic determinants of macrolide resistance (*ermB* and *mefA*), and multilocus sequence typing. We will also perform serotyping of all pneumococcal isolates. This information will be

used for several purposes: first, we will describe the proportion of resistance seen in each serotype/strain type, and test whether serotype or strain type is associated with resistance using a Fisher's exact test of an r x c contingency table. We will also test whether strain diversity is associated with the prevalence of resistance by computing the Simpson's index of diversity in each village, and performing linear regression with the log-transformed prevalence of resistance as the outcome variable, and Simpson's index as the predictor. Bootstrap resampling will be used in hypothesis testing. The number of bootstrap samples will be adaptively chosen to ensure adequate convergence of the estimated p-value.

Additional analyses will be conducted using mathematical transmission models of pneumococcus, as well as geographic variables (to determine whether prevalence of resistance in nearby villages affects a given village). Such analyses will be considered exploratory and will be clearly distinguished from primary or secondary prespecified analyses.

Primary aim for mortality

We propose negative binomial regression to model deaths in children aged 0.5-5 years (the estimated person-years serves as the offset). Specifically, we will combine the Stop-Annual and Stop-Biannual groups, and compare these with the Continue-Annual and Continue-Biannual groups, to see if there is a lower mortality rate in the groups that continued to receive azithromycin. This comparison will be conducted at an 0.05 level, two sided.

Additional mortality comparisons may be conducted according to the same prespecified comparisons as indicated for trachoma and macrolide resistance. Regression assumptions will be checked using goodness of fit tests, goodness of link tests, and residual plots. Sensitivity analyses will be conducted for missing data, and in addition, analysis using at least one different method (such as clustered logistic regression) will be conducted to ensure that our final result is not an artifact of the statistical method chosen. Such supplemental sensitivity analyses will always be reported as such and clearly distinguished from the primary prespecified analysis.

Additional sensitivity analyses in which village-level covariates are included in the negative-binomial regression will be conducted. Specific covariates include the fraction of female children (ages 0-9), the fraction of children (out of the whole population), altitude (in meters) of the state team, distance of the state team to the main road, and distance of the state team to the regional administrative center (Gandewayn).

A secondary analysis will be conducted using individual-level data to determine whether or not actually receiving the antibiotic was protective (reduced mortality). Clustered logistic regression at the village level and the household level (nested within village) will be used (provided the model converges). In the event of nonconvergence, the household random effect will be removed. This analysis differs from the primary prespecified analysis, in that communities, not individuals, were randomized, so that this secondary analysis is observational. The hypothesis test is that the regression coefficient for the indicator variable for whether or not a person received azithromycin was different from zero (the two-sided 95% Wald confidence interval for this coefficient excludes zero). Age will be included as the only other covariate.

Infectious mortality will be assessed by verbal autopsy (see MOP for details). We will compare the degree of infectious mortality between the Continue-Annual and Stop-Annual arms, and between the Continue-Biannual and Stop-Biannual arms.

The mortality assessment will include data on the timing of deaths (see MOP for details). Models will be developed to assess the relation between timing of mortality and the azithromycin mass treatments. These models will include age and (where possible) infectious cause of mortality.

Morbidity and anthropometry

Passive surveillance data will be analyzed as follows. The outcome variable is the number of clinic visits for infectious causes made by individuals from each village over the previous year. We propose to compare the two 'stop' arms (J/Stop Annual and K/Stop Biannual) to the two `continue' arms (L/Continue Annual and M/Continue Biannual). The null hypothesis is that there is no difference between the number of clinic visits in these two groups. The proposed test statistic is Poisson regression (computing the person time at risk from the number present at the beginning of the 12 months, minus one half of all individuals known to have moved permanently or died). Individuals who move from one location to another within the study area will be counted as contributing to the randomization unit to which they first belonged (we do not expect this to be a frequent occurrence). Additional analyses will include the fraction of individuals below the age of 10 as a covariate. Other covariates may be included in an exploratory manner and will be labeled as such.

A second analysis regarding **passive surveillance** will concern timing. Specifically, we propose to tabulate the total number of infectious illnesses in each village for the three months prior to a mass antibiotic distribution, and for the three months following. This will be done for all villages assigned to L/continue annual or to M/continue biannual, yielding a pair of counts for each village. We propose to compare the counts before and after the distribution using the Wilcoxon signed-rank test. The analysis will be repeated in the L/continue annual group separately, and in the M/continue biannual group separately.

School study. Clinically active trachoma (as determined by the WHO simplified grading system) will be compared, based on all children under age 10, selected from a school.

Malaria data will be compared as follows. We propose to compare malaria parasitemia (as a binary variable measured at the individual level) between K/stop biannual and M/continue biannual villages. The analysis will be conducted using clustered logistic regression (with village as a random effect). The final P-value will be derived from a permutation test of village assignments. Details on timing of measurements and target populations will be given in the MOP. Additional comparisons of hemoglobin in 0-9 year olds (individual level, linear mixed effects regression) and of gametocytemia will be conducted also.

Anthropometric analysis will be conducted. We propose to compare (a) height, (b) weight, (c) weight-for-height Z score, (d) height-for-age Z score, (e) weight-for-age Z score using clustered linear regression (community as a random effect). The primary analysis will concern height for children measured (see MOP for details), and will be two sided at alpha of 0.05; other analyses are secondary and will be reported as such. Note that we will compare arms K and M.

The primary statistical model will be conducted on heights measured at the two time points as joint outcomes; fixed effects will be community treatment assignment (K vs M), age, sex, and time (first or final); random effects will be individual (since two observations are made on each individual) and community. We also propose (1) to regress the change in height between the two time points, using assignment as a fixed effect, and clustering on village, and (2) to regress height on weight with arm and arm-weight interaction as covariates, again using clustered linear regression. Individuals who are missing due to mortality could introduce bias; if an individual is present at the first measurement but is absent at the second due to mortality, we propose (in a sensitivity analysis) to assign a final height equal to their first measurement of height.

Table 1. Summary of primary prespecified test statistics. Abbreviations: SA **Stop-Annual**, SB **Stop- Biannual**, CA **Continue-Annual**, CB **Continue-Biannual**, TE **Target-Exam**, TA **Target-Age**. Each of these will be conducted at the 0.05 level.

Aim	Method	Age group	Outcome	Test statistic	Assumptions unmet?	Purpose
Trachoma Aim 1	Paired T, transformed	Children 0-9 years	PCR prevalence, SA and SB, 36 months	T	Wilcoxon signed rank	Primary
Trachoma Aim 2	ANCOVA, transformed, arm and baseline as covariates	Children 0-9 years	PCR prevalence, CA vs CB, 36 month	T for arm	Rank regression	Primary
Trachoma Aim 3	ANCOVA, transformed, arm and baseline as covariates	Children 0-9 years	PCR prevalence, TE vs TA, 36 month	T for arm	Rank regression	Primary
Resistance	Unpaired T, transformed	Children 0-9 years	Prevalence of macrolide resistance, CA vs CB	T	Wilcoxon rank sum	Primary
Mortality	Negative binomial regression, person-time denominator	Children 0.5-5 years	Mortality rate, SA and SB vs CA and CB, at 36 months	Wald test for regression coefficient	Clustered logistic regression	Primary

5.6 Missing data and loss to follow-up

Missing values of the primary study endpoint (prevalence of infection in each community at time 36 months after the beginning) will be analyzed by last-observation carried forward. These results will be compared with the results of imputation based on clinical activity or previous observations.

Missing baseline covariates will be handled by imputation from the distribution at baseline of other villages with the same treatment history. We will use multiple imputation with 10 replicates; a random number seed will be prespecified prior to multiple imputation, and the multiply imputed covariates will be generated prior to the collection of any outcome data and used subsequently. Standard formulae will be used to combine estimates from multiple imputation to an overall hypothesis test (see Little and Rubin, 2002).

5.7 Multiple comparisons

An alpha of 0.05 will be used for each of the primary analyses of Specific Aims 1, 2, and 3, and for the mortality and resistance outcomes. For pre-specified secondary analyses, we will report both the *P*-value and the number of pre-specified analyses performed. For the six secondary analyses, the *P*-value will be set at 0.01.

5.8 Software

The standard software package R (`http://www.r-project.org`) for the MacIntosh OS X will be used for all descriptive and inferential analyses.

6 Human Subjects

6.1 Summary of final dispositions

All subjects who provide informed consent will be accounted for in this study. The frequency of subjects in each population will be presented. We will also present the frequency of subjects in each community, the community-specific coverage, and any major protocol violations.

6.2 Data and Safety Monitoring Committee

6.2.1 Scope

A Data and Safety Monitoring Committee (DSMC) will be empaneled by the NEI. We propose that this committee consist of 5-7 individuals, and should include (a) cornea specialists, (b) an independent biostatistician, (c) a bioethicist, and (d) representation from both Ethiopia and the United States. The committee will meet in person at least once per year, and will convene biannual teleconferences for progress reports. *Ad hoc* meetings as needed may also be convened. All study protocols will be subject to review and approval by Institutional Review Boards at the UCSF and the Ethiopian Science and Technology Commission, and by the DSMC.

The Data and Safety Monitoring Committee will informally review the interim efficacy data, and be empowered to make one of the following recommendations:

- Continue the trial without modifications
- Continue the trial with study modifications
- Terminate enrollment or treatment in the trial because of safety concerns
- Terminate enrollment or treatment in the trial because of efficacy
- Terminate enrollment or treatment in the trial because of futility

6.2.2 Meetings

All in-person and teleconference meetings of the DSMC and study personnel will consist of (a) "open" sessions, which may be attended as needed by masked study personnel, and (b) "closed" sessions, which may only be attended by unmasked study personnel (TP, KR). Masking is primarily with respect to laboratory findings, and care will be taken so that *no* laboratory findings or data which would allow laboratory findings to be determined will be revealed during the open sessions.

Interim reports for the DSMC will be prepared by the central Proctor site (TP). These reports will include (a) baseline or follow-up census reports by community, (b) treatment distribution and coverage, (c) clinical activity, and (d) safety monitoring and adverse outcomes. The DSMC will determine the database closure dates for each report in advance; archival copies of the (a) main SQL database, and (b) study analysis file as they exist at the time of each report will be maintained. All reports will be sent using secure email to the members of the DSMC two weeks prior to each meeting. Each interim report will be labeled clearly as confidential, printed in binding so that the contents are not visible from the outside, and labeled with the name of each person authorized to receive it. Reports will be kept in possession of KR and TP and only delivered in person; reports not delivered due to absences are to be shredded. In addition, redacted versions of the interim reports will be prepared which contain no masked study information, and which are suitable for restricted distribution to other personnel on an as-needed basis. All hard copies will be destroyed at the end of each meeting, except for a copy to be kept in a locked file cabinet accessible only to TP and KR.

6.2.3 Decisions

The DSMC will make decisions with the benefit of prespecified decision guidelines. These guidelines will be agreed upon at the initial meeting, and may include (a) safety, (b) efficacy, (c) clinical importance, (d) effect of baseline covariates, or (e) validity.

Stopping for harm will be done at the judgment of the DSMC. Adverse outcomes will be monitored by the DSMC. While the analysis would consider maldistribution of predictive factors such as age, it is recognized that ethical considerations require careful considerations of statistical tests as well as qualitative judgments in the light of experience. Any additional analyses required by the DSMC will be conducted by TP and KR, as needed.

Note that serious adverse events (SAE) are reported directly to the DSMC Chair within 24 hours of the time the study site learns of them. The DSMC Chair receives notification of the

event, the timing of the event, a medical narrative from the medical monitor, the site, the patient identification number. The statistician reports the study treatment assignment to the DSMC Chair. If azithromycin use clearly results in an unacceptable increase in serious adverse outcomes, then the study will be stopped. Considerable experience from many groups suggests that this outcome is highly unlikely. It is difficult to fully prescribe boundaries for monitoring safety because there need not be strong evidence to discontinue the study if it appears that the treatment is harmful.

7 Safety and tolerability

The analysis of safety in this study will include summaries of the following:

- Exposure
- Adverse events
    - Adverse events and serious adverse events (including perforations)
    - Adverse events leading to withdrawal
    - Any deaths

As for the TANA I study, an additional adverse events survey will be administered; see Manual of Operations for details. Community-specific estimates for the proportion of individuals (by age group) will be presented to the DSMC at each meeting.

7.1 Exposure

Individuals are assumed to have exposure to the drug that was provided (azithromycin or tetracycline).

7.2 Adverse Events

12.2.1 Individual events

The proportion of subjects with at least one of the following safety-related events will be compared using Fisher's Exact Test. Non-serious adverse events (not requiring narrative form) are

- Other events determined to be adverse by a site director

Serious adverse events (which must also be reported within 24 hours and which require a narrative form) are

- Death
- Non-elective surgery, hospitalization, or loss of function

12.2.2 Pooled adverse events

Adverse events will be analyzed according to five main categories:

- Proportion of subjects with *any serious adverse event*

The proportion of subjects with these events will be compared between the arms using Fisher's Exact Test. Poisson regression will be applied to compare the rates of overall adverse events, including recurrent events.

8 Reporting conventions

- All tables and data listings will be presented in landscape orientation, unless presented as part of the text of the final report.
- Figures will be presented in landscape orientation, unless the information is substantially easier to interpret in portrait orientation.
- Direct annotation of figures will be preferred to legends. All figures with more than one variable or item will contain either direct annotation or legends. All annotation will be unambiguously identifiable as such.
- Color will be used in figures only when needed to enhance clarity of communication. All color schemes will be evaluated for visual clarity for individuals with diminished color vision. All color encodings will be identified. Redundant encodings (such as the use of different plot symbols or line dash patterns) will be used in addition to color, so that all figures are interpretable after monochrome reproduction at 100 dots per inch. All dash patterns and line widths will be adequate to be distinguishable after monochrome reproduction at 100 dots per inch. Any distinction between plot symbols (circles, filled circles, diamonds, etc.) will remain clear after monochrome reproduction at 100 dots per inch.
- Fixed width sans serif fonts will be used for all labeling (Helvetica, Arial, or Futura).
- Boldface and italics will not be used unless substantial value is added.
- Decorative fonts and enhancements, including borders and shading, will not be used. Decorative presentation methods, such as ribbon graphs, will never be used.
- All information given in figures will also be presented in summary tables (perhaps only included in an Appendix or in supplementary materials).
- Only standard characters will be used in tables and data listings.
- All titles will be centered. The first title line will be the number of the table, figure, or listing. The second and possibly third lines will be the description of the table, figure, or data listing. The ICH numbering convention will be used for all.
- All footnotes will be left justified and at the page bottom. Footnotes will be used sparingly. Reference footnotes will be complete enough to locate any reference based on the information provided (Author, Journal, Pages, Date, or PubMed accession number).
- Missing values for numeric or character variables will be unambiguously identified as such using the special string `NA` (not available) in all settings; `NA` is the standard missing value code for our software. Each figure or table caption in which `NA` is used will indicate the meaning of `NA` in that figure or table. The abbreviation `NA` will never be used for any other purpose.
- All date values will presented in the form DDmmmYYYY format (e.g. 01jan2008), using four digit years. June will be encoded as `jne` (otherwise `jan` and `jun` would differ by

only a single character), and July as `jly` (so that the lowercase letter `l`, easily confused with the digit `1`, will not be adjacent to any numerals).

- All tables, figures, and data listings will have the name of the program and a date/time stamp on the bottom of the output.

9 Abbreviations and acronyms

AES Advanced Encryption Standard
ANCOVA Analysis of covariance
CA Continue-Annual study arm
CB Continue-Biannual study arm
CAS Chemical Abstracts Service
DSMC Data and Safety Monitoring Committee
FIPS Federal Information Processing Standard
KR K. Ray
NIST National Institute of Standards and Technology
SA Stop-Annual study arm
SB Stop-Biannual study arm
TA Target-Age study arm
TE Target-Exam study arm
TP T. Porco
UCSF University of California, San Francisco
ZZ Z. Zhou

10 References

American Statistical Association. 1999. *Ethical Guidelines for Statistical Practice.* Alexandria, Virginia: ASA.

Chow SC, Shao J, Wang H. Sample size calculations in clinical research. Boca Raton: CRC/Taylor and Francis.

Department of Health and Human Services, Food and Drug Administration. 1998. International Conference on Harmonization; Guidance on Statistical Principles for Clinical Trials. *US Federal Register* **63**(179):49583-49598. Docket 97D-0174.

Friedman LM, Furberg CD, DeMets DL. Fundamentals of Clinical Trials, 3d ed. New York: Springer Verlag, 1998.

House JI, Ayele B, Porco TC, Zhou Z, Hong KC, Gebre T, Ray KJ, Kennan JD, Stoller NE, Whitcher JP, Gaynor BD, Emerson PM, Lietman TM. Assessment of herd protection against trachoma due to repeated mass antibiotic distributions: a cluster-randomized trial. Lancet 373:1111-1118, 2009.

Lehmann EL. Nonparametrics: statistical methods based on ranks. New York: Springer-Verlag, 2006.

Little RJA, Rubin DB. Statistical Analysis with Missing Data. New York: John Wiley, 2002.

11 Appendix

To estimate the power for our negative binomial or Poisson regression for the study for mortality, we conducted a small simulation study. We assumed a baseline mortality rate of 0.02 per year for children aged 0.5-5, an effect size of 50% (halving the mortality rate), on average 40 children per group, 24 groups in each arm, a standard deviation of 5 in the number of children per group, three years of follow-up, and 2.5% loss per year due to moving. We also assumed that the mortality rates between villages had a coefficient of variation of 0.3 (thereby conservatively assuming considerable heterogeneity within this small group of similar villages).

Simulation revealed approximately 84% power to detect the 50% mortality difference. The simulation code (verified for R v 2.12 for MacIntosh) is provided below.

```
mkdata <- function(nvillages.per.group=24,
                   groups=c("A","B"),
                   mean.children.per.group=40,
                   sd.children.per.group=5,
                   studylength=3,
                   mean.mort.rates=c(0.02,0.01),
                   cv.mort.rates=0.3,
                   loss.rate = 0.025,
                   trace=FALSE,
                   verbose=TRUE) {
  cns <- as.character(1:nvillages.per.group)
  vnames <- paste(groups[1],cns,sep="")
  vg <- rep(groups[1],nvillages.per.group)
  ngroups <- length(groups)
  if (ngroups>=2) {
    for (ii in 2:ngroups) {
      vnames <- c(vnames,paste(groups[ii],cns,sep=""))
      vg <- c(vg,rep(groups[ii],nvillages.per.group))
    }
  }
  meanrates <- mean.mort.rates
  names(meanrates) <- groups
  nvs <- length(vnames)
  chbyg <- round(rnorm(
nvs,mean=mean.children.per.group,sd=sd.children.per.group))
  villages <- data.frame(village=vnames,arm=vg,nchildren=chbyg)
  village.mean.rates <- meanrates[vg]
  village.sds <- cv.mort.rates * village.mean.rates
  village.specific.means <-
rnorm(length(village.mean.rates),mean=village.mean.rates,sd=villa
ge.sds)
  aas <- village.mean.rates^2 / village.sds^2
  sss <- village.sds^2 / village.mean.rates
```

```
  village.specific.means <-
rgamma(length(village.mean.rates),shape=aas,scale=sss)
  villages$nmort <- NA
  villages$futime <- NA
  if (trace) {cat("mkdata: before mtimes loop\n")}
  for (ii in 1:nvs) {
    mtimes <-
rexp(villages$nchildren[ii],rate=village.specific.means[ii])
    ltimes <- rexp(villages$nchildren[ii],rate=loss.rate)
    futimes <- pmin(mtimes,ltimes)
    totfutime <- sum((futimes<studylength)*futimes +
(futimes>=studylength)*studylength)
    villages$futime[ii] <- totfutime
    conf.mort <- (mtimes<ltimes) & mtimes<=studylength
    villages$nmort[ii] <- sum(conf.mort)
  }
  villages
}

pwrsim <- function(nsims=1000) {
  ans <- rep(NA,nsims)
  for (ii in 1:nsims) {
    dtmp <- mkdata()
    res <- glm(model.frame(nmort ~ arm,
offset=log(dtmp$futime),data=dtmp),family=poisson)
    ress <- summary(res)
    ans[ii] <- ress$coefficients["armB",4]
  }
  ans
}
```

12 Changes

5 May 2011 Clarification that the secondary analyses will be conducted at a significance level of 0.01 (p. 12, 15, and 18).

5 May 2011 Correction of typographic error. The sixth prespecified secondary analysis is comparison of Stop-Biannual vs Continue-Biannual.

5 May 2011 Page 5. Addition of single-letter abbreviations for the six study arms.

5 May 2011 Page 17. Omission of multiple imputation for outcome variables (though not for baseline covariates) for primary analysis in favor of last observation carried forward.

8 September 2012. Page 17. Morbidity and anthropometry section.

9 September 2012. Sec. 5.5.2. Primary analysis changed from paired T-test to paired Wilcoxon (Wilcoxon signed rank test). This decision is made without the final data and is thus unaffected by the data.

9 September 2012. Sec. 5. Specific Aims 2 and 3. Significance testing will be conducted by permutation testing with at least 10,000 replicates. References to rank regression omitted. Huber robust estimation preferred.

10 September 2012. Page 12. Clarification that analysis will be conducted on last available result in case of discontinuation of study arms.

7 April 2017. Page 17. adjustment for age and sex were added to the Anthropometry primary analysis because they are highly correlated with height.

9 September 2017. Page 12. Added clarification under general considerations “When comparing between two arms, we square-root transformed the prevalence. When analyzing the change in prevalence longitudinally, we analyzed the prevalence itself.” And “We included TIRET-baseline prevalence as a covariate. When comparing communities that had been randomized to different treatments pre-TIRET (in TANA), we did not include TIRET-baseline as a covariate as this would be a post-randomization correction.”
